# Stable high-capacity and high-rate silicon-based lithium battery anodes upon two-dimensional covalent encapsulation

**DOI:** 10.1038/s41467-020-17686-4

**Published:** 2020-07-31

**Authors:** Xinghao Zhang, Denghui Wang, Xiongying Qiu, Yingjie Ma, Debin Kong, Klaus Müllen, Xianglong Li, Linjie Zhi

**Affiliations:** 1 CAS Key Laboratory of Nanosystem and Hierarchical Fabrication, CAS Center for Excellence in Nanoscience, National Center for Nanoscience and Technology, Beijing, 100190 China; 2 University of Chinese Academy of Sciences, Beijing, 100049 China; 3 Max Planck Institute for Polymer Research, Mainz, 55128 Germany

**Keywords:** Batteries, Synthesis of graphene

## Abstract

Silicon is a promising anode material for lithium-ion and post lithium-ion batteries but suffers from a large volume change upon lithiation and delithiation. The resulting instabilities of bulk and interfacial structures severely hamper performance and obstruct practical use. Stability improvements have been achieved, although at the expense of rate capability. Herein, a protocol is developed which we describe as two-dimensional covalent encapsulation. Two-dimensional, covalently bound silicon-carbon hybrids serve as proof-of-concept of a new material design. Their high reversibility, capacity and rate capability furnish a remarkable level of integrated performances when referred to weight, volume and area. Different from existing strategies, the two-dimensional covalent binding creates a robust and efficient contact between the silicon and electrically conductive media, enabling stable and fast electron, as well as ion, transport from and to silicon. As evidenced by interfacial morphology and chemical composition, this design profoundly changes the interface between silicon and the electrolyte, securing the as-created contact to persist upon cycling. Combined with a simple, facile and scalable manufacturing process, this study opens a new avenue to stabilize silicon without sacrificing other device parameters. The results hold great promise for both further rational improvement and mass production of advanced energy storage materials.

## Abstract (web summary)

Stabilizing silicon without sacrificing other device parameters is essential for practical use in lithium and post lithium battery anodes. Here, the authors show the skin-like two-dimensional covalent encapsulation furnishing a remarkable level of integrated lithium storage performances of silicon.

## Introduction

**Fig. 1:**
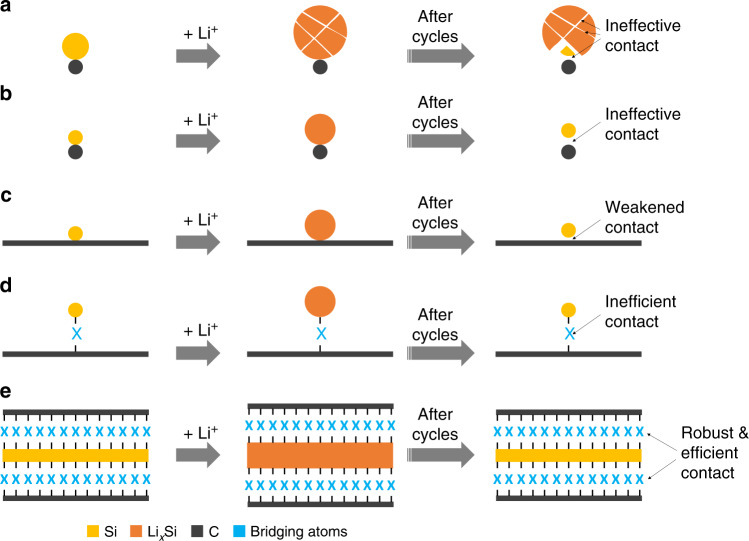
Binding strategies of silicon with electrically conductive media. **a** Point-mode physical binding of Si with a conventional conductive medium (e.g., carbon black). The large volume change causes pulverization of Si obstructing the contact and induces side reactions, as well as SEI propagation further deteriorating the contact. The ineffective binding or unbinding of Si results in poor cycling stability. **b** Downsizing of Si. Although resistant to mechanical fracture, the point-mode physical contact is similarly prone to become ineffective, hindering improvement in cycling and rate capability. **c** Tailoring of the adjacent conductive medium. With the Si/C combination to accommodate the volume change of Si, the cycling stability is improved; yet, this is at the expense of rate capability because the easily weakened point-mode physical binding is still adopted in most cases. **d** Manipulation of the binding between Si and the adjacent conductive medium. As the point mode of covalent binding is inefficient and unfavorable for the rate capability, the existing covalent binding still encounters risks of being disrupted due to the direct contact and propagating erosion of Si with the electrolyte. **e** Designing two-dimensional covalent binding for Si. This skin-like binding creates and more importantly, secures a robust and efficient contact between Si and C components, granting stable and fast electron/ion transport from/to the Si upon cycling. It is noteworthy that Li^+^ is defined as lithium ions.

To meet the ever-demanding performance requirements of lithium-ion batteries (LIBs) and post-lithium rechargeable batteries for applications such as powering electric vehicles and integrating intermittent renewable energy, high-capacity electrochemically active electrode materials are being extensively exploited^1–8^. The binding between such electrode materials and the adjacent electrically conductive media (e.g., carbon black) and consequently the electrode framework is a critical issue^9–12^, in particular when employing conventional electrode formulation with known conductive additives and binders^13^. This electrical connection, at the same time, must be resistant to the electrolyte of a battery cell. The high-capacity active materials inevitably suffer from large volume changes during charging and discharging processes (Fig. 1a). Thus, silicon possesses the highest theoretical gravimetric (specific) capacity, which is ten times that of commercial graphite (372 mAh g^−1^), but experiences up to 300% volume change upon lithiation and delithiation^6,14^. Such a large volume change causes pulverization and electrical disconnection of the active material^15^, but also forms dynamic interfaces^9,16–19^. This “dynamic interfacing” is prone of worsening or blocking the electrical contact of the material by giving rise to undesirable side reactions between active material and electrolyte. This, in turn, would cause propagation and thickening of a so-called solid electrolyte interphase (SEI) layer and rapid deterioration of the capacity and the cycle life.

So far, a variety of design strategies has been developed for silicon which can be classified into three categories as follows: (i) downsizing of silicon (Fig. 1b) to feature sizes on the nanoscale (especially below the critical value) can provide materials surviving operation without mechanical fracture. Although discrete nanoparticles^15,20,21^, nanowires^6,22,23^, nanotubes^24,25^, nanosheets^26–28^, and porous sponges^29–31^ have been employed, the issue of dynamic interfacing remains unsolved and, in many cases, even becomes more severe. Thereby, the contact between silicon and the adjacent conductive medium, specifically via single- or few-point physical binding, becomes ineffective and compromises cycle stability and rate capability; (ii) tailoring of the adjacent electrically conductive medium (Fig. 1c) by a combination of the downsized silicon with carbon nanostructures typically as yolk-shell or wire-in-tube structures. Thereby, the large volume change of silicon is accommodated and the interfacing with electrically conductive media and electrolyte is secured by the stable voids. Cycling stability is greatly improved, however, at the expense of rate capability. The reason for this shortcoming is that the single- or few-point physical binding retards and even obstructs charge transport from and to the silicon^32–44^; (iii) covalent binding between the downsized silicon and adjacent electrically conductive media with the greatest potential to reduce accidental disconnection (Fig. 1d). Yet, this binding furnishes an inefficient point mode, incapable of substantially enhancing charge transport kinetics^24,45–48^. Moreover, it encounters risks of disruption upon electrolyte erosion. Taken all these conflicting requirements together, covalent binding of silicon appears as the method of choice for energy storage materials with large volume change, but is in need of conceptually new design principles.

**Fig. 2:**
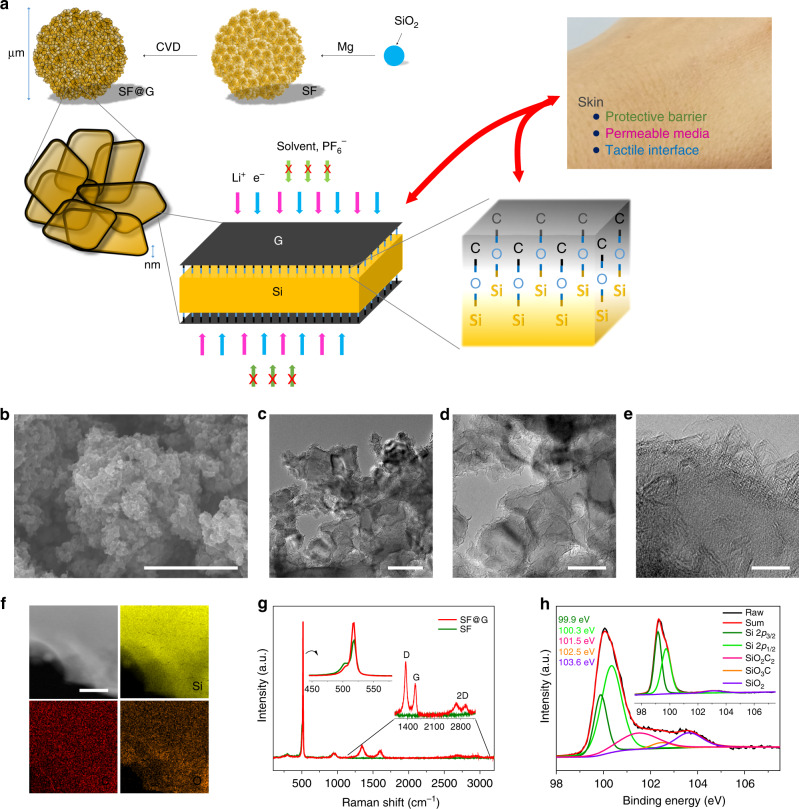
Fabrication and characterization of SF@G. **a** Schematic of the fabrication process for SF@G. The synthesized SF@G features a two-dimensional covalently bound component interface, enabling stable and fast electron (e^−^) and lithium-ion (Li^+^) transport, while fundamentally blocking undesirable substances such as the electrolyte solvents (EC, DEC) and anions (PF_6_^−^), mimicking the skin functions typically as a protective barrier, a permeable media, and a tactile interface. **b** SEM image of SF@G. **c**–**e** TEM images of SF@G. **f** Scanning transmission electron microscopy (STEM) and elemental mapping images of SF@G. **g** Raman spectra of SF@G and SF with magnified regions in the insets. **h** Si 2*p* XPS spectrum of SF@G along with that of SF in the inset, indicating the covalent binding at the Si/C component interface of SF@G. Scale bars, 2 µm (**b**), 100 nm (**c**), 50 nm (**d**), 10 nm (**e**), and 100 nm (**f**).

Herein, we show a (skin-like) covalent encapsulation of silicon (Figs. 1e and 2a). As a proof-of-concept, two-dimensional covalently bound Si-C hybrid materials (namely, SF@G) are shown to exhibit stable, high-capacity, and high-rate lithium storage properties with respect to weight, volume, and area. Such a high level of integrated performance is markedly superior to previous literature studies. As a key issue, the binding between Si and C establishes a robust and efficient contact, and thus enables fast electron as well as ion transport from and to silicon. More importantly, as proven by interfacial morphology and chemical composition, this skin-like binding drastically changes the interface of silicon with the electrolyte and thus renders it stable upon cycling. Our material prototype unlocks the potential of the covalent encapsulation. Markedly different from previously reported concepts for silicon anodes where either physical binding or single- and few-point covalent binding have been adopted, our approach realizes a new binding mode between silicon and the adjacent electrically conductive media towards exploiting silicon-based lithium battery anodes with high integrated performance.

## Results

### Fabrication and characterization

The SF@G material was synthesized by magnesium reduction of silicon dioxide to produce silicene flowers (SF) onto which graphene (G) was deposited by chemical vapor deposition (CVD) (Fig. 2a). Scanning electron microscopy (SEM), transmission electron microscopy (TEM), and elemental mapping images (Fig. 2b–f and Supplementary Fig. 1) reveal the morphology of SF@G. SF@G possesses a micro-sized hydrangea flower-like architecture composed of many interconnected nanoplates. On the surface of every nanoplate, graphene nanosheets (typically, 2~3 layers) are observed to be conformally deposited, in line with investigations of tap density, specific surface area, pore size distribution, as well as graphene morphology (Supplementary Figs. 2–4). The Raman spectrum (Fig. 2g) of SF@G displays an intense peak at 517 cm^−1^ with a shoulder at a lower frequency, confirming the intact nanoplates upon the CVD process^27^. Furthermore, the ratio of the D band to G band is estimated to be around 1.5, revealing the presence of pinholes, defects, and/or disordered domains in the deposited graphene favorable for ion transport (Fig. 2g and Supplementary Fig. 5). In addition to the characteristic peaks of Si (99.9, 100.3 eV) and the native SiO_2_ layer (103.6 eV), the X-ray photoelectron spectroscopy (XPS) spectrum of the Si 2*p* region of SF@G exhibits two additional bands at 101.5 and 102.5 eV (Fig. 2h), in line with C 1*s* and O 1*s* results (Supplementary Fig. 6). These peaks can be attributed to interfacial Si–O–C bonds between two-dimensional Si nanoplates and G nanosheets. Due to the conformal deposition of graphene and subsequent area-to-area contact between Si and C components, as verified by SEM and TEM observations, this covalent binding exists in a two-dimensional mode. The covalent binding at the interface of two-dimensional components can be described by a tentative chemical structure of SF@G (Supplementary Fig. 7). Also relevant is the absence of Si–O–C signals in the hydrofluoric acid (HF)-treated SF@G (SF@G-HF) (Supplementary Fig. 8). The “skin-formation” is suggested to be associated with the native silicon oxide layer on the Si nanoplates (Supplementary Fig. 9). Upon interfacing with the introduced hydrogen gas, the silicon oxide on Si nanoplates is partially reduced to Si–O intermediates at high temperature. These intermediates are further combined with carbon species derived from methane, while additional supply of methane furnishes the deposition of graphene. Unless otherwise noted, the Si content in SF@G is about 88% as estimated by thermogravimetric analysis (Supplementary Fig. 10).

### Electrochemical performance and kinetic characteristics

**Fig. 3:**
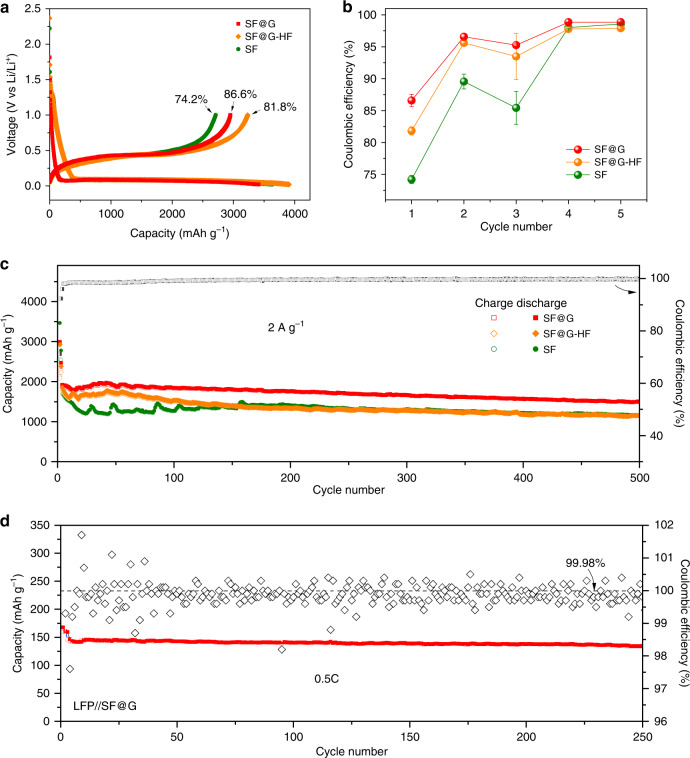
Electrochemical characterization of SF@G upon cycling. **a** Charge–discharge profiles of SF@G, SF@G-HF, and SF at 0.2 A g^−1^ for the first cycle. **b** Coulombic efficiency of SF@G, SF@G-HF, and SF for initial five cycles. **c** Cycling performance of the SF@G and control electrodes over 500 cycles at 0.2 A g^−1^ for initial two cycles and 2 A g^−1^ for subsequent cycles. The Coulombic efficiency of SF@G is plotted on the secondary y-axis. **d** Reversible capacity and Coulombic efficiency vs. cycle plots of the LFP//SF@G full cell with SF@G as the anode and a commercial lithium iron phosphate (LFP) as the cathode (it is noteworthy that the capacity is based on the weight of active materials in the cathode).

**Fig. 4:**
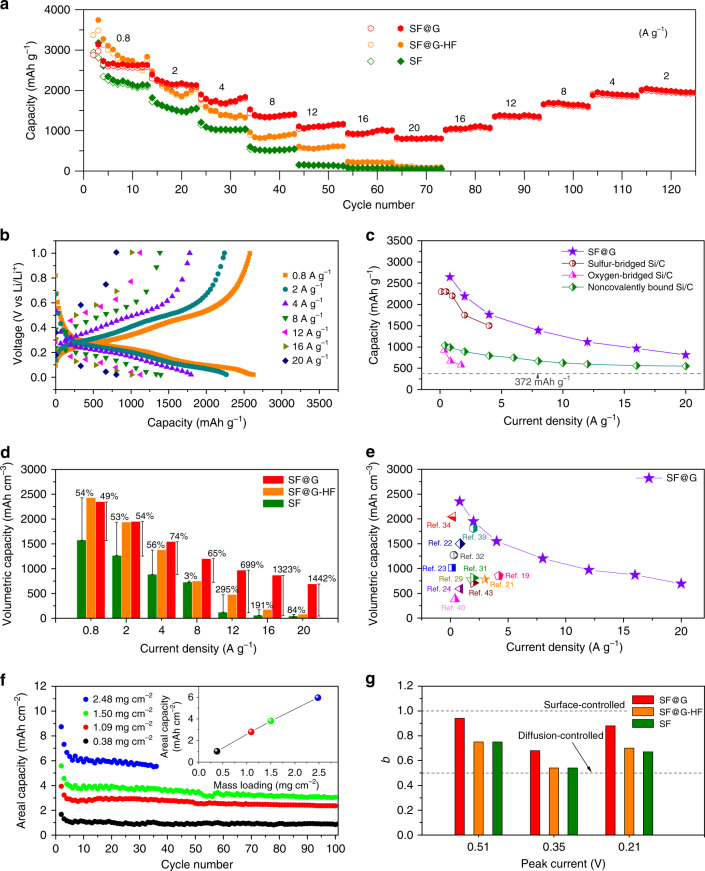
Electrochemical characteristics of SF@G tested at different rates. **a** Capacity of SF@G, SF@G-HF, and SF cycled at different rates from 0.8 to 20 A g^−1^ (ten cycles for each rate). **b** Galvanostatic charge/discharge profiles of SF@G at various rates. **c** Capacity of SF@G at annotated rates, comparing with typical Si/C anodes with conventional interfacial binding modes as noted. **d** Volumetric capacity of SF@G, SF@G-HF, and SF at various rates. **e** Volumetric capacity at annotated current rates for SF@G with some representative Si anodes reported in the literatures as noted. **f** Areal capacity of SF@G at various active material mass loadings. **g** The *b*-value of anodic (0.51 V and 0.35 V) and cathodic (0.21 V) peaks for SF@G, SF@G-HF, and SF, derived from cyclic voltammetry (CV) experiments at various sweep rates.

The achieved covalent, “layered” encapsulation of SF@G affords a remarkable battery performance (Figs. 3 and 4). As exhibited in Fig. 3a, b and Supplementary Fig. 11, SF@G demonstrates an improved initial Coulombic efficiency (87%) and a rapid increase of stabilized Coulombic efficiency > 99%, in sharp contrast with SF@G-HF and SF. This significantly enhanced Coulombic efficiency of SF@G depicts the interfacial difference between SF@G and SF@G-HF, although being made from the same components. Irreversible consumption of lithium and subsequent SEI formation can be rigorously prevented in SF@G. Although possessing the similar micro-sized architecture, SF@G also offers a dramatically improved cycling stability at a high rate of 2 A g^−1^ over 500 cycles compared with SF@G-HF and SF, delivering a high specific capacity that is more than five times higher than the theoretical capacity of graphite (Fig. 3c). The excellent cycling stability of SF@G is also verified by a prototype LFP//SF@G full cell device showing both stable cycling and high Coulombic efficiency (Fig. 3d). The capacities of SF@G and control electrodes at various rates (Fig. 4a, b) further demonstrate superb rate capability of the SF@G. The specific capacity of SF@G at rates of 0.8, 2, 4, 8, 12, 16, and 20 A g^−1^ is ~2646, 2194, 1763, 1389, 1119, 967, and 812 mAh g^−1^, respectively. By comparison, both SF@G-HF and SF cannot deliver such a high capacity, especially at larger rates. Even after cycling at very high current rates of up to 20 A g^−1^, the capacity is still reproducable, firmly corroborating the high reversibility and cyclic stability of SF@G. As demonstrated in Fig. 4c, the rate performance of this two-dimensional covalently bound SF@G surpasses that of competing design concepts such as point-mode sulfur-bridged Si/C^45^, point-mode oxygen-bridged Si/C^24^, and noncovalently bound Si/C^37^ (Fig. 4c). Benefiting from the high gravimetric capacity and the high density of the material, the volumetric capacity of SF@G anodes is extraordinarily high (Fig. 4d, e). Considering the whole electrode volume as well as the volume change of 5.8~6.6% upon cycling (Supplementary Fig. 12), SF@G exhibits a volumetric capacity of 2350 mAh cm^−3^ at a rate of 0.8 A g^−1^, which is more than four times that (~550 mAh cm^−3^) of commercial graphite anodes. Even at high rates of 2, 4, 8, 12, 16, and 20 A g^−1^, a volumetric capacity of 1952, 1547, 1202, 971, 869, and 694 mAh cm^−3^ is delivered, respectively, which is 54%, 74%, 65%, 699%, 1323%, and 1442% of that of SF at the same rates. The achieved volumetric capacity of SF@G is markedly superior to previous results for different silicon anodes^19,21–24,29,31,32,34,39,40,43^ (Fig. 4e). Furthermore, the areal capacity of SF@G can be adjusted almost proportional to the mass loading of the active material, and a reversible areal capacity of ~6 mAh cm^−2^ is reached at a mass loading of 2.48 mg cm^−2^ (Fig. 4f), much higher than that of a commercial LIB cell. In addition, the viability of SF@G is also characterized by a competitive energy density (Supplementary Table 1 and Supplementary Note 1). The high reversibility, high capacity, and high rate capability of SF@G reflect stable and fast electron and ion transport from and to the silicon, together with favorable lithium storage kinetics. These properties primarily stem from a robust and efficient contact between silicon and graphene at both sides of each nanoplate due to the covalent encapsulation and consequent two-dimensional tight binding between Si and C, although the specific surface area may be an additional factor. This scenario is nicely supported by the Nyquist plots obtained from electrochemical impedance spectroscopy (EIS) (Supplementary Fig. 13) and further validated by the significantly improved *b*-values (defining the relation of peak current to sweep rate)^37^ of both cathodic (0.21 V) and anodic (0.35 and 0.51 V) peaks of SF@G in comparison with SF@G-HF and SF (Fig. 4g, Supplementary Fig. 14, and Supplementary Note 2).

### Interfacial morphology and chemical composition upon cycling

**Fig. 5:**
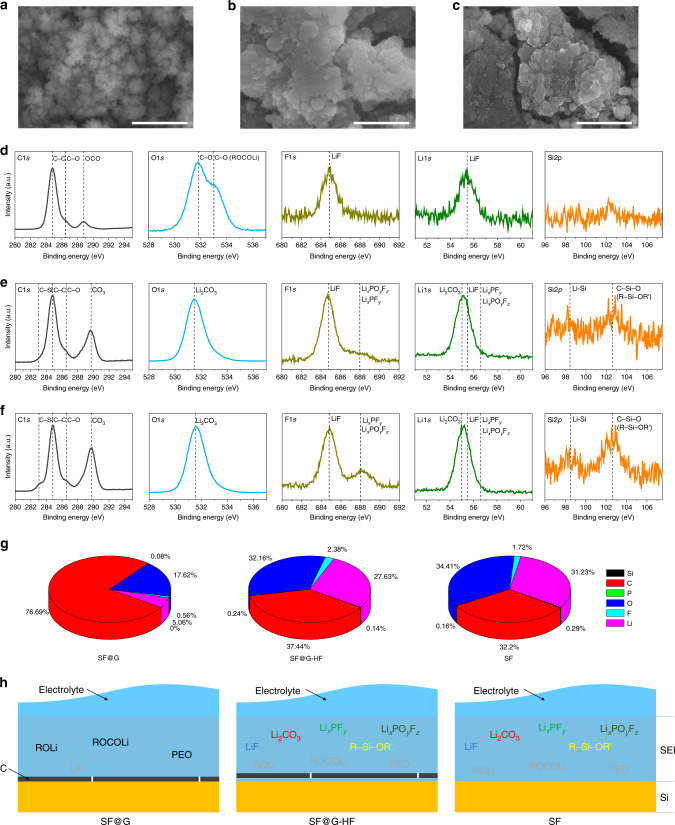
Interfacial morphology and chemical composition after cycling. **a**–**c** SEM images of (**a**) SF@G, (**b**) SF@G-HF, and (**c**) SF after 100 cycles. **d**–**f** C 1*s*, O 1*s*, F 1*s*, Li 1*s*, and Si 2*p* XPS spectra of (**d**) cycled SF@G, (**e**) cycled SF@G-HF, and (**f**) cycled SF. **g** Interfacial atom concentrations of cycled SF@G, SF@G-HF, and SF. **h** Schematic description of the interface of cycled SF@G, SF@G-HF, and SF, showing distinctly different components as the majority of cycled SF@G and control samples. It is noteworthy that the components annotated in gray represent the minority and the SEI thickness is not scaled. Scale bars, 2 µm (**a**–**c**).

To unravel the structural origin of the achieved performance, interfacial morphology and chemical composition of the cycled SF@G and control samples have been investigated. The SEM and TEM images of SF@G, SF@G-HF, and SF after 100 cycles are displayed in Fig. 5a–c and Supplementary Figs. 15 and 16. It is obvious that the SF@G retains the original flower-like architecture upon cycling, possessing a thin and smooth interfacial morphology. Different from that, the flower-like architecture of cycled SF@G-HF is blurred, with an abundance of rather rough SEI deposits filling up the gaps between SF@G-HF nanoplates. In case of cycled SF, the flower-like appearance is nearly completely deformed. The XPS results further depict interfacial SEI components of cycled SF@G, SF@G-HF, and SF (Fig. 5d–f and Supplementary Figs. 17 and 18). As shown in Fig. 5d, the C 1*s* XPS spectrum of cycled SF@G reveals peaks assignable to C–C, C–O, and O = C–O (OCO) bonds, respectively. The C–O and OCO bonds are confirmed by the detection of appropriate binding energy positions in the O 1*s* spectrum of SF@G, respectively. These results imply the presence of polyethylene oxide (PEO) and lithium alkoxides (ROLi) and carboxylates (ROCOLi)^49–51^ as typical SEI components in the cycled SF@G. Although the similar peaks corresponding to C–C and C–O bonds are still present, a prominent peak appears at 289.7 eV in the cases of SF@G-HF and SF (Fig. 5e, f), attributable to carbonate-containing species (labeled as CO_3_). Their O 1*s* spectra suggest the presence of Li_2_CO_3_ as the major SEI component in the cycled SF@G-HF and SF. These features clearly characterize the material interface, at which electrochemical reduction and consumption of the electrolyte solvents (ethylene carbonate (EC) and diethyl carbonate (DEC) in this work) occur (Supplementary Fig. 19 and Supplementary Note 3)^49–51^. Although the SF@G-HF bears a similar electrolyte-interacting interface to that of SF reflecting the direct contact of Si with the electrolyte in both cases, SG@G is obviously different due to the presence of skin-like binding, in agreement with the Coulombic efficiency results. These findings are firmly supported by a series of additional peak assignments such as siloxane structures (R–Si–OR’), LiF and phosphorus compounds (Li_*x*_PF_*y*_ and Li_*x*_PO_*y*_F_*z*_) (see Supplementary Note 4).

The distinct difference in interfacial components is described further by the elemental compositions of the cycled samples (Fig. 5g). In the cycled SF@G, the majority is C (~77 at%), along with a low concentration of O and Li, as well as an insignificant concentration of F and P, proving the organic-dominated nature of SEI in SF@G. In comparison with SF@G, the cycled SF@G-HF and SF display similar atom concentrations corresponding to their inorganics-dominated SEI, where the concentration of C is substantially decreased to ~37% and ~32 at%, respectively, with the concentration of O, Li, F, P, and Si significantly increased. The distinctly high concentration of O and Li in both SF@G-HF and SF points toward Li_2_CO_3_ as a major component. As schematically shown in Fig. 5h, the main interfacial ingredients of cycled SF@G-HF and SF consistently include Li_2_CO_3_, LiF, Li_*x*_PF_*y*_, Li_*x*_PO_*y*_F_*z*_, and R–Si–OR’; the interface of cycled SF@G mostly consists of organic species (e.g., PEO, ROLi, and ROCOLi), with an almost negligible amount of inorganic materials. After washing with 5% hydrochloric acid (HCl), the cycled SF@G exhibits an analogous interfacial morphology and elemental composition (e.g., C, O) to its unwashed one, whereas the cycled SF@G-HF and SF show a scenario totally different from unwashed counterparts (Supplementary Figs. 17 and 20–22), reflecting stable characteristics of the SF@G interface. The differences discussed above are also revealed by elemental compositions obtained from EDX analyses (Supplementary Fig. 23 and Supplementary Note 5). This interfacial contact stability is validated by EIS results (Supplementary Fig. 24). It is noteworthy that the covalent binding present in SF@G is well retained, whereas its two-dimensional hybrid structure persists upon cycling (Supplementary Figs. 20, 25, and 26, and Supplementary Note 6). It should be noted as well that this structural and interfacial stabilization, combined with minimized electrode thickness variations (Supplementary Fig. 12), point toward better accommodation of the volume change of silicon in SF@G. This can be attributed to the two-dimensional character (mimicking the behavior of a planar thin film) and three-dimensional spatial orientation (creating free spaces to accommodate the volume change) of the involved nanoplates^27^. The similar interfacial morphology and chemical composition of cycled SF@G-HF and SF disclose an insufficient ability of the carbon hybridized with silicon in a two-dimensional noncovalent manner for blocking the direct contact of Si with the electrolyte. Specifically, in the case of SF@G-HF, graphene (G) cannot effectively inhibit undesirable side reactions between Si and electrolyte and consequent SEI propagation upon cycling, even if a sandwich-like hybrid structure is adopted to mitigate the structural and interfacial variation of Si upon lithiation and delithiation. One expects that the electrolyte penetrates through pinholes and/or defects of G and interacts with the dynamic Si interface. By contrast, the marked difference in interfacial morphology and composition of cycled SF@G provides firm evidence of the critically important role of skin-like covalent binding in our concept to fundamentally block direct contact of Si with the electrolyte, confer a thin and stable SEI, and maintain the as-established contact during cycling.

## Discussion

In conclusion, a design strategy for skin-like covalent encapsulation of silicon electrodes is developed to address the issue of its large volume change. The proof-of-concept of two-dimensional, covalently bound silicon-carbon hybrids exhibits stable high-capacity and high-rate lithium storage performances when referred to weight, volume and area. These outstanding results are superior to previous investigations. Markedly distinct from existing techniques of battery fabrication, the involved two-dimensional, covalent binding creates a robust and efficient contact between the silicon and electrically conductive media, enabling stable and fast electron as well as ion transport from and to the silicon. As certified by distinctive interfacial morphology and binding modes between elements, this encapsulation rigorously blocks the direct contact of silicon with the electrolyte and changes the material interface, making the contacts persistent to cycling. Combined with a cost-effective raw material and a simple, facile, and scalable manufacturing process, the study opens a new and viable avenue to stabilize silicon without sacrificing parameters including capacity and rate capability. Further, our work can stimulate protocols for the rational design and mass production of other advanced energy materials to be used in lithium storage and beyond.

## Methods

### Preparation of SF@G

The SF@G was fabricated by magnesium reduction and CVD processes as schematically shown in Fig. 2a. Briefly, the freshly prepared SF, through magnesium reduction of silicon dioxide (SiO_2_) as previously reported^27^, was placed in a quartz vessel, which was heated at a ramp rate of 5 °C min^−1^ to 1050 °C in a horizontal tube furnace under argon/hydrogen (Ar/H_2_; 1:1) atmosphere. The Ar flow was then turned off, the H_2_ flow was maintained at 100 standard cubic centimeters (s.c.c.m.), and 100 s.c.c.m. of methane (CH_4_) was introduced into the reaction tube and kept for 10 min. Cooling of the sample to room temperature under the protection of Ar and H_2_ furnished SF@G. The SF@G-HF control sample was obtained by immersing the as-prepared SF@G in 5% HF solution for a defined time (typically, 1 h) to break the covalent binding at the Si/C interface, followed by repeated washing and subsequent drying. In addition, graphene was obtained by removing the silicon of SF@G with 5% sodium hydroxide (NaOH) aqueous solution under 80 °C.

### Material characterization

The morphology and structure of all samples were investigated by FE-SEM (Hitachi S4800) and FE-TEM (FEI Tecnai G2 20 STWIN and Tecnai G2 F20 U-TWIN). Raman spectra were collected using a Renishaw inVia Raman microscope with a laser wavelength of 514.5 nm. XPS measurements were performed on an ESCALAB250Xi apparatus with an Al Kα X-ray source. Before the XPS measurements, the cycled samples were washed repeatedly with fresh dimethyl carbonate to remove the residual electrolyte; in some cases, the cleaned samples were subjected to further washing with 5% HCl to remove most unstable SEI components of the samples if there exist. Nitrogen adsorption/desorption isotherms were measured at 77 K with an ASAP 2020 physisorption analyzer. The Brunauer–Emmett–Teller method and Barrett–Joyner–Halenda model were utilized to estimate the specific surface area and pore size distribution, respectively.

### Electrochemical characterization

The working electrodes were made by a typical slurry method with active materials (SF@G, SF@G, or SF), conductive additive (Super P, Alfa Aesar), and polyacrylic acid (weight-average molecular weight of 240,000, Alfa Aesar) binder at a mass ratio of 8:1:1. Unless otherwise specified, the typical mass loading of active materials was 1.0~2.5 mg cm^−1^. All the electrodes were degassed in vacuum at 60 °C for at least 2 h before use. Two-electrode CR2032 coin-type half-cells were assembled in an argon-filled glove box (<0.1 p.p.m. of oxygen and water) with lithium foil as the counter electrode. The electrolyte was 1.0 M LiPF_6_ in 1:1 (v/v) EC/DEC with 5% fluoroethylene carbonate and the separator was porous polypropylene films (Celgard 2400). The full cell was designed with a N/P ratio of ca. 1.05. The cathode electrodes were fabricated by mixing commercial lithium iron phosphate (LFP), carbon black, and polyvinylidene fluoride in *N*-methyl-2-pyrrolidone at a mass ratio of 8:1:1, casting, and drying in vacuum at 80 °C for at least 2 h. Coin-type full cells with the fabricated LFP cathodes and SF@G anodes were assembled in a glove box filled with argon gas. The electrolytes and separators in the full cells were the same as those used in the half cells. Electrochemical tests were carried out in the voltage window between 2.5 and 4.2 V. The cycling and rate capability tests were performed using a CT2001A battery program controlling test system within the voltage range of 0.02–1.0 V. Cyclic voltammetry was carried out in the potential range of 0.02–1.0 V at various rates (0.1~1.0 mV s^−1^) with a CHI660D electrochemical station. Unless otherwise specified, all electrochemical measurements were undertaken at room temperature in half-cells, the capacity reported was based on the total weight of active materials in the working electrode, as well as the annotated cycling conditions. The volumetric capacity was calculated as product of gravimetric capacity and packing density of the electrode, and the coulombic efficiency calculated using the ratio of delithiation capacity to lithiation capacity.

## Supplementary information

Supplementary Information

Peer Review File

## Data Availability

The data supporting the findings of this study are available from the corresponding author upon reasonable request.
