# Supplementary material for: Stable high-capacity and high-rate silicon-based lithium battery anodes upon two-dimensional covalent encapsulation
Source: Nat Commun. 2020 Jul 31;11:3826. doi: 10.1038/s41467-020-17686-4 (PMC7395733; doi:10.1038/s41467-020-17686-4)
Supplement: Supplementary file 1 — Supplementary Information [file 41467_2020_17686_MOESM1_ESM.pdf]

Supplementary Information

Stable high-capacity and high-rate silicon-based lithium battery anodes upon two-dimensional covalent encapsulation

Zhang et al.

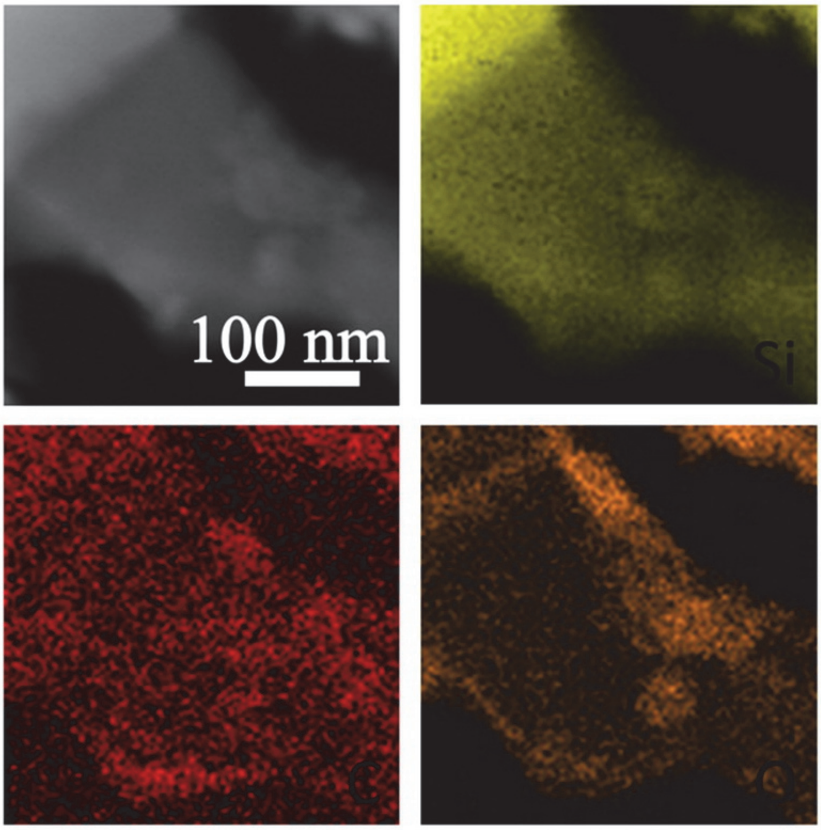

Supplementary Figure 1: Scanning transmission electron microscopy (STEM) and elemental mapping images of a SF@G nanoplate.

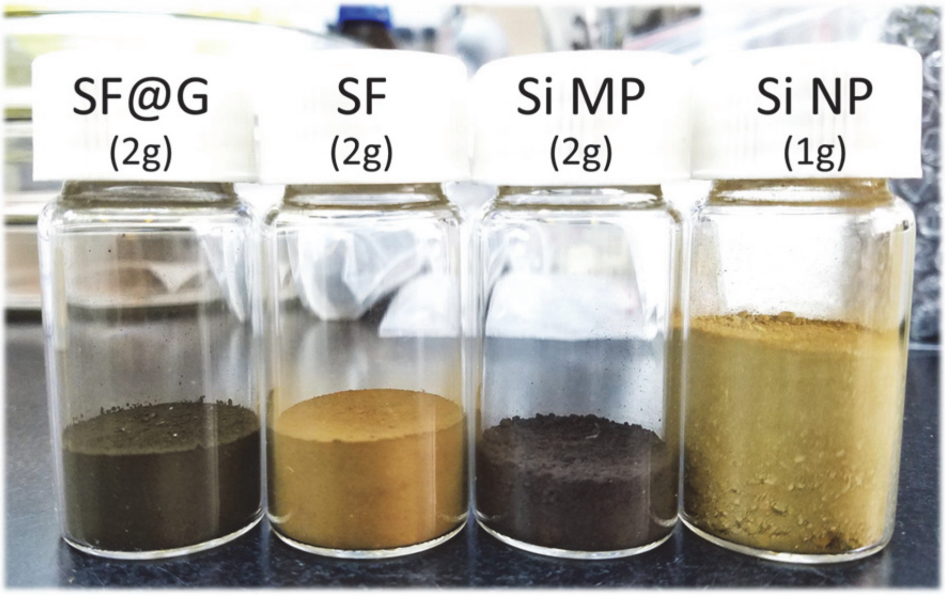

Supplementary Figure 2: Photographs of SF@G, SF, silicon microparticles (Si MP), and silicon nanoparticles (Si NP). Note: the mass of the samples from left to right is 2.0, 2.0, 2.0, and 1.0 g, respectively. The SF@G shows a similar tap density (ca. 0.9 g cm^{-3}) to that of SF, indicative of the conformal deposition of G on Si nanoplates in the SF@G. Furthermore, the density of SF@G is approximately four times that of Si NP, being evidently favorable for volumetric capacity and critical for practical applications.

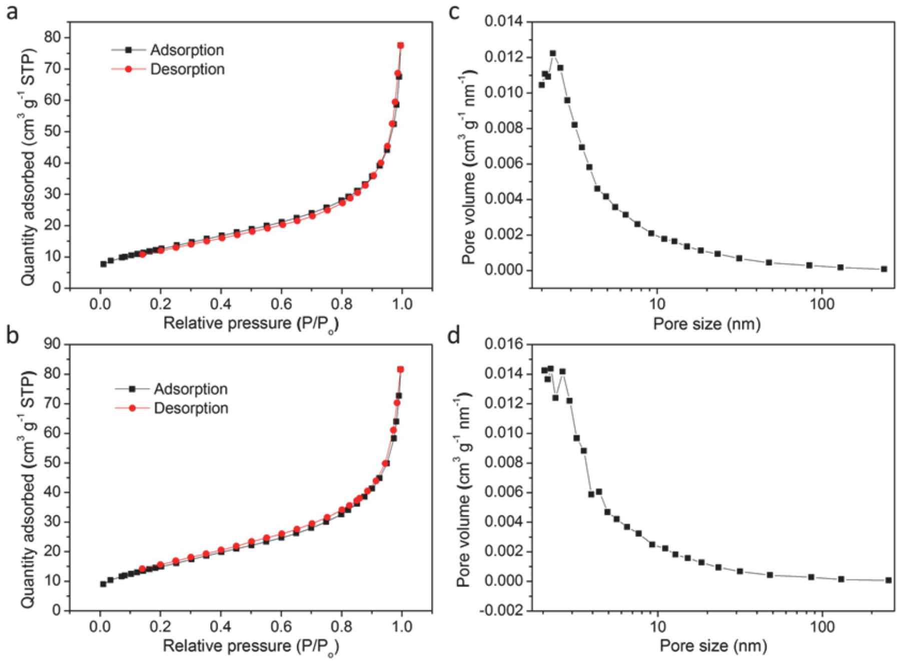

Supplementary Figure 3: **a**,**b**, Nitrogen adsorption-desorption isotherms of (**a**) SF@G and (**b**) SF@G-HF. **c**,**d**, Pore size distributions of (**c**) SF@G and (**d**) SF@G-HF. The Brunauer-Emmett-Teller (BET) method and Barrett-Joyner-Halenda (BJH) model were utilized to estimate the specific surface area and pore size distribution, respectively. The results show the specific surface area of SF@G and SF@G-HF to be 45.7 and 54.5 m^2 g^{-1}, respectively. The similar specific surface area of SF@G to that (~44 m^2 g^{-1})[1] of SF reveals the conformal deposition of G on Si nanoplates in the SF@G. Furthermore, a slightly higher surface area of SF@G-HF than that of SF@G can be attributed to the noncovalent binding between the two components of SF@G-HF. In addition, the similar pore size distribution for SF@G, SF@G-HF, and SF implies that both the CVD process and the HF treatment have a negligible effect on their flower-like architecture composed of different spatial orientations of interconnected nanoplates.

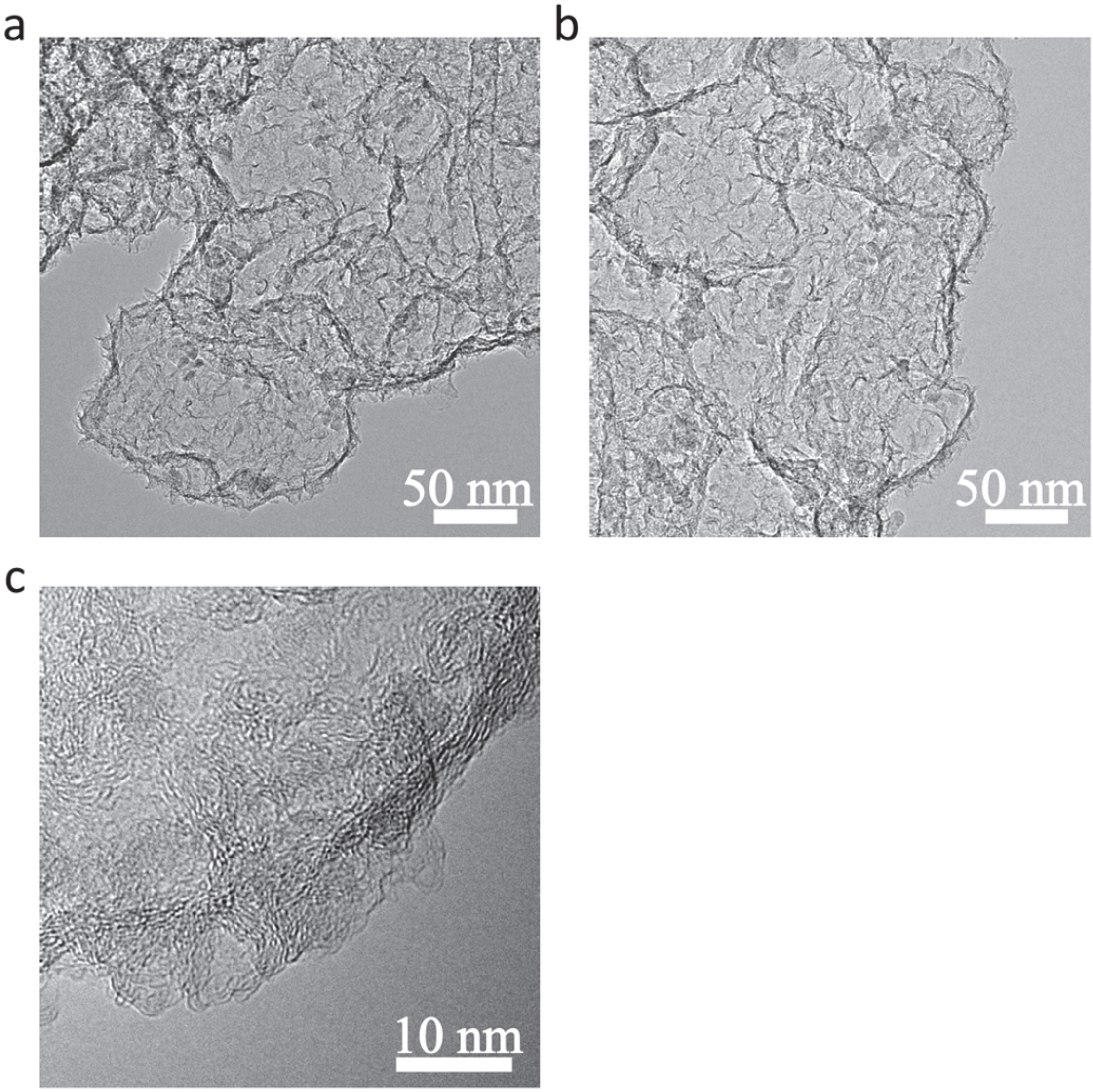

Supplementary Figure 4: a-d, TEM images of graphene (G) obtained by removing the silicon of SF@G with 5% sodium hydroxide (NaOH) aqueous solution under 80 ℃. The modality of G mimics that of Si nanoplates, validating its conformal deposition on the nanoplate.

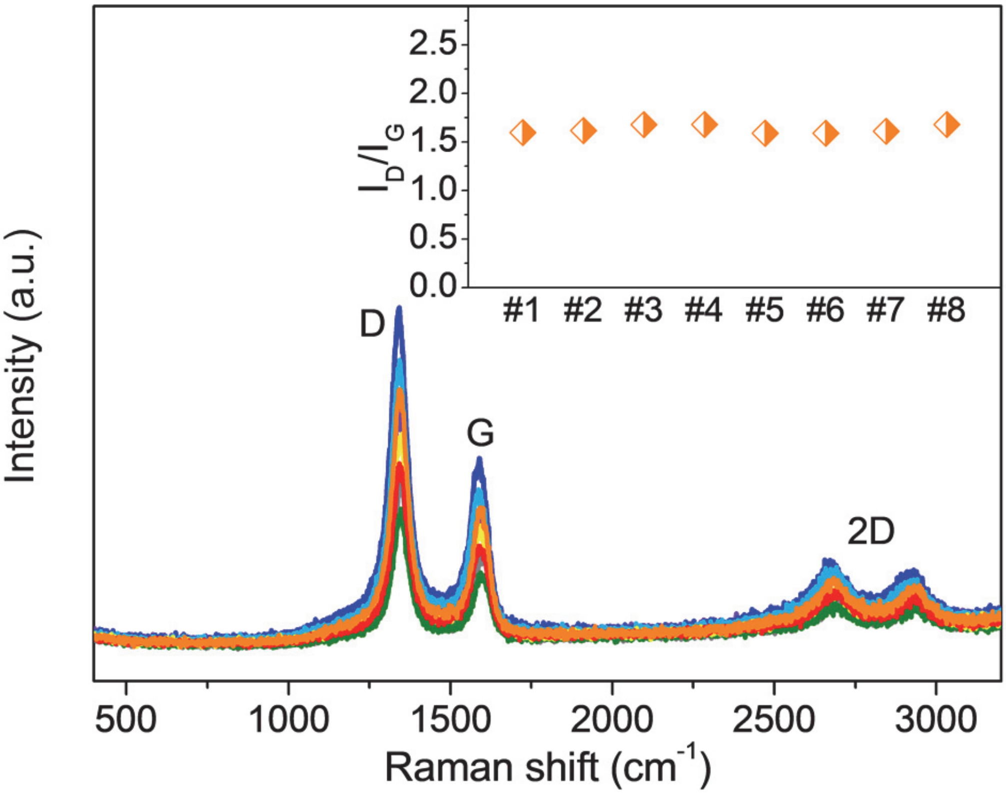

Supplementary Figure 5: Raman spectra of G. The inset shows the ratio of D band to G band (I_D/I_G) to be around 1.5.

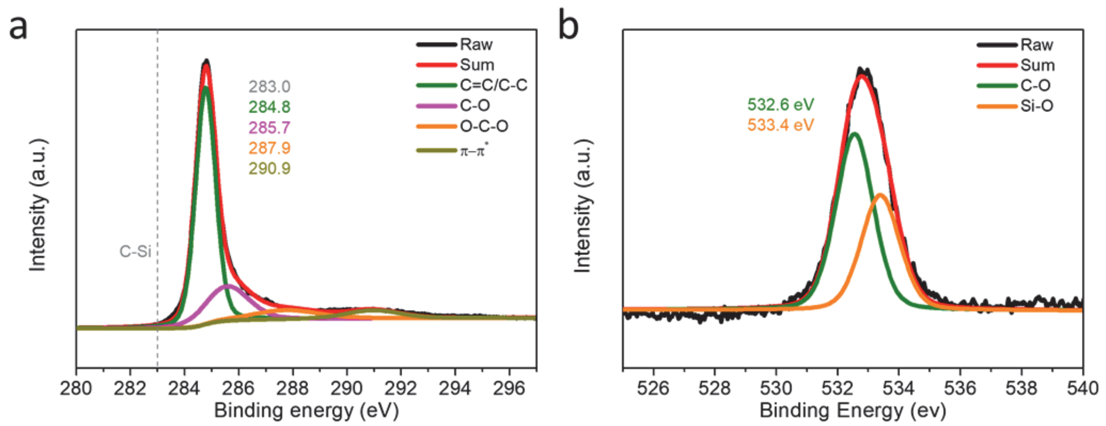

Supplementary Figure 6: XPS spectra of SF@G. **a**, C 1s. **b**, O 1s. In the C 1s XPS spectrum of SF@G, the peaks at 284.8 eV, 285.7 eV, 287.9 eV, and 290.9 eV can be assigned to C–C/C=C, C–O, O–C–O, and π–π^* bonds in graphene, respectively. The observed C–O and O–C–O bonds, combined with C–O and Si–O bonds located at 532.6 eV and 533.4 eV in the O 1s spectra, respectively, indicate the presence of Si–O–C bonds in SF@G, in agreement with the assignments of Si 2p peaks of SF@G. Note that there is no distinguishable peak at 283.0 eV, indicating the absence of C–Si bonds and consequent silicon carbide (SiC) in the SF@G.

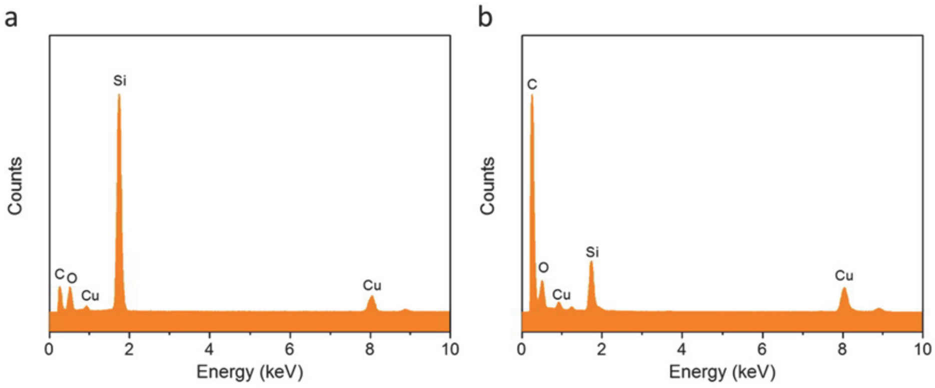

c

	SF@G		G	
	Weight %	Atomic %	Weight %	Atomic %
C(K)	11.80	23.22	85.27	90.68
O(K)	4.09	6.05	7.63	6.09
Si(K)	84.09	70.72	7.08	3.22

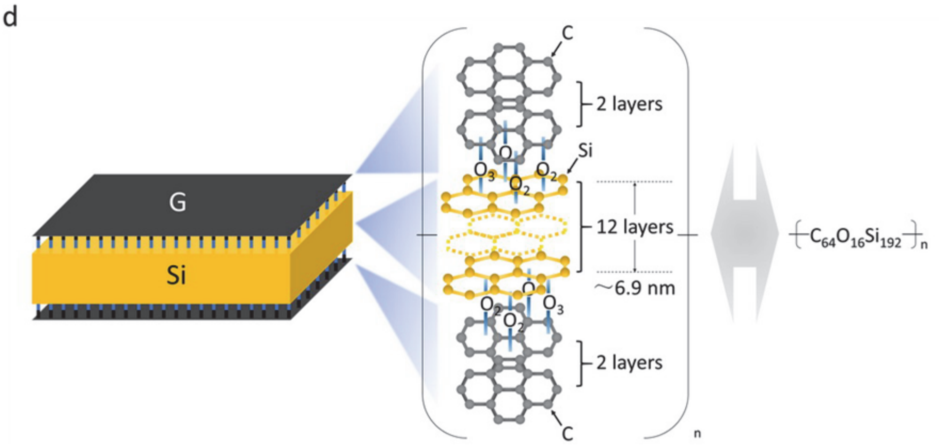

Supplementary Figure 7: **a**,**b**, Energy dispersive X-ray (EDX) patterns of (**a**) SF@G and (**b**) G. **c**, Weight and atomic ratios of SF@G and G. **d**, A speculative chemical structure of SF@G. Based on the atomic ratio of C, O and Si obtained by EDX as well as the layer number of graphene observed from TEM images, it can be tentatively assumed that, in an SF@G nanoplate, two graphene nanosheets are covalently bound through oxygen atoms to both sides of a Si nanoplate with a thickness of 6.9 nm, corresponding to a possible molecular formula of $C_{64}O_{16}Si_{192}$. Notably, the thus-deduced thickness of 6.9 nm is very close to the average thickness (7.2 nm)[1] of the original nanoplates.

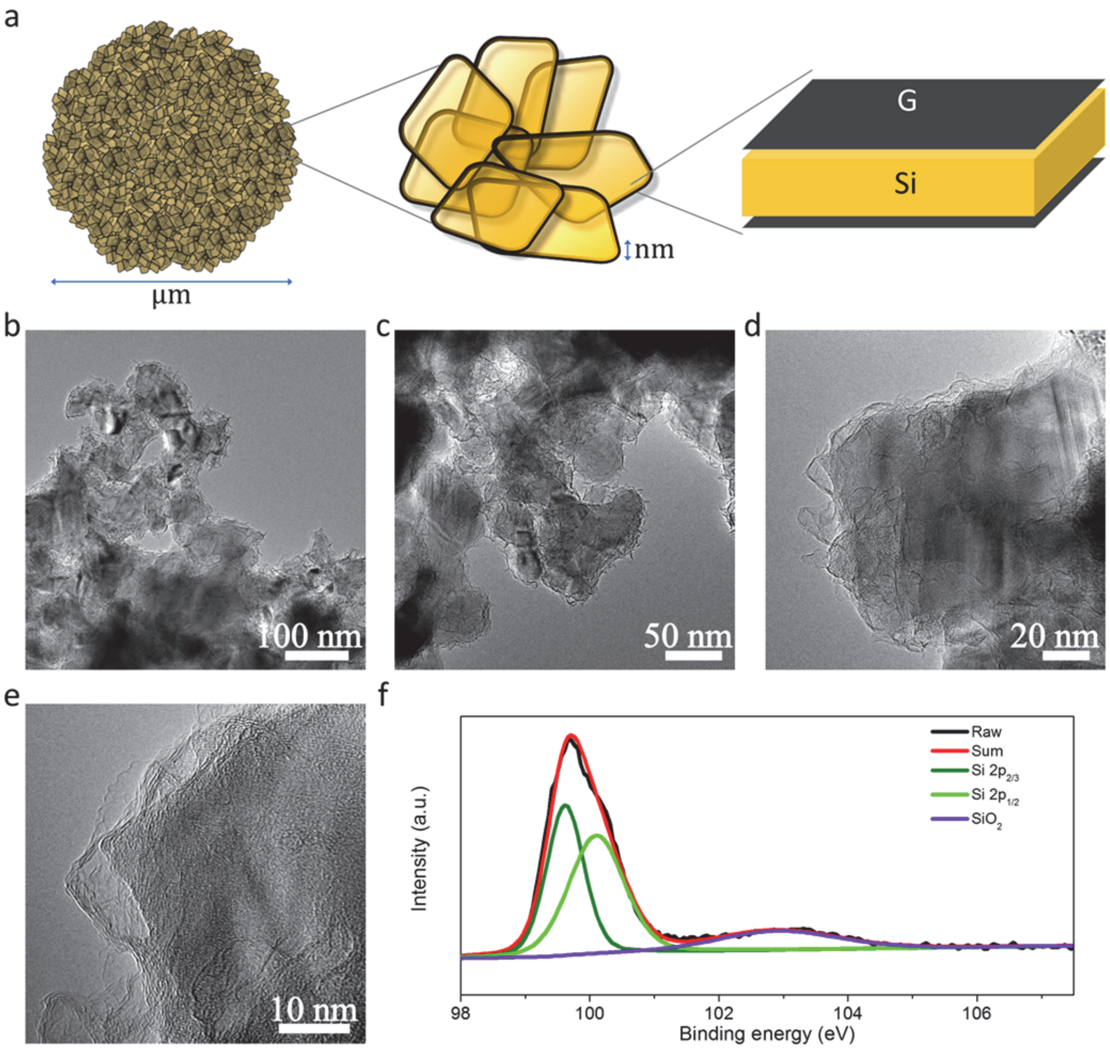

Supplementary Figure 8: Characterization of SF@G-HF. **a**, schematically describing the structure. **b-e**, TEM images. **f**, Si 2p XPS spectrum. As the SF@G-HF shows a similar modality to that of SF@G, the Si–O–C signals corresponding to the covalent binding are absent in the Si 2p XPS spectrum of SF@G-HF, indicating noncovalent binding between the Si and C components of SF@G-HF, as well as confirming the interfacial origin of these XPS signatures.

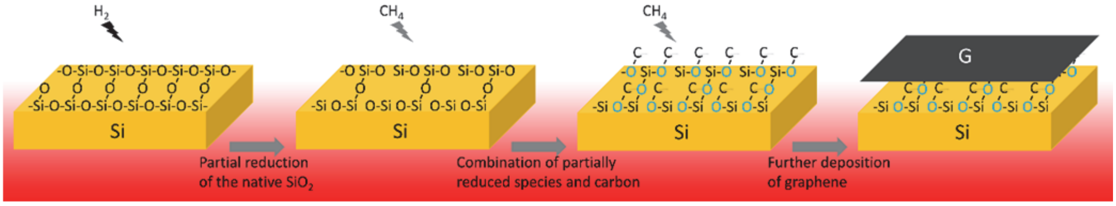

Supplementary Figure 9: Scheme illustrating the suggested formation route of Si–O–C bonds between the components of SF@G during the CVD process. Note that only one side of a silicon nanoplate is shown for clarity.

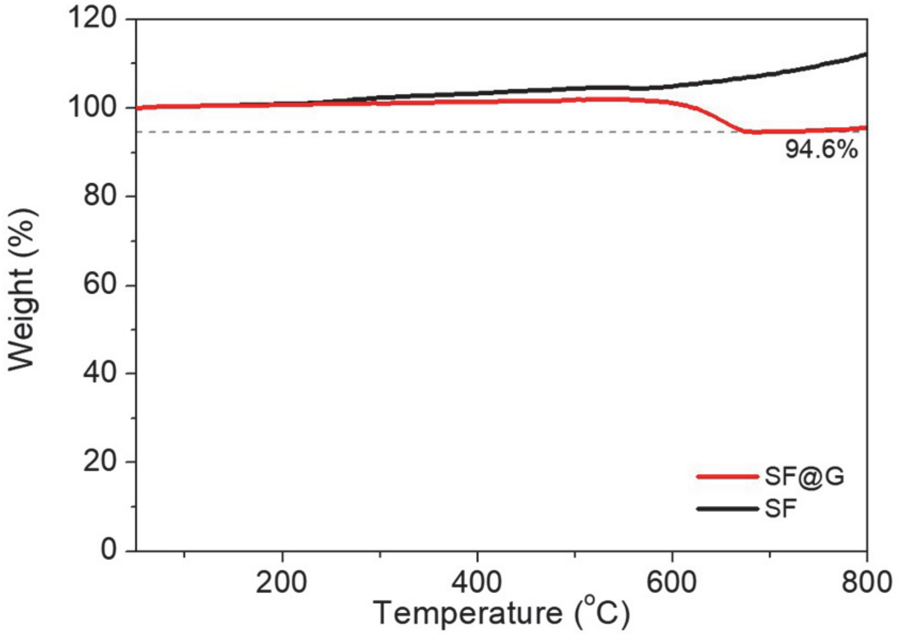

Supplementary Figure 10: TGA curves of SF@G and SF obtained under air atmosphere at a heating rate of 10 ºC min^{-1}. While the heat treatment in air resulted in an increase in the weight of SF due to the formation of silicon oxide, it induced the decomposition of G in SF@G and led to the weight decrease. Thus, the mass content of Si in SF@G could be calculated from the equation $W_G \times (1 - x) + W_{SF} \times x = W_{SF@G}$, where W_G, W_{SF} and $W_{SF@G}$ are the residual weight percentages of G, SF, and SF@G, respectively, and x is the mass content of Si in SF@G. Given that the G is completely decomposed and the W_G is close to zero, the x value could be estimated to be 87.6% based on the residual values at 700 ºC. A slight difference between the TGA and EDX results can stem from the thickness variation of Si nanoplates in SF@G.

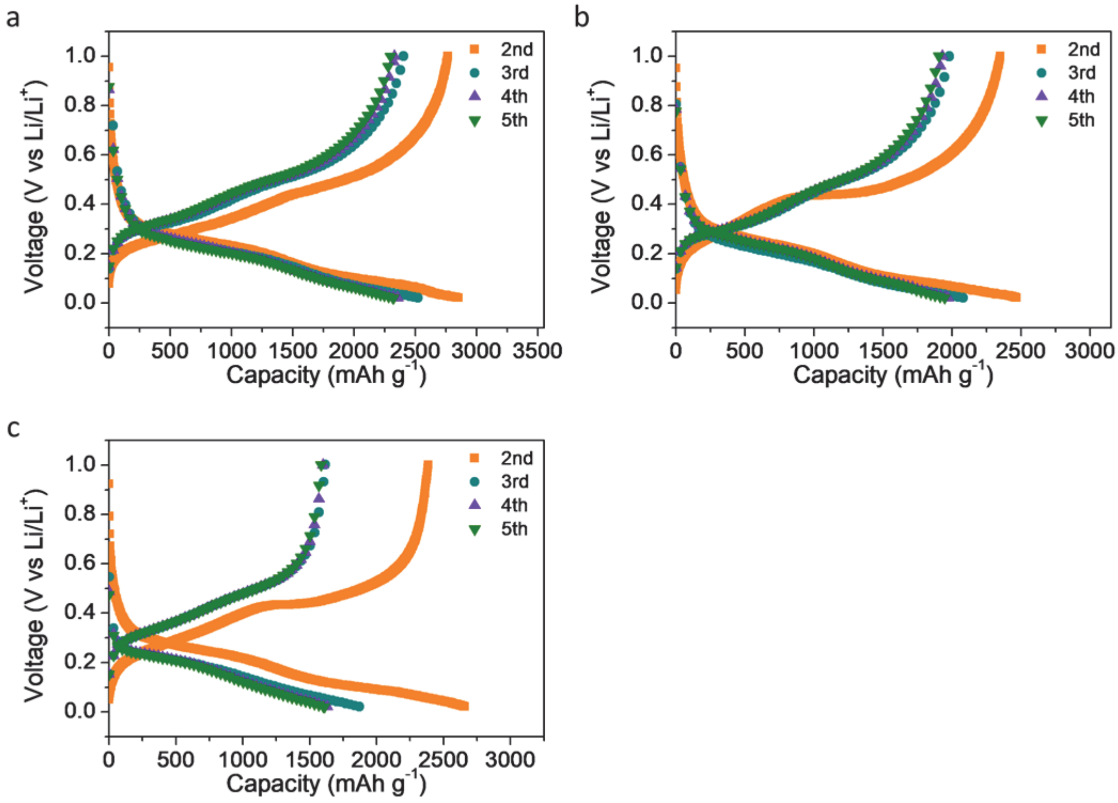

Supplementary Figure 11: Charge-discharge profiles of (**a**) SF@G, (**b**) SF@G-HF, and (**c**) SF for the 2nd, 3rd, 4th, and 5th cycles. The current rate is 200 mA g^{-1} for the 2nd cycle, and 2 A g^{-1} for the 3rd, 4th, and 5th cycles.

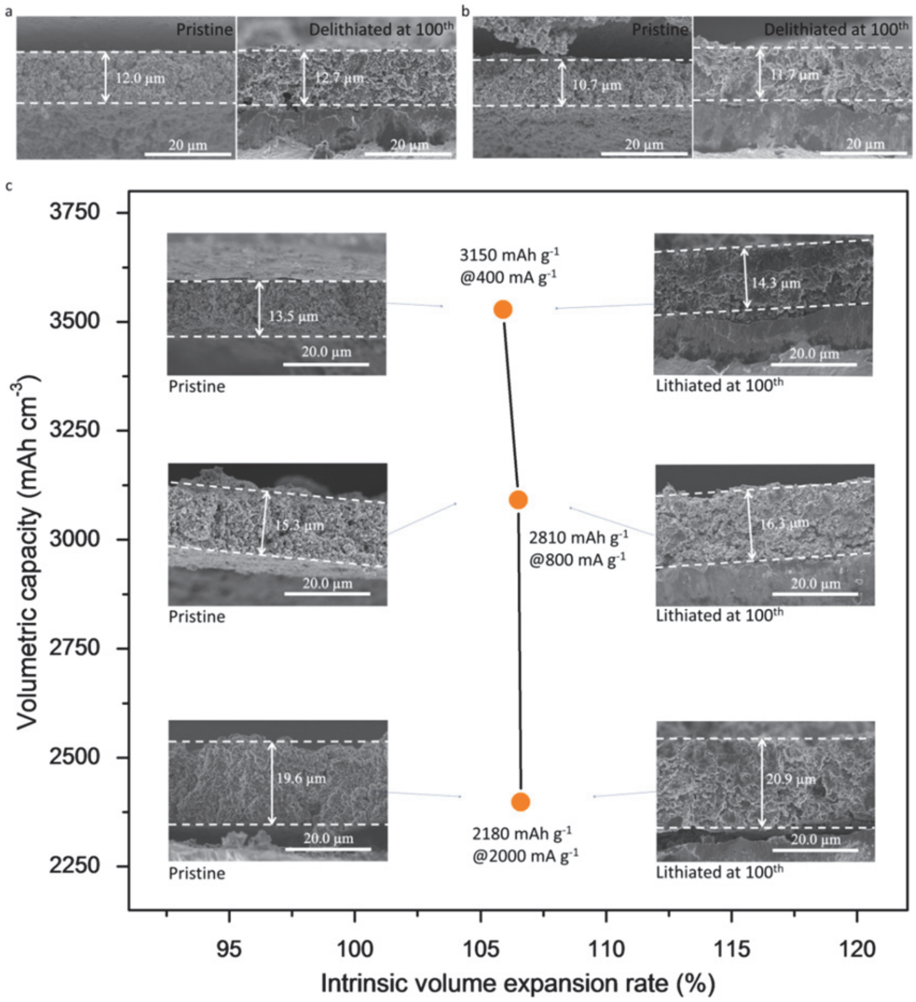

Supplementary Figure 12: **a**,**b**, SEM images of the cross section of a SF@G electrode (**a**) and (**b**) a SF@G-HF electrode in the annotated states. The SF@G and SF@G-HF electrodes show a thickness change of ~5.8 and ~9.3%, respectively. **c**, Plot of volumetric capacity *versus* Intrinsic volume expansion rate of SF@G electrodes with a slightly elevated areal capacity and packing density (~5 mAh cm^{-2} and ~1.1 g cm^{-3}, respectively), showing intrinsic volume expansion rates of 105.9~106.6%. The inset shows SEM images of the cross section of SF@ electrodes in the annotated states. *Note*: the intrinsic volume expansion rate is defined as an electrode thickness value in the full-lithiated state at 100th cycle divided by its pristine one.

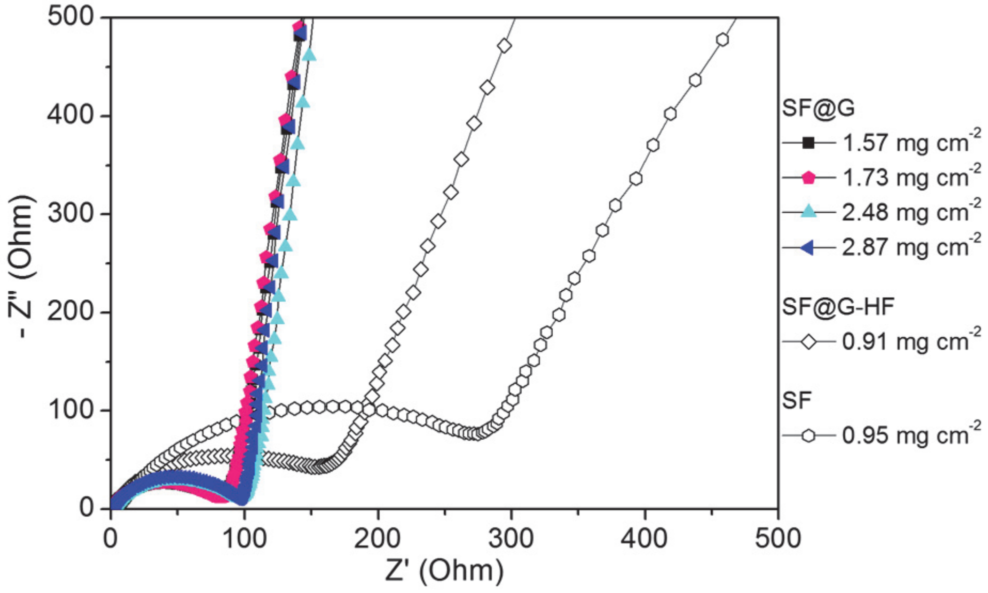

Supplementary Figure 13: Nyquist plots of SF@G, SF@G-HF, and SF at annotated loadings obtained from electrochemical impedance spectroscopy (EIS) measurements. All curves consist of a high frequency semicircle and a low frequency line. The inclined line corresponds to the lithium ion diffusion impedance, and the depressed semicircle at high frequency is mainly associated with interfacial charge transfer resistance. On the one hand, the comparison of the semicircle diameter clearly discloses that the charge transfer resistance of SF@G is significantly smaller than those for both SF@G-HF and SF. This scenario reflects fast electron transport in the SF@G, attributable to the two-dimensional covalent binding between silicon and carbon. On the other hand, the steeper slopes of the inclined line for SF@G compared with those for SF@G-HF and SF anodes are observed at the low-frequency region, depicting the efficiency of two-dimensional covalent binding in our design for fast diffusion and transport of lithium ions.

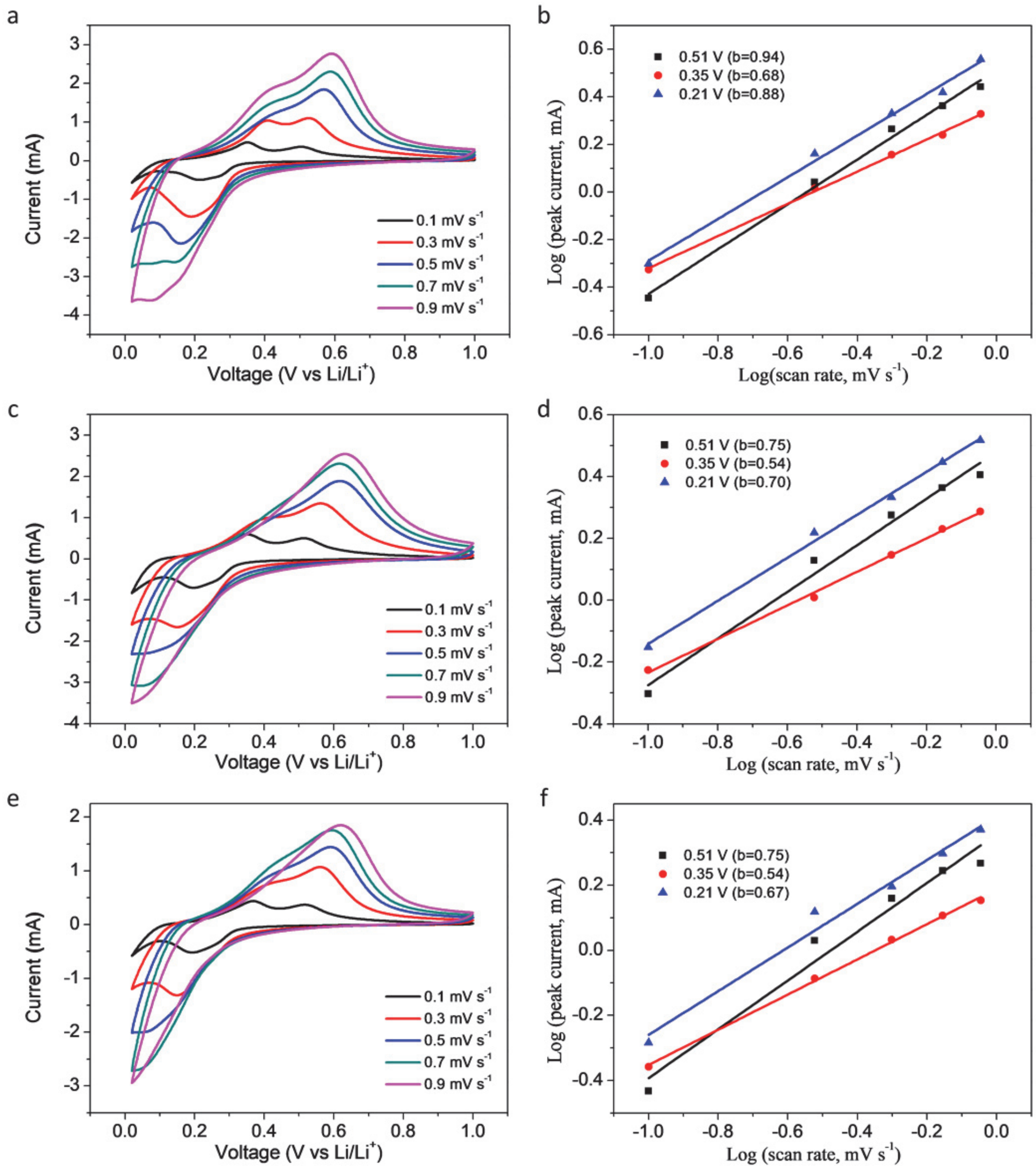

Supplementary Figure 14: **a**,**c**,**e**, Cyclic voltammograms (CV) at different scan rates. **b**,**d**,**f**, Plots of log (peak current) *versus* log (scan rate) for both cathodic (0.21 V) and anodic (0.35 V, 0.51 V) peaks. (**a**,**b**) SF@G. (**c**,**d**) SF@G-HF. (**e**,**f**) SF.

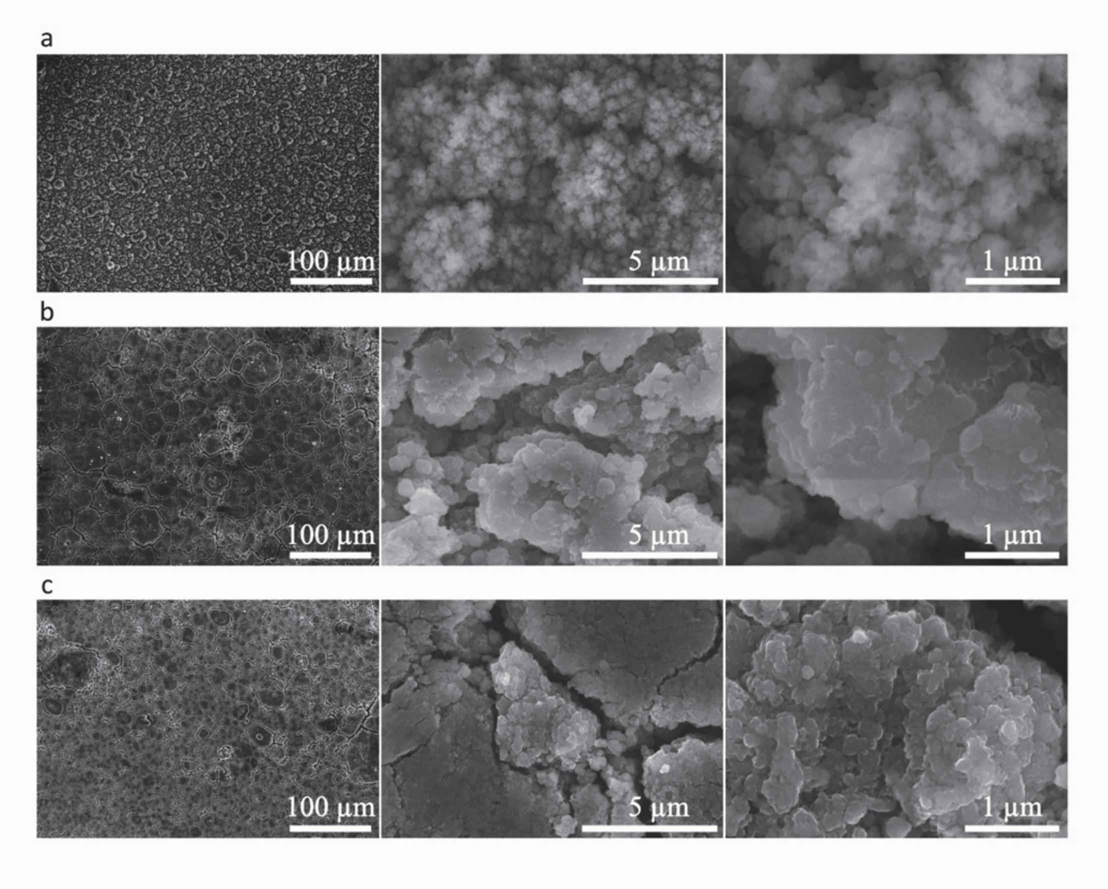

Supplementary Figure 15: SEM images of (**a**) SF@G, (**b**) SF@G-HF, and (**c**) SF after cycling for 100 times.

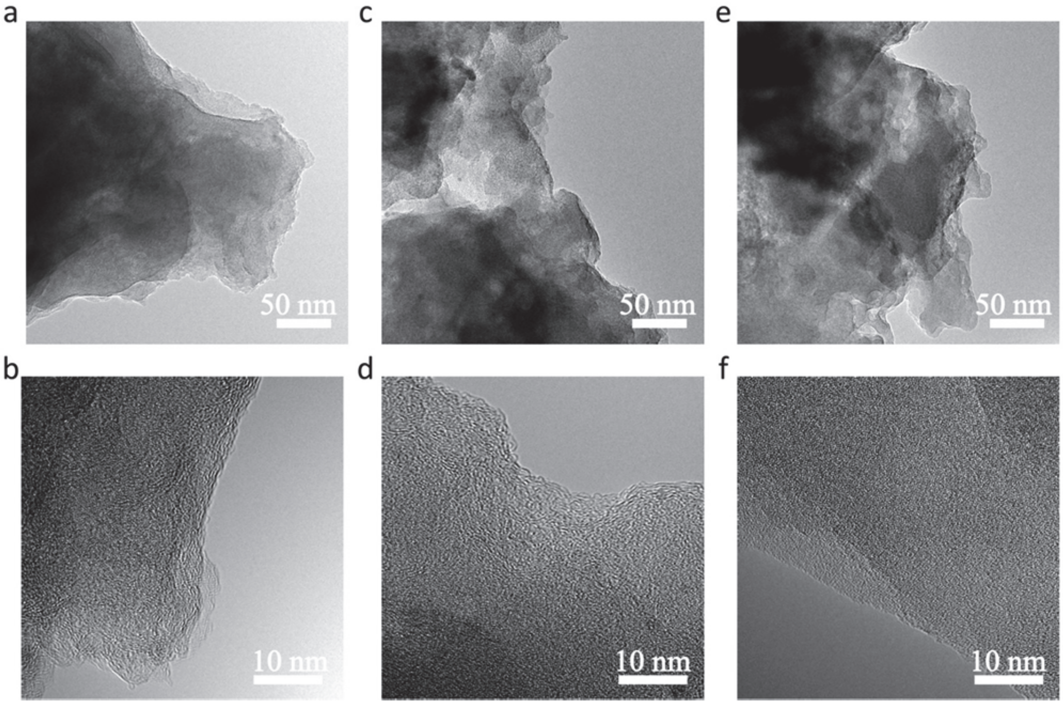

Supplementary Figure 16: TEM images of (a,b) SF@G, (c,d) SF@G-HF, and (e,f) SF after cycling for 100 times.

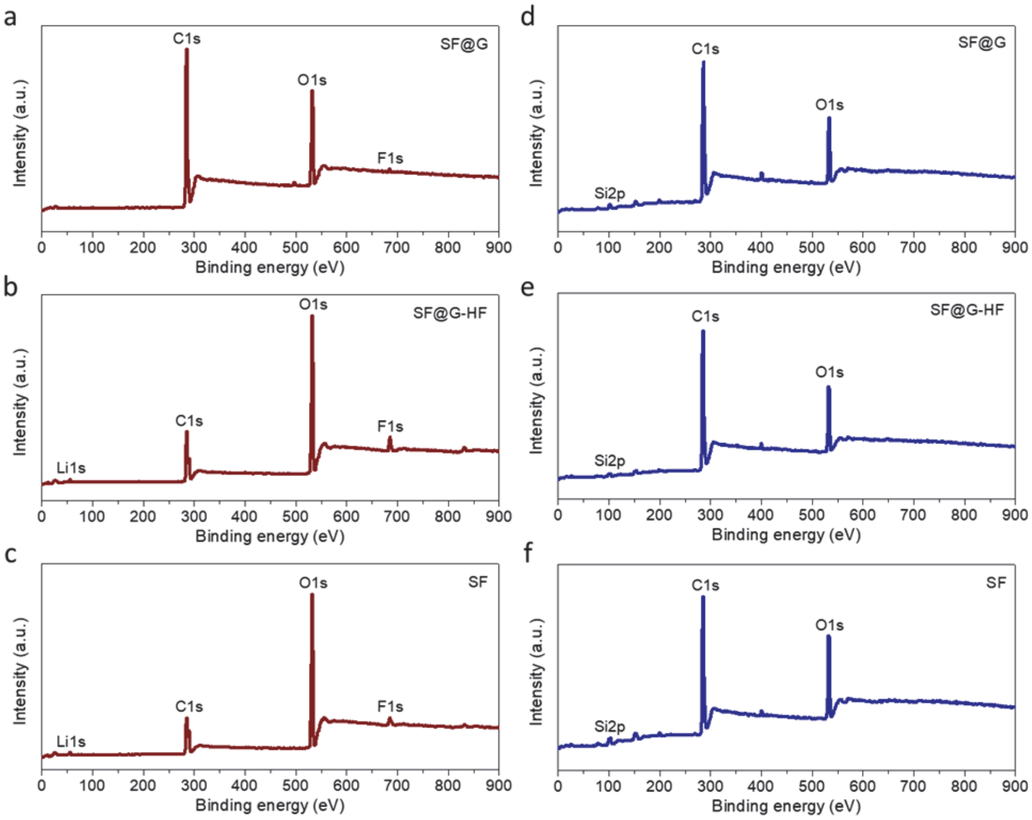

Supplementary Figure 17: XPS spectra of (**a**,**d**) cycled SF@G, (**b**,**e**) cycled SF@G-HF, and (**c**,**f**) cycled SF (**a**,**b**,**c**) before and (**d**,**e**,**f**) after HCl washing.

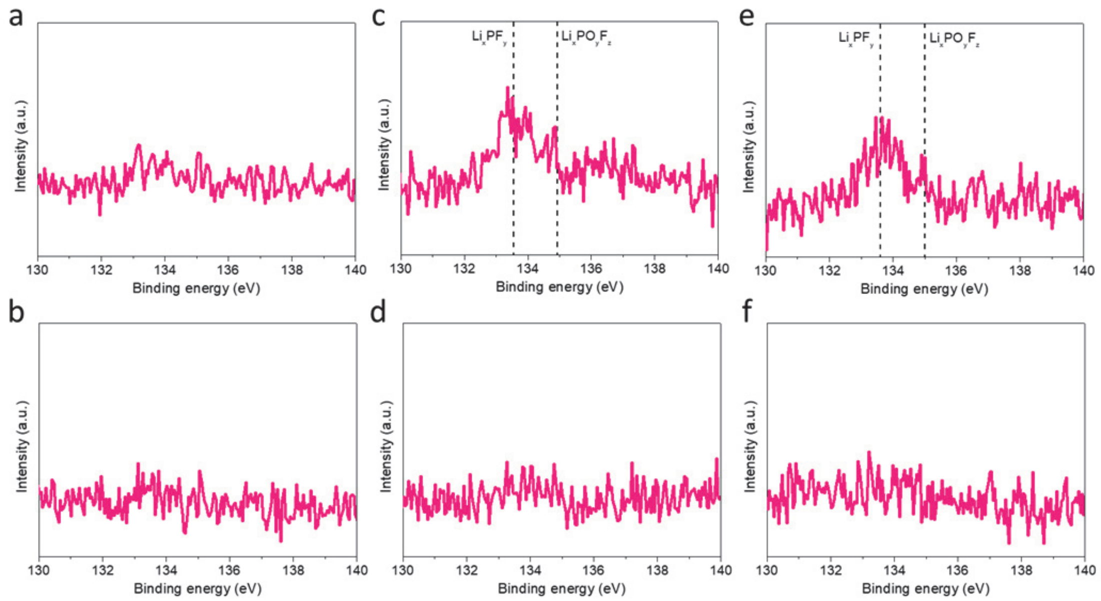

Supplementary Figure 18: P 2p XPS spectra of (**a**,**b**) cycled SF@G, (**c**,**d**) cycled SF@G-HF, and (**e**,**f**) cycled SF (**a**,**c**,**e**) before and (**b**,**d**,**f**) after HCl washing. The peaks at 133.5 and 134.9 eV in (**c**) and (**e**) can be assigned to Li_xPF_y and $Li_xPO_yF_z$, respectively, in line with their F 1s spectra, showing the occurrence of decomposed $LiPF_6$ species in the cases of SF@G-HF and SF.

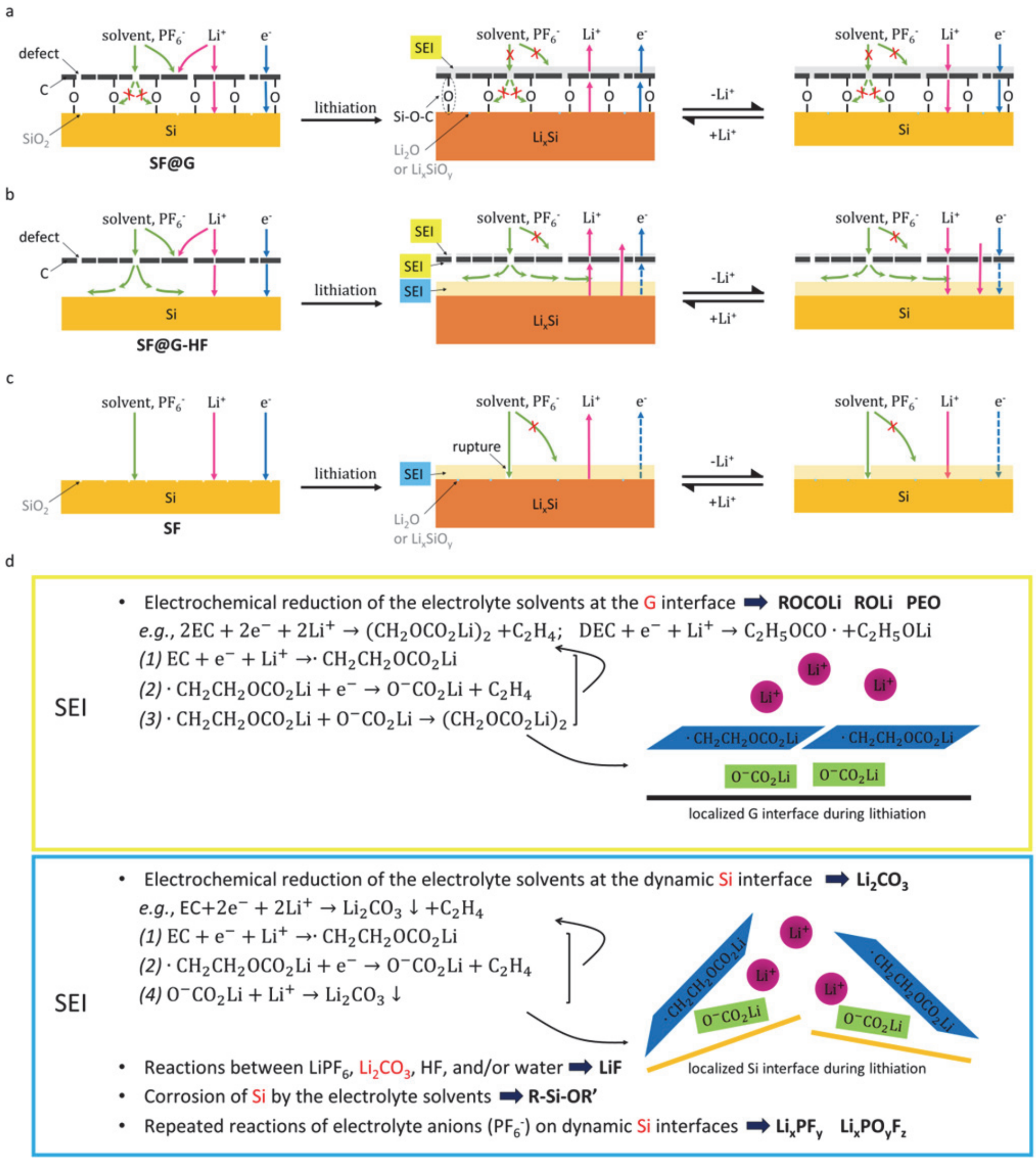

Supplementary Figure 19: a-c, Scheme illustrating the SEI formation on (**a**) SF@G, (**b**) SF@G-HF, and (**c**) SF. In all three cases, the transport pathways for electrons (e^-), lithium ions (Li^+), and others (solvent, PF_6^-) are shown as well. Note that the solid and dashed blue arrows represent fast and slow electron transport, respectively. **d**, Proposed origin of the interface-dependent SEI majority based upon typical reactions of the involved solvents (*e.g.*, EC).

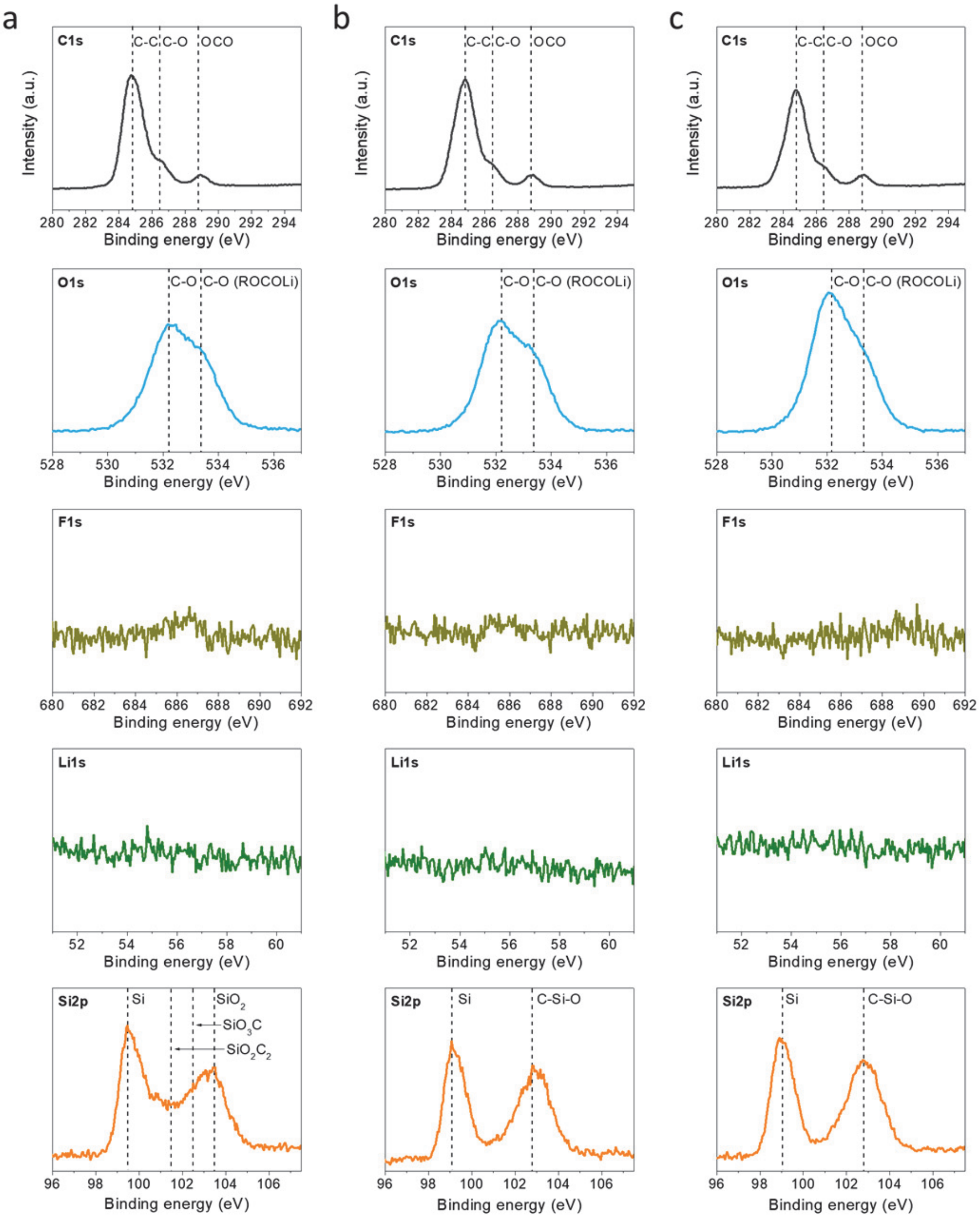

Supplementary Figure 20: C 1s, O 1s, F 1s, Li 1s, and Si 2p XPS spectra of (**a**) cycled SF@G, (**b**) cycled SF@G-HF, and (**c**) cycled SF after HCl washing.

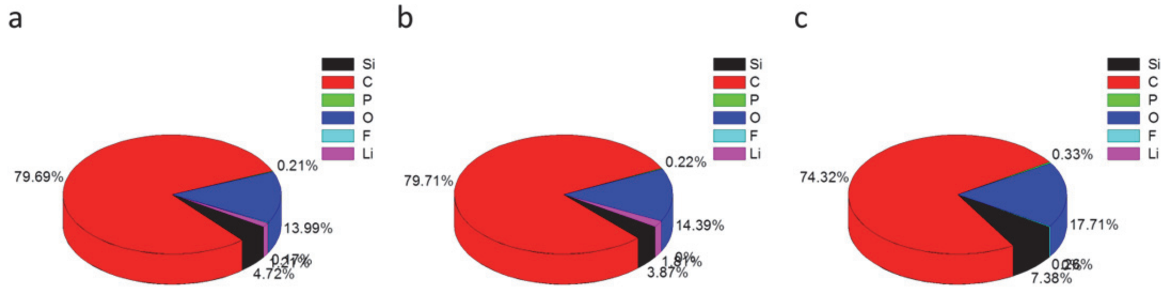

Supplementary Figure 21: Interfacial element concentrations of (**a**) cycled SF@G, (**b**) cycled SF@G-HF, and (**c**) cycled SF after HCl washing.

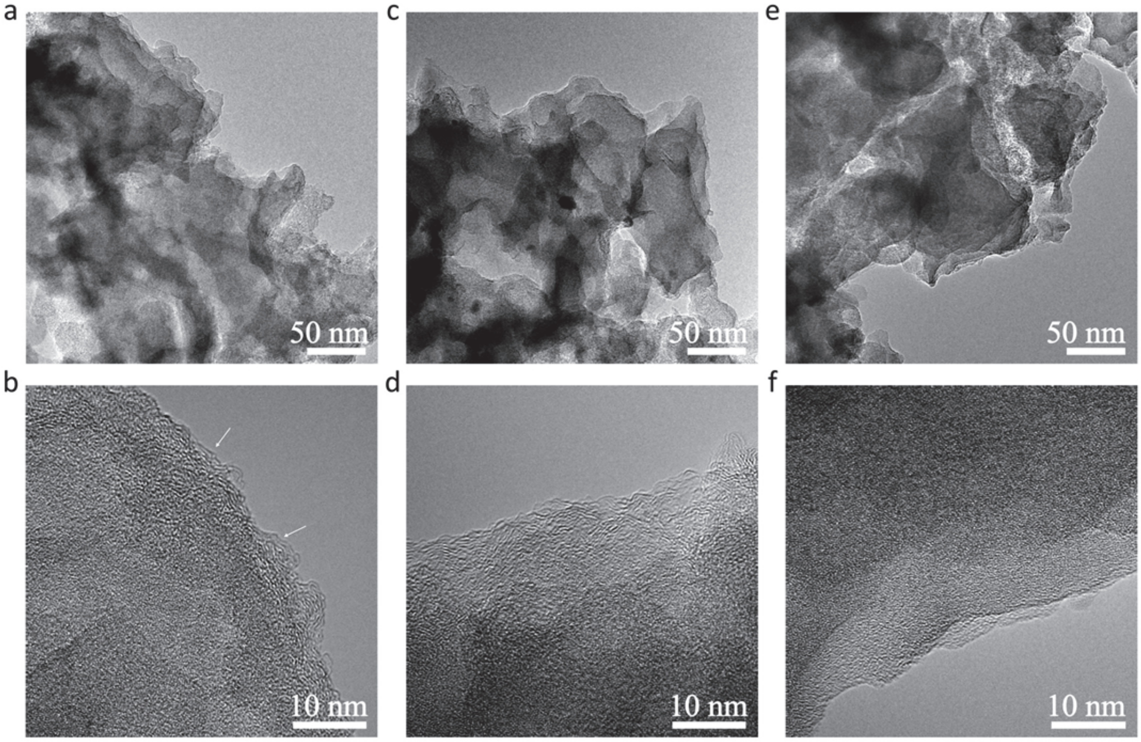

Supplementary Figure 22: TEM images of (**a**,**b**) cycled SF@G, (**c**,**d**) cycled SF@G-HF, and (**e**,**f**) cycled SF after HCl washing.

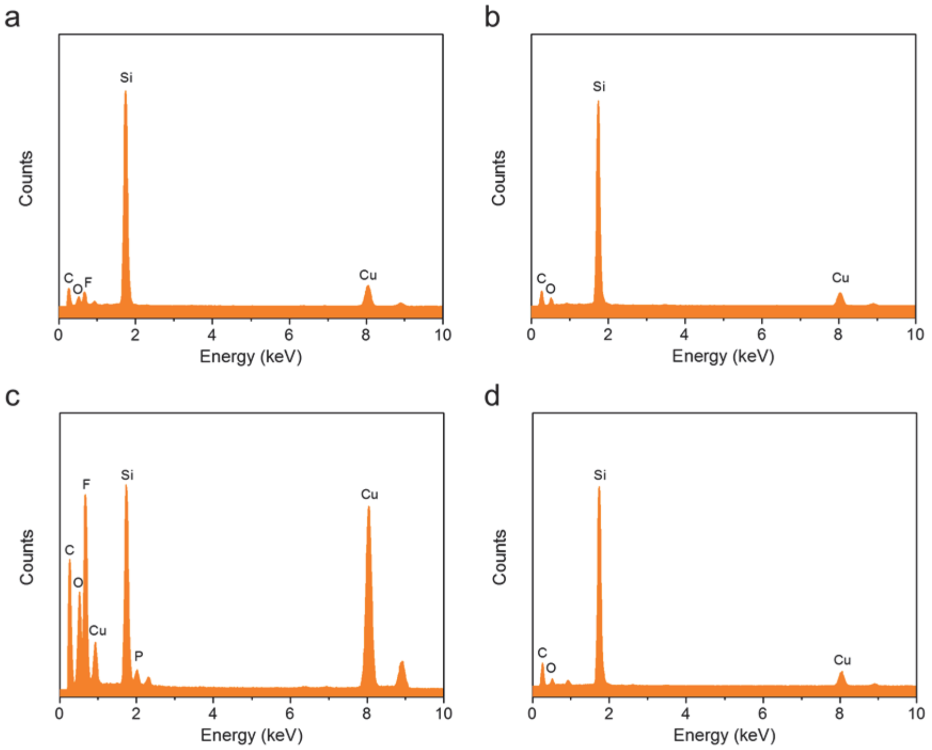

e

	Cycled SF@G w/o & w/ HCl washing		Cycled SF@G-HF w/o & w/ HCl washing	
	Weight % (w/o)	Weight % (w/)	Weight % (w/o)	Weight % (w/)
C(K)	15.64	15.04	35.24	20.63
O(K)	5.06	4.29	15.66	4.71
Si(K)	72.35	79.82	20.9	73.17
F(K)	6.57	0.58	26.49	1.13
P(K)	0.35	0.25	1.69	0.33

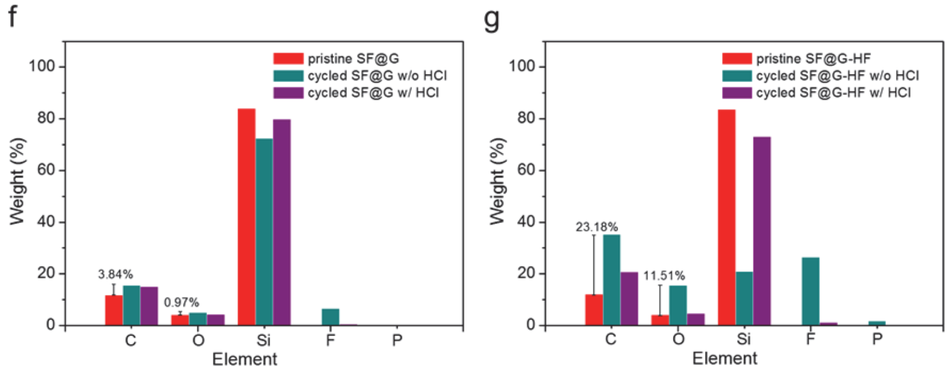

Supplementary Figure 23: **a**,**b**, EDX patterns of cycled SF@G (**a**) w/o and (**b**) w/ HCl washing. **c**,**d**, EDX patterns of cycled SF@G-HF (**c**) w/o and (**d**) w/ HCl washing. **e**, Weight ratios of cycled SF@G and SF@G-HF w/o and w/ HCl washing. **f**,**g**, Comparison of weight ratios between pristine and cycled (**f**) SF@G and (**g**) SF@G-HF w/o and w/ HCl washing.

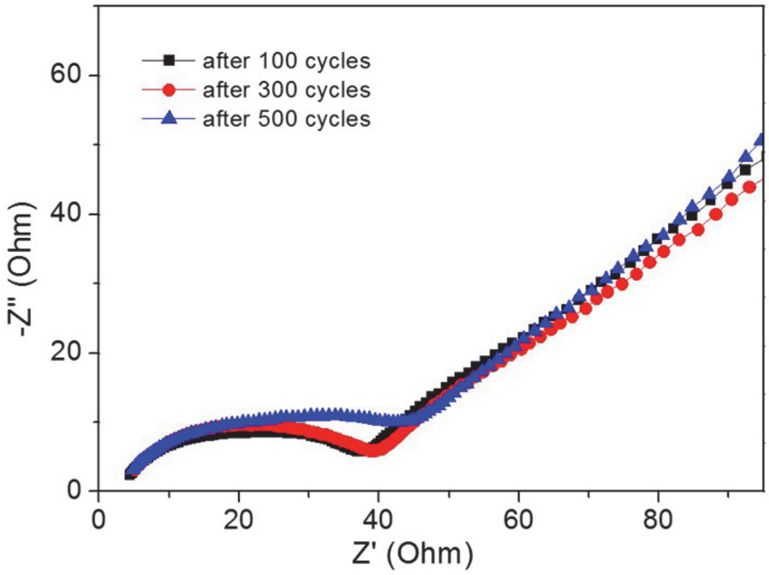

Supplementary Figure 24: Nyquist plots of SF@G after 100, 300, and 500 cycles.

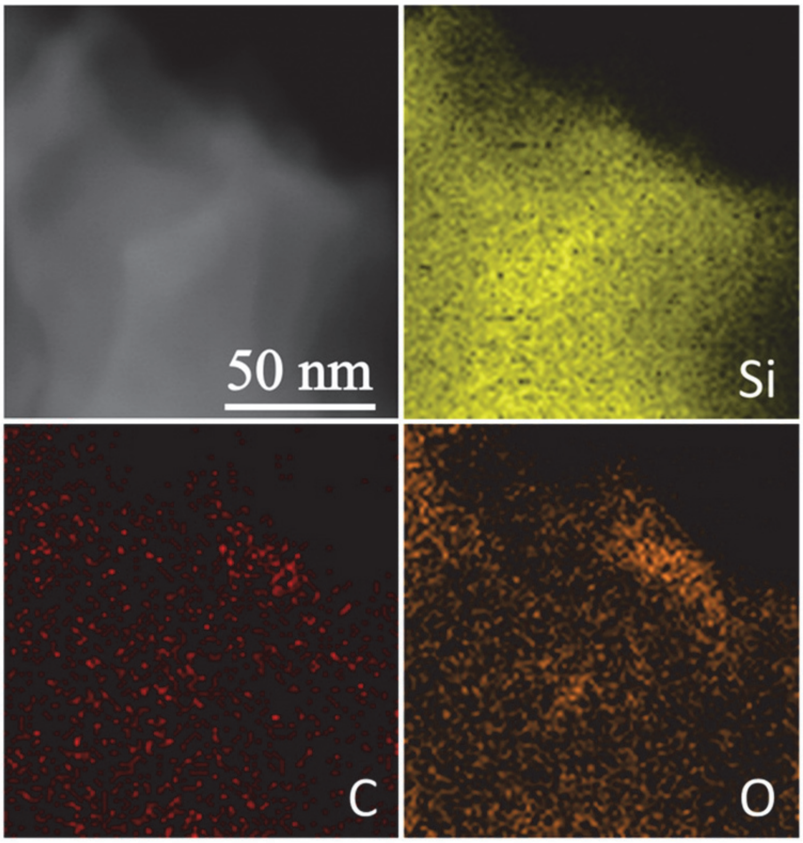

Supplementary Figure 25: STEM and elemental mapping images of cycled SF@G after HCl washing.

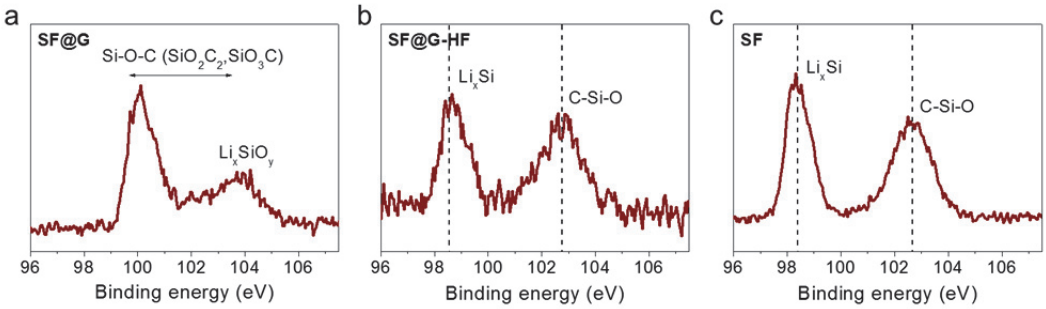

Supplementary Figure 26: Si 2p XPS spectra of (**a**) lithiated SF@G, (**b**) lithiated SF@G-HF, and (**c**) lithiated SF. Note that the peak shift of Si-O-C species in the case of lithiated SF@G relative to their original one (Fig. 2h in the main text) reflects the interaction with lithium during discharging.

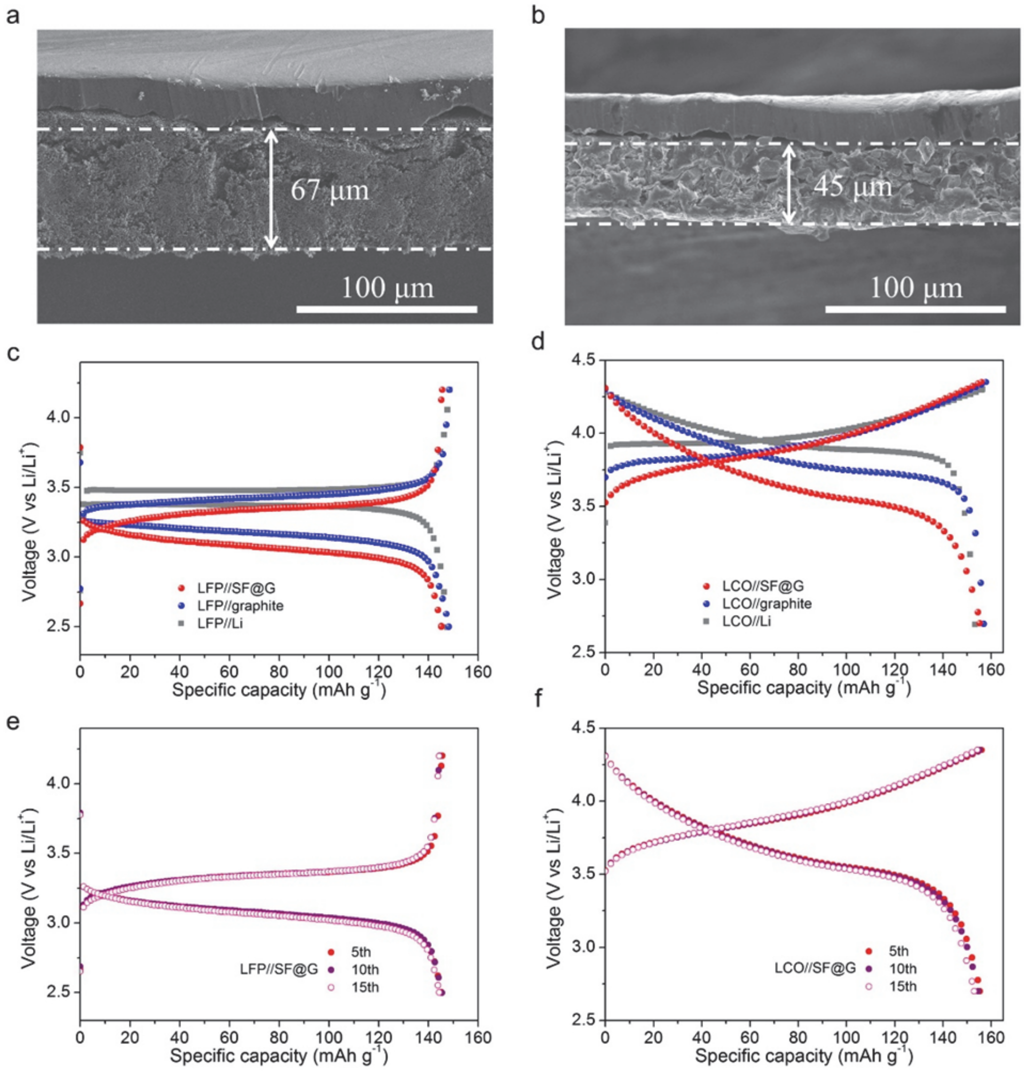

Supplementary Figure 27: **a**,**b**, SEM images of the cross section of (**a**) a lithium iron phosphate (LFP) cathode and (**b**) a lithium cobalt oxide (LCO) cathode. **c**, Typical voltage profiles of LFP//SF full cell, LFP//graphite full cell, and LFP//Li half-cell. **d**, Typical voltage profiles of LCO//SF full cell, LCO//graphite full cell, and LCO//Li half-cell. **e**,**f**, Voltage profiles of the full cells of (**e**) LFP//SF@G and (**f**) LCO//SF@G. *Note*: All the cathodes and full cells were fabricated following the similar process as described in the main text.

**LFP, LCO cathode-involved full cells**				
**Electrode configuration**				
	SF@G	Graphite	LFP	LCO
**Electrode area (cm^2)**	1.13	1.13	1.13	1.13
**Mass loading ($mg\ cm^{-2}$)**	1.34	7.14	16.96	15.9
**Electrode thickness (μm)** [a]	12	48	67	45
**Full cell characteristic**				
	LFP// SF@G	LFP// Graphite	LCO// SF@G	LCO// graphite
**Operation voltage (V)** [b]	3.1	3.2	3.7	3.83
**Specific energy (Wh/kg)** [c]	**433**	**338**	**548**	**423**
**Specific energy $(\text{Wh/kg})_{cell}$** [d]	**255**	**202**	**323**	**253**
**Energy density (Wh/L)** [e]	**802**	**567**	**1326**	**838**

**NCA cathode-involved full cells (5 mAh/cm^2)**		
**Electrode and cell configuration**		
	SF@G	NCA
**Specific capacity ($mAh\ g^{-1}$)**	2000	198
**Mass loading ($mg\ cm^{-2}$)**	2.73	26.6
**Electrode press density ($g\ cm^{-3}$)**	0.94	3.5
**Active material weight ratio (%)**	96	96
**Electrode thickness (μm)**	29	75.9
**Current collector thickness (μm)**	8 (Cu)	12 (Al)
**N/P ratio**	1.05	
**Separator thickness (μm)**	9 (50% porosity of PE with two sides of 1.5 μm MgO and PVDF coating)	
**Electrolyte amount ($cm^3\ Ah^{-1}$)**	1.8	
**Full cell characteristic**		
	NCA//SF@G	
**Operation voltage (V)**	3.7	
**Specific energy (Wh/kg)** [c]	**667**	
**Energy density (Wh/L)** [e]	**1790**	
**Specific energy $(\text{Wh/kg})_{cell}$**	**394** [f]	**367** [g]
**Energy density $(\text{Wh/L})_{cell}$**	**770** [f]	**850** [h]

Supplementary Table 1: Electrode configurations and estimated[1] specific energy and energy density levels of full cells.

Supplementary Note 1:

Considering the same cathode (LFP or LCO), the comparison of specific energy and energy density at the material scale[2] and the cell scale between SF@G-based full cells and graphite-based ones reveals the successful design of SF@G, the projected energy level in a current state-of-the-art NCA-involved cell configuration further points to the viability and potential of SF@G. *Note*: a. The electrode thickness for LFP and LCO is acquired by SEM observation as shown in Supplementary Fig. 27. b. The operation voltage is obtained based upon the voltage profiles of full cells as exhibited in Supplementary Fig. 27. c. The specific energy is in terms of both the anode and cathode. d. The estimated specific energy in terms of the whole cell based on previously reported material-to-cell factors of 59~61% (which can be further increased depending on cell formats)[3,4], pointing to 26 and 28% increase for LFP//SF@G and LCO//SF@G when compared to their counterparts, respectively. e. The energy density is on the basis of the total volume of the anode and the cathode; the volume variation (5.8~6.6%) of SF@G upon cycling can incur a slight decrease in the estimated values. f. The estimated specific energy and energy density in terms of the whole cell based on a typical material-to-cell ratio for an 18650-type cell (59 and 43%, respectively)[3]. g. The evaluated specific energy in terms of the whole cell based on the displayed cell configuration and components, indicating an increase of up to 41% when compared to that of the current state-of-the-art LCO//graphite (w/ 3wt% SiO_x) commercial full cell

(~260 Wh/kg at 3500 mAh cell level). h. The calculated energy density in terms of the whole cell based on the displayed cell configuration and components (w/o considering cell housing, poles, and tabs).

Supplementary Note 2:

It is known that the voltammetric response of active electrode materials at various sweep rates can be summarized according to the equation $\mathrm{i} = \mathrm{a} \times v^{b}$, in which the current ($\mathrm{i}$) obeys a power law relationship with the sweep rate (v), as well as a and b are adjustable values. It has been well established in previous investigations of other energy storage electrode materials that there are two well-defined conditions: b = 0.5 and b = 1.0[5-7]. For b = 0.5, the current in a cyclic voltammetry (CV) experiments varies with the square root of the sweep rate, which reveals a typical battery behavior controlled by semi-infinite linear diffusion. The other defined condition of b = 1 implies that the current is proportional to the sweep rate, which reveals a surface-controlled charge storage process. Based on the plots of log (peak current) *versus* log (scan rate) for both cathodic and anodic peaks (Supplementary Fig. 14), the b values were obtained and summarized in Fig. 4f. Notably, all the b values relating to anodic and cathodic peak currents of SF@G are significantly higher than those for SF@G-HF and SF, confirming much improved lithium storage kinetics in SF@G mostly enabled by the involved skin-like binding. It should be noted as well that the similar b values of SF@G-HF and SF suggest the comparable interfacial contact between the silicon and adjacent electrically conductive

media in both cases upon cycling.

Supplementary Note 3:

As shown in Supplementary Fig. 19, it is proposed that the observed distinct alteration in SEI components of SF@G and control samples (SF@G-HF, SF) may mainly result from different reduction reactions of the used solvents (e.g., EC, DEC). Specifically, as for EC, its reduction is generally considered via ring opening as shown in reaction (1), followed by further electron transfer reactions, for example, reaction (2). Due to the covalent encapsulation in SF@G fundamentally blocking direct contact of Si with the electrolyte, the product of reaction (1) forms and accumulates near the relatively stable G interface. Thereby, the product of the successive reaction (2) can readily react with the already-existing product of reaction (1) to produce semi-organic carbonates, for example, by reaction (3) before interacting with lithium ions. By a further one-electron reduction, these carbonate species can be converted into either lithium alkoxides (ROLi) or radical esters that can coordinate with lithium to form lithium carboxylates (ROCOLi). In contrast, in the case of SF@G-HF and SF with silicon directly interfacing with the electrolyte, the product of reaction (1) will be randomly scattered away from the localized dynamic Si interface during lithiation. Thereby, the neighboring lithium ions have more chances to combine the product of successive reaction (2), resulting in the formation of Li_2CO_3 via reaction (4). Furthermore, the direct contact of silicon with the electrolyte in SF@G-HF and SF also leads to the

propagation of other inorganic SEI components such as partially decomposed $LiPF_6$ constituents (Li_xPF_y and $Li_xPO_yF_z$) by repeated reactions of electrolyte anions (PF_6^-) on dynamic Si interfaces, and siloxane structures (R–Si–OR') upon severe corrosion of Si by the electrolyte solvents.

Supplementary Note 4:

As shown in Fig. 5d-f, in the C 1s spectra of cycled SF@G-HF and SF an additional distinguishable shoulder corresponding to C–Si bonds is observed at 283.0 eV. This finding is in line with the result obtained from their Si 2p spectra, where a distinct peak located at 102.6 eV corresponds to C–Si–O bonds, assignable to siloxane structures (labeled as R–Si–OR'). The siloxane species may be soluble in the electrolyte and diffuse throughout the SEI layer; their presence manifests the severe corrosion of Si by the electrolyte solvents in both cases. In sharp contrast, the corresponding peak is absent at 283.0 eV in the C 1s spectrum of SF@G, suggesting that the direct contact and undesirable corrosion of Si with the electrolyte solvents are effectively inhibited by the encapsulation concept. This protection is also confirmed by the absence of any discernible peak in the Si 2p spectrum of cycled SF@G. Furthermore, in the F 1s spectra for all three cycled samples, there exists a major peak at 684.7 eV, assignable to LiF derived from various mechanisms including reactions between $LiPF_6$, Li_2CO_3, HF, and/or water. Remarkably enough, this signal is very weak in the case of SF@G when being compared with SF@G-HF and SF. The similar phenomenon is also observed for the LiF peak

at 55.4 eV in their Li 1s spectra, reflecting a much smaller amount of LiF present in the cycled SF@G relative to those in SF@G-HF and SF. A large amount of LiF in SF@G-HF and SF is proposed to be related to the presence of Li_2CO_3 in their SEI layers considering the same cell formula and assembly condition. Besides, the F 1s spectra of both SF@G-HF and SF show a significant peak at 687.9 eV, which can be ascribed to partially decomposed $LiPF_6$ constituents labelled as Li_xPF_y and $Li_xPO_yF_z$, consistent with their P 2p spectra (Supplementary Fig. 18). The presence of these phosphorus species is also depicted as a shoulder at 56.5 eV in the Li 1s spectra of SF@G-HF and SF. The occurrence of decomposed $LiPF_6$ is primarily associated with repeated reactions of electrolyte anions (PF_6^-) on dynamic Si interfaces in both SF@G-HF and SF. By comparison, there is no detectable signal at the same binding energy in the F 1s, Li 1s, and P 2p spectra of SF@G, indicating effective suppression of the PF_6^--involved reactions on the interface of skin-like bound structure.

Supplementary Note 5:

As shown in Supplementary Fig. 23, the relative value of incremental weight ratios of C to O in cycled SF@G and SF@G-HF relative to their pristine counterparts is 4 and 2, respectively. This discrepancy discloses the average stoichiometry of distinct interfacial SEI species in both cases as revealed by XPS (Fig. 5d, 5e, and 5g) in view of the identical silicon and carbon components in their pristine counterparts. Furthermore, the significant change

in weight ratios of C and O in cycled SF@G-HF upon HCl washing can be associated with its inorganic-dominated interface. In stark contrast, the change in weight ratios of C and O in cycled SF@G upon HCl washing is indistinguishable which can be attributed to its organic-dominated interface and stable nature. In addition, a slight increase in weight ratios of C and O in cycled SF@G relative to its pristine one suggests the presence of a low amount of SEI components, in good agreement with the SEM observation (Fig. 5a). By comparison, the observed substantial increase in weight ratios of C and O in cycled SF@G-HF indicates the introduction of a large amount of SEI species as exhibited in Fig. 5b.

Supplementary Note 6:

The preservation of covalent binding between Si nanoplates and G nanosheets in SF@G upon charging and discharging may be attributed to a reversible interaction of the involved Si–O–C species (e.g., SiO_2C_2 and SiO_3C) with lithium, resembling the reversible insertion described previously for Si–O–C bulk materials[8]. This scenario is also verified by the XPS results of lithiated SF@G, which indicates the presence of Si–O–C species interacting with lithium (Supplementary Fig. 26)[8]. Furthermore, upon interacting with lithium ions, the remaining SiO_2 in each SF@G nanoplate, if there is any, can be converted into Li_2O (or Li_xSiO_y (e.g., $Li_2Si_2O_5$, Li_4SiO_4)) and Si by reactions of $\mathrm{SiO_2 + 4Li^+ + 4e^- \rightarrow 2Li_2O + Si}$, $\mathrm{5SiO_2 + 4Li^+ + 4e^- \rightarrow 2Li_2Si_2O_5 + Si}$, and/or $\mathrm{2SiO_2 + 4Li^+ + 4e^- \rightarrow Li_4SiO_4 + Si}$. While the Li_2O (or Li_xSiO_y) is randomly

distributed between already-existing Si–O–C species (or domains) along the silicon-carbon interface as schematically illustrated in Supplementary Fig. 19, the thus-resulted Si can be further contacted or fused with the silicon bulk of the nanoplate during its reversible lithiation and delithiation via $\mathrm{Si} + \mathrm{yLi}^{+} + \mathrm{ye}^{-} \leftrightarrow \mathrm{Li_{y}Si}$. It should be noted that there is no distinguishable Li_2O peak (~53 eV in the Li 1s spectrum) and Li_xSiO_y peak (~530 eV in the O 1s spectrum) for cycled SF@G (Fig. 5d), which rather implies their insignificant amount.

Supplementary References

1. Zhang, X. *et al*. Silicene flowers: A dual stabilized silicon building block for high-performance lithium battery anodes. *ACS Nano* **11**, 7476-7484 (2017).

2. Ko M. *et al*. Scalable synthesis of silicon-nanolayer-embedded graphite for high-energy lithium-ion batteries. *Nat. Energy* **1**, 16113 (2016).

3. Betz J. *et al*. Theoretical versus practical energy: a plea for more transparency in the energy calculation of different rechargeable battery systems. *Adv. Energy Mater.* **9**, 1803170 (2019).

4. Placke T. *et al*. Lithium ion, lithium metal, and alternative rechargeable battery technologies: the odyssey for high energy density. *J. Solid-State Electrochem.* **21**, 1939-1964 (2017).

5. Augustyn, V. *et al*. High-rate electrochemical energy storage through Li^+ intercalation pseudocapacitance. *Nat. Mater.* **12**, 518-522 (2013).

6. Lindström, H. *et al*. Li^+ ion insertion in TiO_2 (anatase). 2. Voltammetry on nanoporous films. *J. Phys. Chem. B* **101**, 7717-7722 (1997).

7. Wang, B. *et al*. Approaching the downsizing limit of silicon for surface-controlled lithium storage. *Adv. Mater.* **27**, 1526-1532 (2015).

8. Liu, X. *et al*. Mechanism of lithium storage in Si–O–C composite anodes. *J. Power Sources* **196**, 10667-10672 (2011).
