# Supplementary material for: Stable high-capacity and high-rate silicon-based lithium battery anodes upon two-dimensional covalent encapsulation
Source: Nat Commun. 2020 Jul 31;11:3826. doi: 10.1038/s41467-020-17686-4 (PMC7395733; doi:10.1038/s41467-020-17686-4)
Supplement: Supplementary file 2 — Peer Review File [file 41467_2020_17686_MOESM2_ESM.pdf]

Reviewers' comments:

Reviewer #1 (Remarks to the Author):

The authors synthesized nano-engineered Si-C structure, interface of which is encapsulated by covalent bonding, resulting in great improvement of both capacity retention and Coulombic efficiency of Si-anode based full cells. Various examination viewpoints are considered and systematically organized. That said, there are few things that needs to be clarified prior to me commening on whether or not this work deserves the publicaiton in Nature communicaitons. Thsese include,

1. The positive effect of covalent interface is varified comparing SF, SF@G, and SF@G-HF. That said, as far as I notice, there are a few works that fundamentally deliver the covalent concept in previous arts such as

Hasan, et. al
https://www.nature.com/articles/ncomms9597
Osaka et. al
https://pubs.rsc.org/en/content/articlelanding/2014/ta/c3ta13080k#!divAbstract

These works show the covalent effect is particulaly exhibited only when the base Si structure is quite small feature size, such as <10nm, i.e. cell performance improvement by covalent encapsulation is not global but synergized when combined with ultra-thin Si. Also the work,

Ogata et. al
https://www.nature.com/articles/s41467-018-02824-w

shows in-situ stabilization of reversibility, showing the composite turns into covalent encapsulated sub 3nm Si system. The author needs to clarify the intrinsic novelty of the work compared to the previous covalent works.

2. There are hundreds of nano-engineered Si-C composites published for the last 10 years. However, unfortunately, most of them neglect density (g/cc) and volumetric/gravimetric energy density (Wh/L or Wh/kg) after volumet expansion when combined with cathode. Some S-C composites only shows 0.5-1.0 g/cc despite its high capacity such as 2000mAh/g, ending up with 1000-2000mAh/cc. Further, volume expansion needs to be considered. There is an approximately linear correlationship between mAh/cc and volume expansion rate, thus the mAh/cc needs to be devided by volume expansion rate, e.g. if 180%, 2000mAh/cc devided by 1.8 = 1100mAh/cc effectively. This effective number needs to be taken into account into calculation of Wh/L and Wh/kg. Further, with increase of Si % in the anode, discharge voltage drop gets significant due to nature of Cap-Voltage profile of Si. This can reduce energy density indeed. In the actual commercialization stage, people always realize that after volume expansion and voltage drop in use of higher concentration Si there is not so much energy density gain in view of whole device. So the author needs to clarify advantage of their work in this view point.

Reviewer #2 (Remarks to the Author):

The authors synthesized silicon-graphene hybrids in the skin-like form where Si and C are covalently bonded and used the hybrids as anode for lithium batteries. They found that the covalent binding makes electrode material superior compared to other Si-C hybrid forms.

A central, outstanding issue for silicon anodes is its large volume change upon charge and discharge. The authors acknowledge that. In literature, as long as the silicon loading is sufficiently large to make

the volumetric capacity sufficiently high, this mechanics issue arises. However, throughout the manuscript, the authors did not mention about this, despite that they acknowledge volume change is a serious issue in previous studies. It is important to report to the readers and the community how this issue is resolved (or preserved). If this issue remains, i.e., if volume change exists for the Si-C hybrid material, the authors need to address why the volume change does not (or does) influence the performance of the material.

I would be supportive to this manuscript to be accepted by Nature Comm, provided that the above issue is carefully addressed.

Reviewer #3 (Remarks to the Author):

The paper demonstrates a nice energy density and electrochemical stability. However, to verify the concept of the atuhros, further investigations are needed. Otherwise, the results are one of good results in the field of silicon based anodes for LIBs. So far, the paper does not reach the level of Nature Commun..

In the concept of the authors, the graphene layer is fixed by silicon oxide and carbon bonding. However, when lithiated, SiO_x is reacted into Li_2O and Li_xSi. The authors need to verify how the silicon layer and the graphene layer are bonded after lithiation.
Acutely, The data of SF@G is superior to the data of SF@G-HF. However, this is not direct evidence to verify the concept of the authors.

The authors explained that the electrolyte penetrates through pinholes and/or defects of graphene without direct contact of Si with the electrolyte. If the ionic pathway is limited like this, the authors need to verify how such high current operation (20 A/g) was achieved.

These are critical points. And small questions are followings.

The authors had better to explain what b value is.

The magnitude of Fig 5a-c is not enough to tell us the flower-like structure.

The SEIs on SF@G and "SF@G-HF and SF" are different. I understand that the reactivity changes the compactness of the SEI. But I don't understand why the composition of SEI changes.

The authors should indicate absolute value (intensity) in the elemental composition results, not only concentration. Because even if inorganic layers were same, thick organic layer changes the concentration.

Responses to the Referees' Comments

Reviewer #1 (Remarks to the Author):

The authors synthesized nano-engineered Si-C structure, interface of which is encapsulated by covalent bonding, resulting in great improvement of both capacity retention and Coulombic efficiency of Si-anode based full cells. Various examination viewpoints are considered and systematically organized. That said, there are few things that needs to be clarified prior to me commening on whether or not this work deserves the publicaiton in Nature communicaitons. Thsese include,

R: Thank very much for the referee's recognition of our work.

1. The positive effect of covalent interface is varified comparing SF, SF@G, and SF@G-HF. That said, as far as I notice, there are a few works that fundamentally deliver the covalent concept in previous arts such as Hasan, et. al

https://www.nature.com/articles/ncomms9597;

Osaka et. al

https://pubs.rsc.org/en/content/articlelanding/2014/ta/c3ta13080k#!divAbstract

These works show the covalent effect is particulaly exhibited only when the base Si structure is quite small feature size, such as <10nm, i.e. cell performance improvement by covalent encapsulation is not global but synergized when combined with ultra-thin Si. Also the work,

Ogata et. al

https://www.nature.com/articles/s41467-018-02824-w

shows in-situ stabilization of reversibility, showing the composite turns into covalent encapsulated sub 3nm Si system. The author needs to clarify the intrinsic novelty of the work compared to the previous covalent works.

R: Thank very much for the referee's positive comment (**The positive effect of covalent interface is verified comparing SF, SF@G, and SF@G-HF**) and for the kind reminder which improves our manuscript. As shown in Figure 1d, in the previous literature the introduction of covalent binding shows potential to reduce accidental disconnection of silicon and contributes to the capacity and retention increase at certain rates in terms of weight, volume, or area. What needs to be emphasized is that

the existing strategy is still mainly limited to an inefficient point-mode of binding, incapable of substantially enhancing charge transport kinetics and subsequent rate capability. Furthermore, this covalent binding often encounters severe risks of disruption upon cycling due to the direct contact and propagating erosion of silicon with the electrolyte in the existing covalently-bound hybrids. Different from the existing point-contact-mode of physical and covalent binding, herein, we emphasize the intrinsic novelty of our design (of two-dimensional covalent encapsulation) including: (1) **two-dimensional covalent binding:** ***this creates a robust and efficient contact between the silicon and electrically conductive media, enabling stable and fast electron as well as ion transport from and to silicon***; and (2) **covalent encapsulation:** ***this profoundly changes the interface between silicon and the electrolyte, thus securing the as-created contact to persist upon cycling***. Consistent with the above discussion, for further clarifying the novelty compared to the previous covalent approaches, the text '*Yet, this binding furnishes an inefficient point mode, incapable of substantially enhancing charge transport kinetics. Moreover, it encounters risks of disruption upon electrolyte erosion.*' has been *added* when discussing the existing covalent binding strategy in the revised version. Further, the title of our manuscript '*Stable high-capacity and high-rate silicon-based lithium battery anodes due to covalent encapsulation*' has been *improved* as well into '*Stable high-capacity and high-rate silicon-based lithium battery anodes due to two-dimensional covalent encapsulation*' in the revised edition. The text '*a protocol is developed which we describe as covalent encapsulation.*' has been *improved* into '*a protocol is developed which we describe as two-dimensional covalent encapsulation.*' in the revised Abstract.

Furthermore, we completely agree with the Referee that the downsizing of silicon plays an important role in a series of different electrode material designs. As outlined in the original Introduction part, the silicon design strategies can be classified into three categories: (1) **downsizing of silicon**, (2) **tailoring the adjacent electrically conductive medium of the downsized silicon**, and (3) **covalent binding between the silicon and adjacent electrically conductive media**. For the covalent effect, the

basic point is to ensure operation of silicon building blocks without mechanical fracture. Otherwise, the covalent strategy when applied to fractured and even pulverized silicon components becomes meaningless. This indeed requires downsizing of silicon components to feature sizes on the nanoscale (especially below the critical value as reported previously, for example, in *ACS Nano* 2012, 6, 1522). Considering this synergistic effect, we have indeed emphasized that '***Two-dimensional**, covalently bound silicon-carbon hybrids serve as proof-of-concept of a new material design.*' and '*the **two-dimensional** covalent binding creates a robust and efficient contact between the silicon and electrically conductive media, enabling stable and fast electron as well as ion transport from and to silicon.*' in the original Abstract as well as '*the involved **two-dimensional**, covalent binding creates a robust and efficient contact between the silicon and electrically conductive media, enabling stable and fast electron as well as ion transport from and to the silicon.*' in the original Conclusion part. For becoming even more convincing, the related text of '*iii) covalent binding between silicon and adjacent electrically conductive media with the greatest potential…*' in the revised Introduction part has been *improved* into '*iii) covalent binding between the downsized silicon and adjacent electrically conductive media with the greatest potential…*'. As mentioned above, the title of our manuscript '*Stable high-capacity and high-rate silicon-based lithium battery anodes due to covalent encapsulation*' has been *improved* as well into '*Stable high-capacity and high-rate silicon-based lithium battery anodes due to two-dimensional covalent encapsulation*' in the revised version.

In addition, we have carefully checked and classified the mentioned three literatures as follows:

In the first literature (*Hasan, et. al, https://www.nature.com/articles/ncomms9597; that is, Ref. 44 in the original edition*), interconnected microparticles composed of sulfur-doped graphene (SG) nanosheets that sandwich silicon nanoparticles (SiNP) were fabricated, in which the modified binder (c-PAN) was used to enable a robust electrode structure as well as to prevent penetration and erosion of the electrolyte into the bulk of the electrode and limited most of the SEI formation to the surface. In view

of this material arrangement (zero-dimensional vs. two-dimensional), silicon nanoparticles were covalently bound to the adjacent conductive medium (that is, sulfur-doped graphene) by sulfur atoms in a point-contact mode. This electrical connection was reflected in the literature as well by the text of 'However, those fractured Si particles are still confined within the continuous channels of the c-PAN shell, which is overlaid on SG and maintains the electrical connection between Si and graphene.'. Thus, we have cited this work as Ref. 44 in the original version when discussing the third strategy as shown in Figure 1d.

In the second literature (*Osaka et. al, https://pubs.rsc.org/en/content/articlelanding/2014/ta/c3ta13080k#!divAbstract*), the authors prepared Si–O–C films by electrodeposition and demonstrated excellent cycle durability. From the viewpoint of material engineering, this work represents one pioneering covalent binding, in which individual silicon atoms and/or clusters were covalently bound to one carbon atom by one oxygen atom in a point contact mode, functioning as active sites for lithium storage. We have thus *cited* this original work as *Ref. 48* in the revised version when discussing the third strategy (that is, covalent binding). Consistently, the text '*iii) covalent binding between silicon and adjacent electrically conductive media with the greatest potential to reduce accidental disconnection (Fig. 1d)*[24,44-46]' has been *modified* into '*iii) covalent binding between silicon and adjacent electrically conductive media with the greatest potential to reduce accidental disconnection (Fig. 1d)*[24,45-48]' in the revised version.

In the third literature (*Ogata et. al, https://www.nature.com/articles/s41467-018-02824-w: shows in-situ stabilization of reversibility, showing the composite turns into covalent encapsulated sub 3nm Si system.*), the authors constructed spray-dried second particles of carbon nanotubes and silicon nanoparticles with and without graphite as schematically shown in their Figure 2b. Furthermore, the authors demonstrated their in-situ stabilization based upon the altered nature (that is, a shift from bulk- to <5 nm surface-dominated systems) of the electrode via the repeated phase transformation enabled by precisely controlling the Li–Si lithiation process or DOD%. This stabilization mechanism is consistent with

that revealed in our previous work on graphene-supported ~3 nm silicon quantum dots (Approaching the downsizing limit of silicon for surface-controlled lithium storage. *Adv. Mater.* 2015, 27, 1526-1532 (Ref. 37 in the original edition)), in which surface-controlled lithium storage was disclosed to be responsible for the achieved cycling and rate performance even without any surface modification or encapsulation. Evidently, to electrically connect such tiny silicon nanostructured units to enable performance enhancement, the introduced carbon nanotubes and/or graphene play important roles. As the nano-structuring of silicon with an ultra-small feature size (via either pre-synthesis or in-situ formation) can be classified as the first strategy shown in Figure 1b in our original manuscript, the introduction of either carbon nanotube entangling silicon nanoparticles (that is, surface-dominated networked structures with the small feature size upon DOD%) or graphene supporting silicon quantum dots belongs to the second strategy (that is, tailoring the adjacent electrically conductive medium) as shown in Figure 1c of our original manuscript. Therefore, we have *cited* this literature as *Ref. 44* in the revised version when discussing the second strategy. Consistently, the text '*ii) tailoring of the adjacent electrically conductive medium (Fig. 1c)*[32-43].' has been *modified* into '*ii) tailoring of the adjacent electrically conductive medium (Fig. 1c)*[32-44]' in the revised version.

2. There are hundreds of nano-engineered Si-C composites published for the last 10 years. However, unfortunately, most of them neglect density (g/cc) and volumetric/gravimetric energy density (Wh/L or Wh/kg) after volumet expansion when combined with cathode. Some S-C composites only shows 0.5-1.0 g/cc despite its high capacity such as 2000mAh/g, ending up with 1000-2000mAh/cc. Further, volume expansion needs to be considered. There is an approximately linear correlationship between mAh/cc and volume expansion rate, thus the mAh/cc needs to be devided by volume expansion rate, e.g. if 180%, 2000mAh/cc devided by 1.8 = 1100mAh/cc effectively. This effective number needs to be taken into account into calculation of Wh/L and Wh/kg. Further, with increase of Si % in the anode, discharge voltage drop gets significant due to nature of Cap-Voltage profile of Si. This can

reduce energy density indeed. In the actual commercialization stage, people always realize that after volume expansion and voltage drop in use of higher concentration Si there is not so much energy density gain in view of whole device. So the author needs to clarify advantage of their work in this view point.

R: We thank the referee for his/her kind note and excellent suggestion which strengthen our manuscript. First, as the referee pointed out, nano-structuring of Si has been intensively demonstrated, yet mostly along with a low material density (typically <0.5 g cm^{-3}) and consequent unacceptable volumetric capacity and energy density. We believe that the volumetric capacity (product of packing density and gravimetric capacity) is a critically important parameter for commercialization of Si anodes. It has been estimated in previous reports (e.g., see *Nano Lett.* 2013, 13, 5578; that is, Ref. 22 in the original edition)) that a maximum allowable material density can be 0.8−0.9 g cm^{-3} (depending on the achievable gravimetric capacity), because the essential space must be necessarily involved to accommodate the volume change of Si so as to guarantee the architectural integrity and stability upon cycling. In view of the high gravimetric capacity achieved as well as a packing density reaching ~0.89 g cm^{-3}, the obtained volumetric capacity of SF@G in our work is exceptionally high! This impressive merit of our SF@G design has thus been presented and discussed in the original edition as '*Benefiting from the high gravimetric capacity and the high density of the material,* ***the volumetric capacity of SF@G anodes is extraordinarily high*** *(Fig. 4d, 4e). Considering the whole electrode volume as well as the volume change upon cycling (Fig. S12), SF@G exhibits a volumetric capacity of 2350 mAh cm^{-3} at a rate of 0.8 A g^{-1}* ***which is more than four times that*** *(~550 mAh cm^{-3}) of commercial graphite anodes. Even at high rates of 2, 4, 8, 12, 16, and 20 A g^{-1}, a volumetric capacity of 1952, 1547, 1202, 971, 869, and 694 mAh cm^{-3} is delivered, respectively, which is* ***54%, 74%, 65%, 699%, 1323%, and 1442% of that of SF*** *at the same rates. The achieved volumetric capacity of SF@G is* ***markedly superior to previous results for different silicon anodes***[19,21-24,29,31,32,34,39,40,43] *(Fig. 4e).*'. We therefore note that the relatively high density of SF@G can be related to its micro-sized flower-like architecture, which is also responsible for the high tap/packing density observed in SF

as depicted in our previous work (*ACS Nano* 2017, 11, 7476, that is, Ref. 27 in the original version).

Second, we completely agree with the referee that the volume expansion must be considered for any reported volumetric capacity values. That is, **controlling the volume change at the electrode scale** is critically important toward real application. It is known that an acceptable degree of volume change for a commercialized lithium ion battery electrode is about 10% (Ref. 22). In our case, the cycled SF@G electrode remains almost the same as the initial one with a thickness change of ~5.8% (Fig. S12 in the original supporting information), evidencing that the volume change of SF@G electrodes is significantly below the above limit. This efficient control of volume change at the electrode scale can be attributed to the two-dimensional character and three-dimensional spatial orientation of the nanoplates in SF@G (also see Figure 1e and Figure 2a). These allow for better accommodation of the volume change of silicon without disturbing the material structure and the electrode architecture (see also the details in the Reply to the referee 2). We have thus noted that '*The SF@G and SF@G-HF electrodes show a thickness change of ~5.8 and ~9.3%, respectively.*' in the caption for Fig. S12 in the original supporting information. Moreover, we have considered this critical point for the reported volumetric capacity and mentioned it in the original text as '*Considering the whole electrode volume as well as* ***the volume change upon cycling (Fig. S12)****, SF@G exhibits a volumetric capacity of 2350 mAh cm^{-3} at a rate of 0.8 A g^{-1} which is more than four times that (~550 mAh cm^{-3}) of commercial graphite anodes.*'. To make the advantage of our approach even clearer, the text '*Considering the whole electrode volume as well as the volume change upon cycling (Fig. S12) …*' has been *improved* into '*Considering the whole electrode volume as well as the volume change of ~5.8% upon cycling (Fig. S12) …*' in the revised edition. Furthermore, this merit has been mentioned in the additional text '*It should be noted as well that this structural and interfacial stabilization,* ***combined with minimized electrode thickness variations (Fig. S12)****, signify better accommodation of the volume change of silicon in SF@G, which can be attributed to the two-dimensional character and three-dimensional spatial orientation of the*

involved nanoplates[27].' in the revised edition (See also the details in the response to the Referee 2).

Third, as the referee noted, the discharge voltage drops upon increasing the silicon content in the anode in view of the capacity-voltage profile of silicon, thus reducing the energy density of a full cell. While this voltage drop occurs in our design with a high Si content of ~88% as estimated by thermogravimetric analysis (Fig. S10 in the original supporting information), we have *provided* the estimated specific energy and energy density of SF@G-based and graphite-based full cells pairing with the LFP and LCO cathodes as *Table S1* (see also Ref. 27 in the original version (*ACS Nano* 2017, 11, 7476) for the detailed calculation method), *along with* the voltage profile of full cells and the thickness characterization of the relating cathodes as *Fig. S27* in the revised version. Even if considering the fact that the small volume variation (~5.8%) of SF@G can give rise to a slight decrease in the estimated volumetric values, the competitive specific energy and energy density (Wh/kg and Wh/L, respectively) of SF@G-based full cells relative to graphite-based ones clearly highlight the potential of our design, attributable to its two-dimensional covalent encapsulation (enabling high gravimetric capacity at high rates), as well as relatively high material/packing density and limited volume changes upon cycling. Consistently, the text '*In addition, the viability of SF@G is also characterized by a competitive energy density (Table S1).*' has been *added* in the revised edition.

Last, based on the above facts, this extraordinary merit has been described in the original Abstract part as '*Their **high reversibility, capacity and rate capability furnish a remarkable level of integrated performances when referred to weight, volume and area** which is clearly superior to previous works.*', and emphasized in the original Conclusion part as '*The proof-of-concept of two-dimensional, covalently bound silicon-carbon hybrids exhibits **stable high-capacity and high-rate** lithium storage performances **when referred to weight, volume and area.***'.

Reviewer #2 (Remarks to the Author):

The authors synthesized silicon-graphene hybrids in the skin-like form where Si and

C are covalently bonded and used the hybrids as anode for lithium batteries. They found that the covalent binding makes electrode material superior compared to other Si-C hybrid forms.

R: Thank very much for the referee's recognition of our work.

A central, outstanding issue for silicon anodes is its large volume change upon charge and discharge. The authors acknowledge that. In literature, as long as the silicon loading is sufficiently large to make the volumetric capacity sufficiently high, this mechanics issue arises. However, throughout the manuscript, the authors did not mention about this, despite that they acknowledge volume change is a serious issue in previous studies. It is important to report to the readers and the community how this issue is resolved (or preserved). If this issue remains, i.e., if volume change exists for the Si-C hybrid material, the authors need to address why the volume change does not (or does) influence the performance of the material.

R: Many thanks for the referee's kind note and nice suggestion. As pointed out by the Referee, the large volume change upon charge and discharge is an outstanding issue of silicon anodes; this issue as well as related structural, interfacial, and cyclic instabilities are aggravated while requiring high volumetric capacity. In our design, high reversibility, capacity and rate capability when referred to weight, volume and area have been achieved as exhibited and detailed in the original main text. Noticeably, as exhibited in Figure S12 in the original supporting information, the cycled SF@G and SF@G-HF electrodes show a thickness change of ~5.8 and ~9.3%, respectively. Accordingly, for our SF@G design, the volume change has been significantly suppressed at the electrode scale, while delivering the achieved performance. Yet, we consider that the volume change of silicon inevitably exists at the material building block scale since lithiation and delithiation are involved. In our previous work (*ACS Nano* 2017, 11, 7476-7484; that is, Ref. 27 in the original version), it has been investigated that thin Si nanoplates in the SF mimic the behavior of a planar Si thin film in the respective spatial orientations upon cycling. There, the free spaces (or gaps) between Si nanoplates can well accommodate the volume change of Si, thus stabilizing the material structure and the electrode architecture. Resembling the SF,

the SF@G possesses a micro-sized flower-like architecture composed of many interconnected nanoplates with different spatial orientations. We can thus firmly assume that the two-dimensional character and three-dimensional spatial orientation of the nanoplates in SF@G (also see Figure 1e and Figure 2a) allows for better accommodation of the volume change of silicon without disturbing the material structure and the electrode architecture. This scenario has indeed been confirmed by structural, interfacial, and morphological preservation of cycled SF@G (see SEM, STEM, and elemental mapping images of cycled SF@G in Figure 5a, Figure S15, Figure S19, and Figure S23 in the original edition) as well as the minimized electrode thickness changes after cycling (Figure S12 in the original supporting information). Thereby, whilst the volume change of silicon occurs and the consequent dynamic interface remains upon charging and discharging, our design establishes a robust and efficient contact between the silicon and electrically conductive media. At the same time, it profoundly alters the material interface making the as-created contact persistent to the electrolyte, thus enabling the performance. While these design features (**two-dimensional covalent encapsulation**) have been thoroughly discussed in the whole text, we have *improved* the title of our manuscript as '*Stable high-capacity and high-rate silicon-based lithium battery anodes due to two-dimensional covalent encapsulation*' (see also the Reply to Point 1 of Referee 1).

Furthermore, consistent with the above discussion, the text '*It should be noted as well that this structural and interfacial stabilization, combined with minimized electrode thickness variations (Fig. S12), point toward better accommodation of the volume change of silicon in SF@G. This can be attributed to the two-dimensional character and three-dimensional spatial orientation of the involved nanoplates*[27].' has been *added* in the revised version.

I would be supportive to this manuscript to be accepted by Nature Comm, provided that the above issue is carefully addressed.

R: Thank very much for the referee's recognition of our work and positive comments on our manuscript which strengthen our manuscript.

Reviewer #3 (Remarks to the Author):

The paper demonstrates a nice energy density and electrochemical stability.

R: Thank very much for the referee's recognition of our work.

However, to verify the concept of the atuhros, further investigations are needed. Otherwise, the results are one of good results in the field of silicon based anodes for LIBs. So far, the paper does not reach the level of Nature Commun..

R: Thank very much for the referee's stimulating suggestions which strengthen our manuscript.

In the concept of the authors, the graphene layer is fixed by silicon oxide and carbon bonding. However, when lithiated, SiOx is reacted into Li2O and LixSi. The authors need to verify how the silicon layer and the graphene layer are bonded after lithiation.

R: Thank very much for the referee's comment. First, two-dimensional Si nanoplates and G nanosheets in our SF@G were covalently bound by interfacial Si–O–C bonds (denoted as **SiO_2C_2** and **SiO_3C**), as verified by Si 2p XPS spectrum (Fig. 2h in the original edition). The formation of such bonds has been schematically illustrated in Fig. S9 and discussed as well in the original version as '*The "skin-formation" is suggested to be associated with* ***the native silicon oxide layer*** *on Si nanoplates (Fig. S9). Upon interfacing with the introduced hydrogen gas, the silicon oxide on the Si nanoplates is* ***partially reduced to Si–O intermediates*** *at high temperature. These intermediates are further combined with carbon species derived from methane while additional supply of methane furnishes the deposition of graphene.*'. Based on the Si 2p spectrum of cycled SF@G presented as one panel of Fig. S19a in the original supporting information, it is evident that interfacial Si–O–C bonds (mainly **SiO_2C_2** and **SiO_3C**) remain after cycling, thus maintaining covalent binding between Si nanoplates and G nanosheets. Moreover, this point has been described in the original text as '*It is noteworthy that the covalent binding present in SF@G is well retained whilst its two-dimensional hybrid structure persists upon cycling (Fig. S19, S23).*'. Therefore, besides the partially reduced silicon oxide, the remaining silicon oxide, if there is any, may be converted into Li_2O (or Li_xSiO_y (e.g., $Li_2Si_2O_5$, Li_4SiO_4)) and Si

by typical reactions of $SiO_2 + 4Li^+ + 4e^- \rightarrow 2Li_2O + Si$, $5SiO_2 + 4Li^+ + 4e^- \rightarrow 2Li_2Si_2O_5 + Si$, and/or $2SiO_2 + 4Li^+ + 4e^- \rightarrow Li_4SiO_4 + Si$. As the Li_2O (or Li_xSiO_y (e.g., $Li_2Si_2O_2$, Li_4SiO_4)) species is scattered between Si–O–C bonds near the silicon-carbon interface, the thus-formed Si can be fused or contacted with the bulk Si of SF@G during its reversible lithiation and delithiation via $Si + yLi^+ + ye^- \leftrightarrow Li_ySi$. It should be noted that, there is no distinguishable Li_2O peak (~53 eV in the Li 1s spectrum) and Li_xSiO_y peak (~530 eV in the O 1s spectrum) for cycled SF@G (see Fig. 5d, Ref. 48 in the original edition (*J. Electrochem. Soc.* 2009, 156, A95), and *Phys. Chem. Chem. Phys.* 2015, 17, 24956), which can be related to their insignificant amount.

Second, this preservation of interfacial Si–O–C bonds in our concept may trace back to (or refer to) previously reported SiOC composite materials, in which mixed bonds (e.g., $SiOC_3$, SiO_2C_2 and SiO_3C) of Si, O, and C are usually included along with various contents of free carbon phases. Fukui *et al.* explained the origin of electrochemical lithium storage in carbon-rich SiOC composite materials, in which three electrochemically active sites were observed: interstitial spaces or edges of the graphene layers, directly or indirectly the Si–O–C glass phase, and the micropores. It was further evidenced that, while both the Si–O–C glass phase and micropores are minor as electrochemically active sites for lithium storage, and interstitial spaces or edges of the graphene layers act as major electrochemically active sites in their composite material (*ACS Appl. Mater. Interfaces* 2010, 2, 998). Wilamowska-Zawlocka *et al.* indicated that the amount of free carbon phase (a major lithium storage host) in SiOC ceramics is a crucial factor for the electrochemical activity towards lithium ions (*RSC Adv.* 2016, 6, 104597). Graczyk-Zajac *et al.* rationalized the highly reversible Li-ion storage capacity of SiOC in terms of the enhanced Li-ion storage in the more disordered free carbon phase of SiOC, while this disorder is induced by the presence of mixed SiOC bonds or units (*J. Mater. Chem. A* 2018, 6, 93). For carbon-poor SiOC materials, Liu et al. detailed the electrochemical reversibility of Si–O–C species, where lithium can be *reversibly* inserted into both **SiO_2C_2** and **SiO_3C**, while $SiOC_3$ as well as some SiC_4 are irreversible with lithium

(*Mechanism of lithium storage in Si–O–C composite anodes*, *J. Power Sources* 2011, 196, 10667). In the context of these existing investigations on SiOC materials, the preservation of interfacial bonds observed in our design can be associated with the reversible interaction with lithium, although the detailed mechanism remains to be studied. Following the Referee's nice suggestion, we have studied the interfacial properties of *lithiated* SF@G, SF@G-HF, and SF by XPS. The results have been *provided* as *Fig. S26* in the revised supporting information, which indicates the presence of interfacial Si–O–C bonds interacting with lithium in the lithiated SF@G, confirming the above deduction. Consistently, these discussions have been *summarized* and *provided* as additional *Supplementary note 4* in the revised edition. Furthermore, the text '*It is noteworthy that the covalent binding present in SF@G is well retained whilst its two-dimensional hybrid structure persists upon cycling (Fig. S19, S23).*' has been *modified* into '*It is noteworthy that the covalent binding present in SF@G is well retained whilst its two-dimensional hybrid structure persists upon cycling (Fig. S20, S25, S26, and Supplementary note 4).*' in the revised version.

Supplementary note 4 is also copied here: The preservation of covalent binding between Si nanoplates and G nanosheets in SF@G upon charging and discharging may be attributed to a reversible interaction of the involved Si–O–C species (e.g., SiO_2C_2 *and* SiO_3C*) with lithium, resembling the reversible insertion described previously for Si–O–C bulk materials*[5]*. This scenario is also verified by the XPS results of lithiated SF@G, which indicates the presence of Si–O–C species interacting with lithium (Fig. S26). Furthermore, upon interacting with lithium ions, the remaining* SiO_2 *in each SF@G nanoplate, if there is any, can be converted into* Li_2O *(or* Li_xSiO_y *(e.g.,* $Li_2Si_2O_5$, Li_4SiO_4*)) and Si by reactions of* $SiO_2 + 4Li^+ + 4e^- \rightarrow 2Li_2O + Si$, $5SiO_2 + 4Li^+ + 4e^- \rightarrow 2Li_2Si_2O_5 + Si$, *and/or* $2SiO_2 + 4Li^+ + 4e^- \rightarrow Li_4SiO_4 + Si$*. While the* Li_2O *(or* Li_xSiO_y*) is randomly distributed between already-existing Si–O–C species (or domains) along the silicon-carbon interface as schematically illustrated in Fig. S19, the thus-resulted Si can be further contacted or fused with the silicon bulk of the nanoplate during its reversible lithiation and*

delithiation via $Si + yLi^+ + ye^- \leftrightarrow Li_ySi$. It should be noted that there is no distinguishable Li_2O peak (~53 eV in the Li 1s spectrum) and Li_xSiO_y peak (~530 eV in the O 1s spectrum) for cycled SF@G (Fig. 5d), which rather implies their insignificant amount.

Herein, the additional Ref. 5 is: *Liu, X. et al. Mechanism of lithium storage in Si–O–C composite anodes. J. Power Sources* ***196****, 10667-10672 (2011).*

Acutely, The data of SF@G is superior to the data of SF@G-HF. However, this is not direct evidence to verify the concept of the authors.

R: Thank very much for the referee's recognition and comment of our work. Our design concept is **two-dimensional covalent encapsulation**, that is, including (1) **two-dimensional covalent binding** and (2) **covalent encapsulation** (also, see the Reply to the Referee 1). First, it is known that both silicon and carbon components play critically important roles for the performance enhancement of a silicon/carbon design (also see, *e.g.*, Ref. 5 and Ref. 9 in the original edition). To unravel the binding effect at the silicon/carbon interface, any direct comparisons must be based upon the same hybrid components including silicon and carbon. The SF@G-HF (that is, the SF@G after HF etching to break the covalent binding at the Si/C interface) possesses nearly unchanged two-dimensional silicon and carbon components (Fig. 2 vs. Fig. S8), thereby meeting the above criteria. Second, the structure of SF@G-HF belongs to the second strategy for silicon anodes (that is, *tailoring of the adjacent electrically conductive medium*) as discussed in the original main text. It is known that HF etching has been used to construct diverse hybrid structures of silicon with non-covalently contacted carbon (also see, *e.g.*, *Nat. Nanotechnol.* 2014, 9, 187; *Adv. Mater.* 2013, 25, 3560 (that is, Ref. 32 and Ref. 33 in the original edition)). Upon HF etching, consistently, the SF@G-HF bears an evidently different silicon-carbon contact interface from that of the SF@G (that is, non-covalent *vs.* covalent) as evidenced by Si 2p XPS spectra in Fig. S8f and Fig. 2h in the original version. Thereby, the SF@G-HF holds the ability to act as a reasonable control sample to allow a clear and direct description of the **two-dimensional covalent binding** effect, since there are the

same two-dimensional silicon/carbon components, but different interfacial contacts. A series of comparisons on electrochemical results between *SF@G, SF@G-HF, SF, and other previously reported point-mode contacted silicon/carbon hybrids* (Fig. 4 in the original edition), as recognized as well by the Referee, verify the two-dimensional covalent binding effect: *it creates a robust and efficient contact between the silicon and electrically conductive media, enabling stable and fast electron as well as ion transport from and to silicon.* Third, regardless of interfacial contacts, both SF@G and SF@G-HF belong to a two-dimensional encapsulation design. Impressively, the cycled SF@G and SF@G-HF show markedly different interfacial morphologies and chemical compositions as verified by Fig. 5. They thus directly depict the fact that the interface between the material and the electrolyte is substantially changed. In view of the same encapsulating shells (or media) and testing conditions, combined with the similar interfacial characteristics of cycled SF@G-HF and SF, the observed difference between SF@G and SF@G-HF strongly evidences the distinct role of **covalent encapsulation** in our design: *it profoundly changes the interface between silicon and the electrolyte, thus securing the as-created contact to persist upon cycling*.

The authors explained that the electrolyte penetrates through pinholes and/or defects of graphene without direct contact of Si with the electrolyte. If the ionic pathway is limited like this, the authors need to verify how such high current operation (20 A/g) was achieved.

R: Thank you very much for the referee's comment. First, for nearly ideal graphene with few pinholes and/or defects, the trans-plane transport of lithium ions is limited as the referee pointed out. In view of this fact, **the introduction of in-plane carbon vacancies, defects, and/or pores** into graphene has been demonstrated to be a powerful way to mitigate lithium ion diffusion limitations and consequently enable high current operations (e.g., *Adv. Energy Mater.* 2011, 1, 1079 (**carbon vacancies**); *Nat. Energy* 2016, 1, 15029 (**defects**); *Science*, 2017, 356, 599 (**pores**)). In our design, the in-situ deposited graphene inherently bears numerous pinholes, defects, and/or disordered domains as verified by Raman results (Fig. 2g and Fig. S5 in the original

edition), which rather facilitates ion transport through graphene. This point has thus been described in the original text as '*Furthermore, the ratio of the D band to G band is estimated to be around 1.5, revealing the presence of pinholes, defects and/or disordered domains in the deposited graphene favorable for ion transport (Fig. 2g, S5).*'.

Second, as shown in the Fig. 1e and Fig. 2a as well as discussed in the original whole text, the two-dimensional covalent binding in our design creates a robust and efficient contact between the silicon and electrically conductive media (that is, graphene), thereby enabling stable and fast electron as well as ion transport from and to silicon, even if without a direct contact between the silicon and the electrolyte. Moreover, as for each SF@G building block, lithium ions can be transported from both sides of a hybrid nanoplate as shown in Fig. 2a in the original edition. These distinct hybridization features of our design have been further verified by EIS and CV characterizations as described in the original edition as '*This scenario is nicely supported by the Nyquist plots obtained from electrochemical impedance spectroscopy (EIS) (Fig. S13) and further validated by the significantly improved b values*[37] *of both cathodic (0.21 V) and anodic (0.35 and 0.51 V) peaks of SF@G in comparison with SF@G-HF and SF (Fig. 4g, Fig. S14, and Supplementary note 1).*', thus providing an additional explanation for the achieved high current operations. To become even clearer, the related text '*These properties primarily stem from a robust and efficient contact between silicon and graphene due to the covalent encapsulation and consequent two-dimensional tight binding between Si and C.*' has been *improved* into '*These properties primarily stem from a robust and efficient contact between silicon and graphene at both sides of each nanoplate due to the covalent encapsulation and two-dimensional tight binding between Si and C, although the specific surface area may be an additional factor.*' in the revised version.

Third, it has been reported that, a triple layered a-C/Si/C sandwich thin film electrode (the thickness of C, a-Si, and C: 50 nm, 250 nm, and 50 nm, respectively) can be stably cycled at the current of 100 μA/cm^2 (0.1 mA/cm^2) (Amorphous silicon-carbon based nano-scale thin film anode materials for lithium ion batteries,

Electrochimica Acta 2011, 56, 4717). Considering the specific surface area of our material (SF@G) to be 45.7 m^2 g^{-1} as exhibited in Fig. S3 in the original supporting information, the operating current of 20 A/g can be estimated to be around 0.04 mA/cm^2 when referring to the material surface. From this viewpoint, the allowable high operating currents in our design may be also associated with to the specific surface area of the material itself. As discussed in the above point, the related text has been *improved* as '*These properties primarily stem from a robust and efficient contact between silicon and graphene at both sides of each nanoplate due to the covalent encapsulation and two-dimensional tight binding between Si and C, although the specific surface area may be an additional factor*.' in the revised edition.

Fourth, we note that, there exist various carbon-hybridized nanomaterials showing operations at high currents, such as interconnected porous silicon nanowires coated with ~2 nm carbon layers (enabling lithium storage operation at 7C (24.5 A/g), *ACS Nano* 2019, 13, 2307), holey graphene sheet-networked (or sandwiched) Nb_2O_5/graphene hybrids (enabling lithium storage operation at 100C (20 A/g), *Science*, 2017, 356, 599), double carbon shells (~10 nm in thickness) coated silicon nanoparticles (enabling lithium storage at 5C and even 10C (21 and 42 A/g, respectively), *Adv. Mater.* 2017, 29, 1605650), and multilayer graphene (~10 nm in thickness) caged micrometer-sized silicon particles (enabling lithium storage at 4C (16.8 A/g), *Nat. Energy* 2016, 1, 15029). All these mentioned studies generally reveal the feasibility of fast lithium transport from and to active nanostructures through a carbon-based media/layer with the tailored morphology, structure, and/or thickness.

These are critical points. And small questions are followings.

The authors had better to explain what b value is.

R: Regarding the b value, the detailed explanation has been provided in the original Supplementary note 1 as '*It is known that the voltammetric response of active electrode materials at various sweep rates can be summarized according to the equation* $i = a \times v^b$, *in which the current* (i) *obeys a power law relationship with the sweep rate* (v), *as well as a and b are adjustable values. It has been well established*

in previous investigations of other energy storage electrode materials that there are two well-defined conditions: b = 0.5 and b = 1.0[2-4]. *For b = 0.5, the current in a cyclic voltammetry (CV) experiments varies with the square root of the sweep rate, which reveals a typical battery behavior controlled by semi-infinite linear diffusion. The other defined condition of b = 1 implies that the current is proportional to the sweep rate, which reveals a surface-controlled charge storage process.*'.

For further clarity, the text '*…and further validated by the significantly improved b values*[37] *of both cathodic (0.21 V) and anodic (0.35 and 0.51 V) peaks of SF@G in comparison with SF@G-HF and SF (Fig. 4g, Fig. S14, and Supplementary note 1).*' has been *improved* to '*…and further validated by the significantly improved b values (defining the relation of peak current to sweep rate)*[37] *of both cathodic (0.21 V) and anodic (0.35 and 0.51 V) peaks of SF@G in comparison with SF@G-HF and SF (Fig. 4g, Fig. S14, and Supplementary note 1).*' in the revised version.

The magnitude of Fig 5a-c is not enough to tell us the flower-like structure.

R: Thank you very much for the Referee's kind note. The SEM images of cycled samples with different magnifications have been provided in Fig. 5a-c as well as Fig. S15a-c in the original edition. We have *replaced* the original images in *Fig. 5a-c* with respective higher-magnification ones in the original *Fig. S15a-c* in the revised edition. The original images in *Fig. 5a-c* have been *moved* as well to *Fig. S15a-c* in the revised supporting information. Consistent with this change, the *Fig. 5* and *Fig. S15* have been *updated* in the revised edition.

Furthermore, we note that the architecture of SF@G resembles *hydrangea* flowers in nature. This is in line with its precursor (that is, SF), which has been depicted in our previous work (*ACS Nano* 2017, 11, 7476-7484; that is, Ref. 27 in the original edition). The text '*SF@G possesses a micro-sized flower-like architecture composed of many interconnected nanoplates.*' has been *improved* to '*SF@G possesses a micro-sized hydrangea flower-like architecture composed of many interconnected nanoplates.*' in the revised edition.

In addition, we note that, as the cycled SF@G retains the original flower-like

architecture, the flower-like architecture of cycled SF@G-HF is blurred and the flower-like appearance of cycled SF is nearly deformed, suggesting the distinct interface of our design. The detailed description has been provided in the original edition as '*It is obvious that the SF@G retains the original flower-like architecture upon cycling, possessing a thin and smooth interfacial morphology. Different from that, the flower-like architecture of cycled SF@G-HF is blurred, with an abundance of rather rough SEI deposits filling up the gaps between SF@G-HF nanoplates. In case of cycled SF, the flower-like appearance is nearly completely deformed.*'.

The SEIs on SF@G and "SF@G-HF and SF" are different. I understand that the reactivity changes the compactness of the SEI. But I don't understand why the composition of SEI changes.

R: Thank you very much for the Referee's kind note. As shown in the original Fig. 5, the chemical compositions of SEI are evidenced to be different in SF@G in comparison with SF@-HF and SF. In the original text, we have noted this difference as '*These features clearly characterize the material interface at which electrochemical reduction and consumption of the electrolyte solvents (EC and DEC in this work) occur*[47-49].'. To better clarify the possible origin for this change in SEI, we have *provided* a schematic illustration of the interface-dependent formation of SEI as *Fig. S19* as well as *Supplementary note 2* in the revised supporting information.

Supplementary note 2 is copied here: *As shown in Fig. S19, it is proposed that the observed distinct alteration in SEI components of SF@G and control samples (SF@G-HF, SF) may mainly result from different reduction reactions of the used solvents (e.g., EC, DEC). Specifically, as for EC, its reduction is generally considered via ring opening as shown in reaction (1), followed by further electron transfer reactions, for example, reaction (2). Due to the covalent encapsulation in SF@G fundamentally blocking direct contact of Si with the electrolyte, the product of reaction (1) forms and accumulates near the relatively stable G interface. Thereby, the product of the successive reaction (2) can readily react with the already-existing product of reaction (1) to produce semi-organic carbonates, for example, by reaction*

(3) before interacting with lithium ions. By a further one-electron reduction, these carbonate species can be converted into either lithium alkoxides (ROLi) or radical esters that can coordinate with lithium to form lithium carboxylates (ROCOLi). In contrast, in the case of SF@G-HF and SF with silicon directly interfacing with the electrolyte, the product of reaction (1) will be randomly scattered away from the localized dynamic Si interface during lithiation. Thereby, the neighboring lithium ions have more chances to combine the product of successive reaction (2), resulting in the formation of Li_2CO_3 via reaction (4). Furthermore, the direct contact of silicon with the electrolyte in SF@G-HF and SF also leads to the propagation of other inorganic SEI components such as partially decomposed $LiPF_6$ constituents (Li_xPF_y and $Li_xPO_yF_z$) by repeated reactions of electrolyte anions (PF_6^-) on dynamic Si interfaces, and siloxane structures (R–Si–OR') upon severe corrosion of Si by the electrolyte solvents.

Consistently, the text '*These features clearly characterize the material interface, at which electrochemical reduction and consumption of the electrolyte solvents (EC and DEC in this work) occur*[47-49].' has been *modified* into '*These features clearly characterize the material interface, at which electrochemical reduction and consumption of the electrolyte solvents (EC and DEC in this work) occur (Fig. S19, and Supplementary note 2)* [47-49].'

The authors should indicate absolute value (intensity) in the elemental composition results, not only concentration. Because even if inorganic layers were same, thick organic layer changes the concentration.

R: Thank you very much for the referee's kind note. First, elemental concentrations have been generally used as an effective means to reveal the difference in interfacial components (e.g., *J. Phys. Chem. C* 2012, 116, 19737 (that is, Ref. 47 in the original edition); *J. Power Sources* 2009, 189, 1132 (that is, Ref. 49 in the original edition); *J. Electrochem. Soc.* 2007, 154, A515; *Phys. Chem. Chem. Phys.* 2015, 17, 24956). In the original edition, SEI components have been identified by carefully analyzing XPS results in Fig. 5d-f. This identification has also been schematically shown in Fig. 5h,

with the relating text as '*As schematically shown in Fig. 5h, the* ***main*** *interfacial ingredients of cycled SF@G-HF and SF consistently include Li_2CO_3, LiF, Li_xPF_y, $Li_xPO_yF_z$, and R–Si–OR'; the interface of cycled SF@G* ***mostly*** *consists of organic species (e.g., PEO, ROLi, ROCOLi), with an almost negligible amount of inorganic materials.*' in the original edition. This distinct difference in major interfacial components is described further by the interfacial elemental compositions (or atom concentrations) of the cycled samples as shown in Fig. 5g. The related text in the original version is as follows: '*In the cycled SF@G, the majority is C (~77 at%), along with a low concentration of O and Li as well as an insignificant concentration of F and P, proving the organic nature of SEI in SF@G. In comparison with SF@G, the cycled SF@G-HF and SF display similar atom concentrations corresponding to their inorganics-dominated SEI, where the concentration of C is substantially decreased to ~37 and ~32 at%, respectively, with the concentration of O, Li, F, P, and Si significantly increased.*'. That is, the obtained elemental compositions based upon interfacial atom concentrations are in line with the major interfacial components identified by XPS. For being more accurate, the text '*In the cycled SF@G, the majority is C (~77 at%), along with a low concentration of O and Li as well as an insignificant concentration of F and P, proving the organic nature of SEI in SF@G.*' has been *improved* into '*In the cycled SF@G, the majority is C (~77 at%), along with a low concentration of O and Li as well as an insignificant concentration of F and P, proving the organic-dominated nature of SEI in SF@G.*' in the revised edition.

Second, we agree with the Referee' supposition that a thick organic layer changes the concentration even if inorganic layers are the same. In the cycled SF@G, the inorganic components have been identified as a minor interfacial part by XPS analyses, if they exist. The significant change in interfacial atom concentrations (Fig. 5g) has further evidenced the presence of organic components as the major interfacial species. Given the same inorganic layers, the combination of inorganic and organic interfacial components in SF@G will make the morphology more blurred than those of SF@G-HF and SF (possessing inorganic components as the interfacial majority). Yet, this is not the case as exhibited in Fig. 5a-c and Fig. S15 in the original edition.

Last, we have investigated elemental compositions of individual cycled SF@G and SF@G-HF nanoplates by energy dispersive X-ray spectroscopy (EDX), and *provided* the results as additional *Fig. S23* in the revised edition. The elemental compositions and variations acquired by EDX are in line with the XPS results shown in Fig. 5. Specifically, the details have been *included* in the caption for the Fig. S23 as '***Figure S23 | a*,*b***, *EDX patterns of cycled SF@G (**a**) w/o and (**b**) w/ HCl washing. **c**,**d**, EDX patterns of cycled SF@G-HF (**c**) w/o and (**d**) w/ HCl washing. **e**, Weight ratios of cycled SF@G and SF@G-HF w/o and w/ HCl washing. **f**,**g**, Comparison of weight ratios between pristine and cycled (**f**) SF@G and (**g**) SF@G-HF w/o and w/ HCl washing. Note that the relative value of incremental weight ratios of C to O in cycled SF@G and SF@G-HF relative to their pristine counterparts is 4 and 2, respectively. This discrepancy discloses the average stoichiometry of distinct interfacial SEI species in both cases as revealed by XPS (Fig. 5d, 5e, and 5g) in view of the identical silicon and carbon components in their pristine counterparts. Furthermore, the significant change in weight ratios of C and O in cycled SF@G-HF upon HCl washing can be associated with its inorganic-dominated interface. In stark contrast, the change in weight ratios of C and O in cycled SF@G upon HCl washing is indistinguishable which can be attributed to its organic-dominated interface and stable nature. In addition, a slight increase in weight ratios of C and O in cycled SF@G relative to its pristine one suggests the presence of a low amount of SEI components, in good agreement with the SEM observation (Fig. 5a). By comparison, the observed substantial increase in weight ratios of C and O in cycled SF@G-HF indicates the introduction of a large amount of SEI species as exhibited in Fig. 5b.*' in the revised version. Consistent with the above discussion, the text '*The differences discussed above are also revealed by elemental compositions obtained from EDX analyses (Fig. S23).*' has been *added* as well in the main text when discussing interfacial elemental compositions in the revised version.

Reviewers' comments:

Reviewer #1 (Remarks to the Author):

The author gives a reasonable explanation for the effect of covalent bonding on the cycle performance and for the difference of its nature from previous works. That said, the authors still need to clarify some critical points and provide the following dataset, without which it is hard to say the work can give a reasonable impact to the silicon-based battery community as well as the corresponding industry. From an industry viewpoint, Si-C composite, as well as pre-lithiated SiOx-C composite, is already commercialized and battery manufacturers are day-by-day increasing the Si concentration: currently up to 5w% of 1700-2500mAh/g Si-C and/or prelithiated SiOx-C composite is already mixed in the commercial products, and the ratio is to be increased to 7-10w% in a few years. Thus, without specifically clarifying and shaping the point of the finding, the work cannot be distinguished from many of previously elaborated nano-engineered Si-C works which is never put on the market. In other words, by fully addressing the points below and if the consequently extrapolated numbers are meaningful compared to the state-of-the-art, I think the editor work may want to consider this work qualifies the publication in Nature Communications.

1. Please provide a plot of “mAh/cc vs. volume expansion (VE) rate at fully litiated state” so that the 2D structure in this work has intrinsically different nature of volume expansion from the prior-arts.

As I mentioned earlier, mAh/cc and VE rate (at fully lithiated state) have some linear correlation. As far as I notice this trend is more universal regardless of Si/C composite structures (0D, 1D, 2D, and 3D). So, the authors need to provide direct evidence that the 2D material here has intrinsically different natures of VE from the prior arts. For this, it is very important for the authors to set a reasonably higher areal-capacity loading level (typically >5mAh/cm2, which is the current Li-ion industry standard) in the system. Also, when the authors make such a plot, the authors need to clarify a specific capacity of the anodes (mAh/g) and corresponding volumetric density (g/cc), multiplying these leads to mAh/cc. Please provide at least three points for mAh/cc to extrapolate linear correlation.

2. Please provide the volume expansion rate under the following definition

(Intrinsic volume expansion rate) = 100*(A thickness of fully lithiated anode at 100cycle)/(A thickness of as-is electrode at 0 cycle)

The way the authors define the volume expansion in the text Figure S12 does not deliver intrinsic insights into the volume expansion when the cell is assembled into a commercially compatible cell. The volume change needs to be referenced to the thickness when the electrode is fully lithiated at the target final cycle. Please fix the areal-capacity more than 5mAh/cm2 and the volumetric capacity more than 2000mAh/cc, and then please calculate the volume expansion rate exactly in the way that I propose. If the expansion is still less than 5-10% at fully lithiated state after 100cy compared to the anode thickness at 0 cycle, that is very impressive.

3. The author should amend the way energy density (Wh/kg) is tabulated in Table S1.

The battery industry never calculates the density just based on active materials. For example, the density in the state of the art LCO vs Gr+3w% SiOx commercial full cell is 260Wh/kg at 3500mAh cell level, which has a huge deviation from what the authors provide in the table. Providing such parameters has actually meaningless. So, please re-calculate the density based on the following parameters.

Cathode areal capacity: >5mAh/cm2

Cathode specific capacity (NCA): 198mAh/g
Cathode press density: 3.5g/cc
Cathode active material ratio: 96w%
Al thickness: 12um
Separator thickness: 9um (50% porosity of PE, 2side 1.5um MgO+PvDV coating)
Pouch areal density: 0.05g/cm2
Anode specific capacity: 2000mAh/g
Anode active material rate: 96w%
Negative/positive electrode ratio: 1.05
Anode density: define-by-yourself g/cc
Cu thickness: 8um
Electrolyte amount: 1.8cc/Ah
Full cell voltage window: 3.0-4.3V (you can set 2.5-4.3V, but lowering the voltage can degrade Si-based anode more rapidly)
Nominal discharge voltage to calculate Wh: 3.7-3.75V (the more Si the more voltage drop you expect in the full cell)

Reviewer #3 (Remarks to the Author):

The authors responded my comments well. Therefore, the paper is acceptable for publication. Congratulation.

Responses to the Referees' Comments

Reviewer #1 (Remarks to the Author):

The author gives a reasonable explanation for the effect of covalent bonding on the cycle performance and for the difference of its nature from previous works. That said, the authors still need to clarify some critical points and provide the following dataset, without which it is hard to say the work can give a reasonable impact to the silicon-based battery community as well as the corresponding industry. From an industry viewpoint, Si-C composite, as well as pre-lithiated SiOx-C composite, is already commercialized and battery manufacturers are day-by-day increasing the Si concentration: currently up to 5w% of 1700-2500mAh/g Si-C and/or prelithiated SiOx-C composite is already mixed in the commercial products, and the ratio is to be increased to 7-10w% in a few years. Thus, without specifically clarifying and shaping the point of the finding, the work cannot be distinguished from many of previously elaborated nano-engineered Si-C works which is never put on the market. In other words, by fully addressing the points below and if the consequently extrapolated numbers are meaningful compared to the state-of-the-art, I think the editor work may want to consider this work qualifies the publication in Nature Communications.

R: Thank very much for the Referee's very kindly reviewing of our manuscript. We are glad to address the Referee's remaining concerns from the industrial viewpoint, while the key scientific point and core feature of this manuscript (two-dimensional covalent encapsulation) have been evidenced based on the original data and discussion as the Referee recognized (*a reasonable explanation for the effect of covalent bonding on the cycle performance and for the difference of its nature from previous works*).

In the revised version, the dataset suggested by the Referee has been embodied, which, we believe, would greatly strengthen the manuscript especially from the viewpoint of industrial battery applications.

While many electrode- and cell-level factors (such as electrode additives and other electrode engineering constraints) must be addressed before industrial deployment of SF@G (as well as other reported electrode materials) toward various

cell formats and application requirements, this manuscript has developed and evidenced a new two-dimensional covalent encapsulation strategy for stable and high-performance Si-C electrode materials, thus providing insight into the material design and material construction for energy chemistry, energy material, as well as battery community. Although it is unrealistic to range from the material design, material preparation, electrode performance, cell optimization, and practical deployment, we believe the revised manuscript (*with its feature evidenced from both the scientific and industrial viewpoint*) has met the criteria for publication in Nature Communications, with your wonderful help of kindly comments and invaluable suggestions. Thank you very much again.

1. Please provide a plot of "mAh/cc vs. volume expansion (VE) rate at fully litiated state" so that the 2D structure in this work has intrinsically different nature of volume expansion from the prior-arts.

As I mentioned earlier, mAh/cc and VE rate (at fully lithiated state) have some linear correlation. As far as I notice this trend is more universal regardless of Si/C composite structures (0D, 1D, 2D, and 3D). So, the authors need to provide direct evidence that the 2D material here has intrinsically different natures of VE from the prior arts. For this, it is very important for the authors to set a reasonably higher areal-capacity loading level (typically >5mAh/cm2, which is the current Li-ion industry standard) in the system. Also, when the authors make such a plot, the authors need to clarify a specific capacity of the anodes (mAh/g) and corresponding volumetric density (g/cc), multiplying these leads to mAh/cc. Please provide at least three points for mAh/cc to extrapolate linear correlation.

R: The plot of volumetric capacity vs. intrinsic volume expansion rate has been *presented* as *Figure S12c* in the revised version.

As exhibited in Figure S12c, at a slightly elevated areal capacity and packing density (~5 mAh cm^{-2} and ~1.1 g cm^{-3}), the estimated intrinsic volume expansion rate of SF@G merely varies ranging from 105.9% to 106.6% with the volumetric capacity.

Thus, it is hard to read a linear correlation as mentioned by the Referee, although

other previously-reported silicon materials may appear. This feature can be associated with the two-dimensional character and three-dimensional random spatial orientation of the involved nanoplates in SF@G. In the original version, the related discussion has been presented as '*It should be noted as well that this structural and interfacial stabilization, combined with minimized electrode thickness variations (Fig. S12), point toward better accommodation of the volume change of silicon in SF@G. This can be attributed to the two-dimensional character and three-dimensional spatial orientation of the involved nanoplates*[27]*.*'.

For being convenient, the revised Figure S12 and its updated caption (*changes in red color*) are copied here:

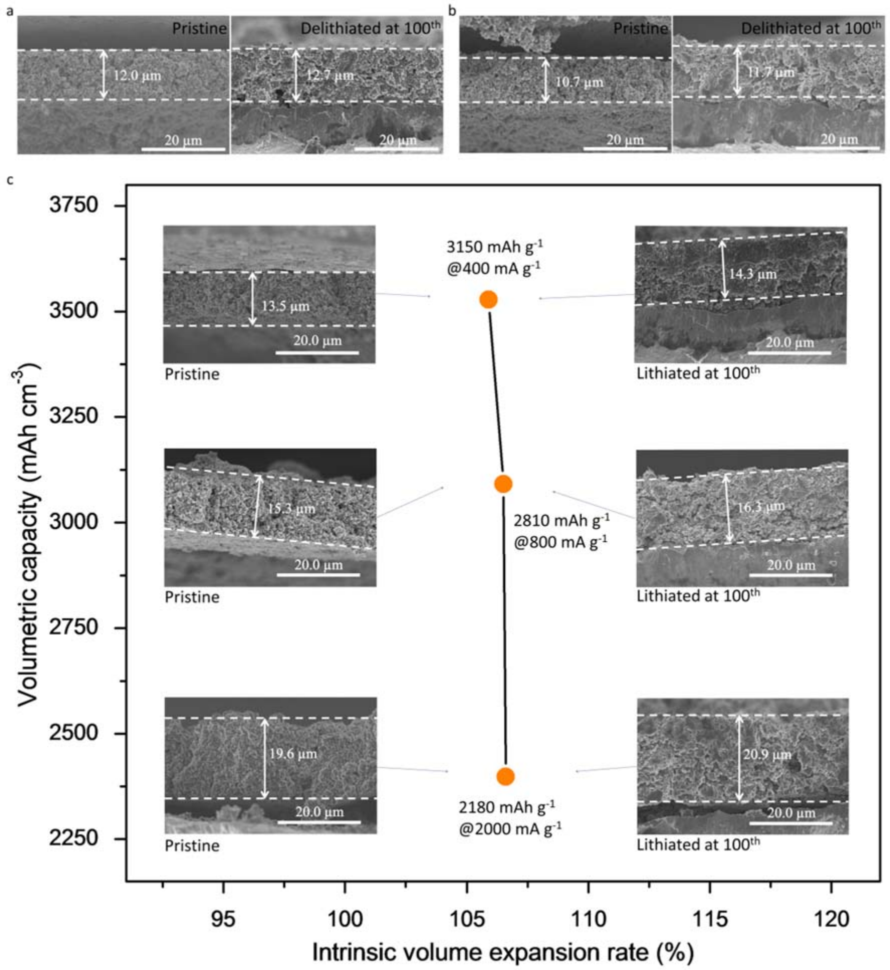

Figure S12 | a,**b**, SEM images of the cross section of a SF@G electrode (**a**) and (**b**) a SF@G-HF electrode in the annotated states. The SF@G and SF@G-HF electrodes show a thickness change of ~5.8 and ~9.3%, respectively. **c**, Plot of volumetric

capacity *versus* Intrinsic volume expansion rate of SF@G electrodes with a slightly elevated areal capacity and packing density (~5 mAh cm^{-2} and ~1.1 g cm^{-3}, respectively), showing intrinsic volume expansion rates of 105.9~106.6%. The inset shows SEM images of the cross section of SF@ electrodes in the annotated states. *Note*: the intrinsic volume expansion rate is defined as an electrode thickness value in the full-lithiated state at 100th cycle divided by its pristine one.

2. Please provide the volume expansion rate under the following definition (Intrinsic volume expansion rate) = 100*(A thickness of fully lithiated anode at 100cycle)/(A thickness of as-is electrode at 0 cycle)

The way the authors define the volume expansion in the text Figure S12 does not deliver intrinsic insights into the volume expansion when the cell is assembled into a commercially compatible cell. The volume change needs to be referenced to the thickness when the electrode is fully lithiated at the target final cycle. Please fix the areal-capacity more than 5mAh/cm2 and the volumetric capacity more than 2000mAh/cc, and then please calculate the volume expansion rate exactly in the way that I propose. If the expansion is still less than 5-10% at fully lithiated state after 100cy compared to the anode thickness at 0 cycle, that is very impressive.

R: Following the evaluation way kindly suggested by the Referee, the intrinsic volume expansion rates of SF@G has been summarized and *provided* as *Figure S12c* in the revised version.

While the volume variation of SF@G upon cycling is 5.8% based upon the original data for the delithiated electrode (Figure S12a), the volume change value based on lithiated SF@G electrode slightly increases to 5.9~6.6%.

Consistent with the original result shown in the original Figure S12, it is obvious that, the additionally-presented intrinsic volume expansion rates further evidence the merit of our SF@G. This can be associated with its two-dimensional character and three-dimensional spatial orientation of the involved nanoplates. In the original edition, the related discussion has been presented as '*It should be noted as well that this structural and interfacial stabilization, combined with minimized electrode thickness variations (Fig. S12), point toward better accommodation of the volume change of silicon in SF@G. This can be attributed to the two-dimensional character*

and three-dimensional spatial orientation of the involved nanoplates[27]*.*’.

For being accurate, the text of ‘*Considering the whole electrode volume as well as the volume change of ~5.8% upon cycling (Fig. S12)*’ has been *improved* into ‘*Considering the whole electrode volume as well as the volume change of 5.8~6.6% upon cycling (Fig. S12)*’ in the revised main text.

3. The author should amend the way energy density (Wh/kg) is tabulated in Table S1. The battery industry never calculates the density just based on active materials. For example, the density in the state of the art LCO vs Gr+3w% SiOx commercial full cell is 260Wh/kg at 3500mAh cell level, which has a huge deviation from what the authors provide in the table. Providing such parameters has actually meaningless. So, please re-calculate the density based on the following parameters.

Cathode areal capacity: >5mAh/cm2

Cathode specific capacity (NCA): 198mAh/g

Cathode press density: 3.5g/cc

Cathode active material ratio: 96w%

Al thickness: 12um

Separator thickness: 9um (50% porosity of PE, 2side 1.5um MgO+PvDV coating)

Pouch areal density: 0.05g/cm2

Anode specific capacity: 2000mAh/g

Anode active material rate: 96w%

Negative/positive electrode ratio: 1.05

Anode density: define-by-yourself g/cc

Cu thickness: 8um

Electrolyte amount: 1.8cc/Ah

Full cell voltage window: 3.0-4.3V (you can set 2.5-4.3V, but lowering the voltage can degrade Si-based anode more rapidly)

Nominal discharge voltage to calculate Wh: 3.7-3.75V (the more Si the more voltage drop you expect in the full cell)

R: Following the Referee’s nice suggestion and detailed note, we have *amended* the

Table S1 in the revised version, while the material-scale energy values are generally adopted to evidence the material design and potential from the scientific viewpoint (e.g., Nat. Energy 2016, 1, 16113).

As shown in the revised Table S1 and its updated caption, the cell-level specific energy has been evaluated for the LFP- and LCO-involved full cells based on previously reported material-to-cell conversion factors of 59~61% (e.g., see Adv. Energy Mater. 2019, 9, 1803170; J. Solid State Electrochem. 2017, 21, 1939). The results exhibit 26 and 28% increase for LFP//SF@G and LCO//SF@G when compared to their graphite-based counterparts, respectively. In addition, upon a NCA-involved full cell with the parameters mentioned by the Referee, the specific energy and energy density has been projected at both the material scale and the cell scale. Specifically, the NCA//SF@G shows an increase of up to 41% in the cell-level specific energy, when compared to that of the current state-of-the-art LCO//graphite (w/ 3wt% SiO_x) commercial full cell (~260 Wh/kg at 3500 mAh cell level).

It is obvious that, the direct comparison of cell-level energy values between SF@G-based full cells and graphite-based ones reveals the successful design of our SF@G, consistent with the conclusion of this manuscript from the original material-level values. Moreover, the estimated energy levels with a current state-of-the-art NCA-involved full cell configuration point to the viability and potential of SF@G, which, we believe, further strengthens our manuscript from the viewpoint of practical applications.

For being convenient, the revised Table S1 and its updated caption (*changes in red color*), reflecting the above discussion and related reference, are copied here:

**LFP, LCO cathode-involved full cells**				
**Electrode configuration**				
	SF@G	Graphite	LFP	LCO
**Electrode area (cm^2)**	1.13	1.13	1.13	1.13
**Mass loading ($mg\ cm^{-2}$)**	1.34	7.14	16.96	15.9
**Electrode thickness (μm)** [a]	12	48	67	45
**Full cell characteristic**				
	LFP// SF@G	LFP// Graphite	LCO// SF@G	LCO// graphite

**Operation voltage (V)** [b]	3.1	3.2	3.7	3.83
**Specific energy (Wh/kg)** [c]	**433**	**338**	**548**	**423**
**Specific energy (Wh/kg)$_{cell}$** [d]	**255**	**202**	**323**	**253**
**Energy density (Wh/L)** [e]	**802**	**567**	**1326**	**838**

**NCA cathode-involved full cells (5 mAh/cm^2)**		
**Electrode and cell configuration**		
	SF@G	NCA
**Specific capacity (mAh g^{-1})**	2000	198
**Mass loading (mg cm^{-2})**	2.73	26.6
**Electrode press density (g cm^{-3})**	0.94	3.5
**Active material weight ratio (%)**	96	96
**Electrode thickness (μm)**	29	75.9
**Current collector thickness (μm)**	8 (Cu)	12 (Al)
**N/P ratio**	1.05	
**Separator thickness (μm)**	9 (50% porosity of PE with two sides of 1.5 μm MgO and PVDF coating)	
**Electrolyte amount (cm^3 Ah^{-1})**	1.8	
**Full cell characteristic**		
	NCA//SF@G	
**Operation voltage (V)**	3.7	
**Specific energy (Wh/kg)** [c]	**667**	
**Energy density (Wh/L)** [e]	**1790**	
**Specific energy (Wh/kg)$_{cell}$**	**394** [f]	**367** [g]
**Energy density (Wh/L)$_{cell}$**	**770** [f]	**850** [h]

Table S1 | Electrode configurations and estimated[1] specific energy and energy density levels of full cells. Considering the same cathode (LFP or LCO), the comparison of specific energy and energy density at the material scale[2] and the cell scale between SF@G-based full cells and graphite-based ones reveals the successful design of SF@G, the projected energy level in a current state-of-the-art NCA-involved cell configuration further points to the viability and potential of SF@G. *Note*: a. The electrode thickness for LFP and LCO is acquired by SEM observation as shown in Fig. S27. b. The operation voltage is obtained based upon the voltage profiles of full cells as exhibited in Fig. S27. c. The specific energy is in terms of both the anode and cathode. d. The estimated specific energy in terms of the whole cell based on previously reported material-to-cell factors of 59~61% (which can be further increased depending on cell formats)[3,4], pointing to 26 and 28% increase for LFP//SF@G and LCO//SF@G when compared to their counterparts, respectively. e. The energy density is on the basis of the total volume of the anode and the cathode; the volume variation (5.8~6.6%) of SF@G upon cycling can incur a slight decrease in the estimated values. f. The estimated specific energy and energy density in terms of the whole cell based on a typical material-to-cell ratio for an 18650-type cell (59 and 43%, respectively)[3]. g. The evaluated specific energy in terms of the whole cell based on the displayed cell configuration and components, indicating an increase of up to

41% when compared to that of the current state-of-the-art LCO//graphite (w/ 3wt% SiO_x) commercial full cell (~260 Wh/kg at 3500 mAh cell level). h. The calculated energy density in terms of the whole cell based on the displayed cell configuration and components (w/o considering cell housing, poles, and tabs).

Reviewer #3 (Remarks to the Author):

The authors responded my comments well. Therefore, the paper is acceptable for publication. Congratulation.

R: We really appreciate your recommendation to publish our manuscript in Nature Communications.

Reviewer #4 (Remarks to the Author):

The authors report a two-dimensional covalent binding strategy to improve Li storage performance of SF@G by enhancing the interface stability and forming conductive framework in SF@G. And the Si-O-C is very important for the Si-C hybrid materials to establish a robust and efficient electrical contact. The SF@G exhibited much improved electrochemical performances compared to SF@G-HF and SF. Although the paper is well written, the idea to form the area-to-area efficient electrical contact between Si and C by Si-O-C is not novel and the work cannot be distinguished from many of previously elaborated nano-engineered Si-C works such as J. Mater. Chem. A, 2019, 7, 12763–12772. The authors demonstrate that SF@G, SF@G-HF and SF could form different SEI, which is very interesting. However, the SEI components and distributions in Figure 5h are only deduced from the XPS results, which are not enough. The authors should provide more evidences such as cryo-EM to support the assumption. The current version is not acceptable for publication in Nature Communications. Some detailed comments as following.
(1)The SF@G displays a very small volume change of 5.8~6.6%(Figure S12), which is very important for the practical applications of Si anodes, the authors should provide more discussions and explanations
(2)What is the thickness of Si nanoplates? Since the authors insist that graphene nanosheets deposited on SF are typically 2~3 layers, however, the C content in SF@G is about 12 % as estimated by thermogravimetric analysis (TGA).
(3)In Figure 4a, the SF@G and SF@G-HF have capacities about 2200 mAh g-1, however, in the long-term cycle in Figure 3c, their capacities are below 2000 mAh g-1, why?
(4)Line 129-130, SF@G also offers a dramatically improved cycling stability at a high rate 130 of 2 A g-1 over 500 cycles compared to SF@G (here, the SF@G should be changed as SF@G-HF) and SF,
(5)If the author investigated the effect of morphology and structure of graphene coating on the electrochemical properties of SF@G? the Raman results reveal the the ratio of D band to G band (ID/IG) to be around 1.5, if this is the optimized structure of graphene coating?
(6)The SF@G exhibits larger b values in Figure 4g, however, the SF@G-HF and SF have the similiar b values. The authors should provide more explanations since the SF@G-HF and SF@G have the similar morphology and structure except Si-O-C bonding.
(7)What is the tap density of SF@G? In Table S1, the electrode press density of SF@G is only 0.94 g cm-3.
(8)The um should be corrected as μm in main text and supporting informations

Responses to the Referees' Comments

Reviewer #4 (Remarks to the Author):

The authors report a two-dimensional covalent binding strategy to improve Li storage performance of SF@G by enhancing the interface stability and forming conductive framework in SF@G. And the Si-O-C is very important for the Si-C hybrid materials to establish a robust and efficient electrical contact. The SF@G exhibited much improved electrochemical performances compared to SF@G-HF and SF.

R: Thank very much for the Referee's recognition of our work. All the comments have been carefully addressed point-by-point as below, which further strengthens our manuscript.

Although the paper is well written, the idea to form the area-to-area efficient electrical contact between Si and C by Si-O-C is not novel and the work cannot be distinguished from many of previously elaborated nano-engineered Si-C works such as J. Mater. Chem. A, 2019, 7, 12763–12772.

R: Thank very much for the Referee's comment. **The novelty of two-dimensional covalent encapsulation** in our work has been thoroughly discussed and evidenced in the original main text, as well as recognized by the Referee 1, 2, and 3. As the Referee knows, the binding between Si and the adjacent electrically conductive media and consequently the electrode framework is a critical issue, in particular, when employing conventional electrode formulation with known conductive additives and binders. As shown in the original Figure 1 and also recognized by the Referee, distinctly different from the existing point-contact-mode of physical and covalent binding in previously elaborated nano-engineered Si-C works, we emphasize the **intrinsic novelty of our design (of two-dimensional covalent encapsulation)** including: (1) **two-dimensional covalent binding: only *this creates a robust and efficient contact between the silicon and electrically conductive media, enabling stable and fast electron as well as ion transport from and to silicon***; and (2) **covalent encapsulation: *this profoundly changes the interface between silicon and the electrolyte, thus securing the as-created contact to persist upon cycling***.

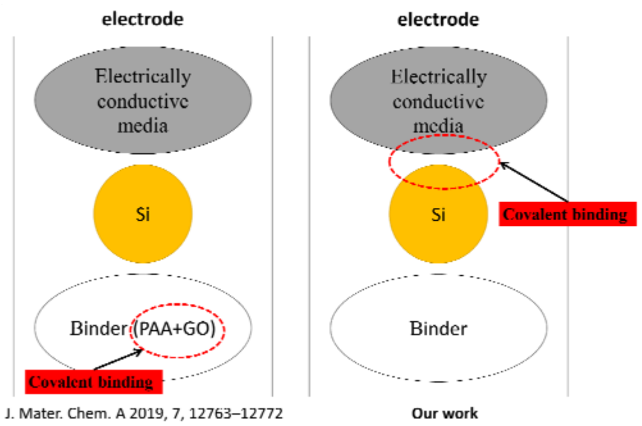

Furthermore, it should be pointed out that, as illustrated in the above scheme, the work by Prof. Seong-Hyeon Hong (*J. Mater. Chem. A* 2019, 7, 12763–12772) shows potential of mechanically robust covalent bonds formed **between the carboxylic acid group of the PAA binder and the hydroxyl group of non-conductive GO** (it was noted that GO used in that study was NOT electrically conductive! (see Page 12765)). In other words, this literature work provides an interesting concept for Si anodes from the viewpoint of binder modification and/or engineering. In stark contrast, however, our work in this manuscript highlights the covalent binding **between Si and the adjacent electrically conductive media**, which, evidently, is a totally different concept for Si anodes.

The authors demonstrate that SF@G, SF@G-HF and SF could form different SEI, which is very interesting. However, the SEI components and distributions in Figure 5h are only deduced from the XPS results, which are not enough. The authors should provide more evidences such as cryo-EM to support the assumption. The current version is not acceptable for publication in Nature Communications. Some detailed comments as following.

R: As for the interfacial difference, **a variety of different characterizations** have been performed in the original version.

Firstly, XPS has been generally used as an effective means to identify the SEI components (e.g., *J. Phys. Chem. C* 2012, 116, 19737 (that is, Ref. 49 in the original version); *J. Power Sources* 2009, 189, 1132 (that is, Ref. 51 in the original version); *J.*

Electrochem. Soc. 2007, 154, A515; *Phys. Chem. Chem. Phys.* 2015, 17, 24956). Based on XPS results (Fig. 5d-f), the distinct difference in SEI components have been explicitly identified. This identification has also been schematically shown in Fig. 5h, with the related text as '*As schematically shown in Fig. 5h, the main interfacial ingredients of cycled SF@G-HF and SF consistently include Li_2CO_3, LiF, Li_xPF_y, $Li_xPO_yF_z$, and R–Si–OR'; the interface of cycled SF@G mostly consists of organic species (e.g., PEO, ROLi, ROCOLi), with an almost negligible amount of inorganic materials.*' in the original version. This difference in major interfacial components is further supported by the interfacial elemental compositions (or atom concentrations) of the cycled samples as shown in Fig. 5g. That is, the obtained elemental compositions based upon interfacial atom concentrations are in full accord with the major interfacial components identified by XPS. Furthermore, for better clarifying the possible origin for this change in SEI, we have provided a schematic illustrating the interface-dependent formation of SEI as Fig. S19, as well as the related reaction mechanisms in Supplementary note 2 in the original version.

Secondly, as described by the text '*The differences discussed above are revealed as well by elemental compositions obtained from EDX analyses (Fig. S23).*' in the original version, this distinct interfacial difference upon cycling has been further certified by investigating elemental compositions of **individual cycled SF@G and SF@G-HF nanoplates** by energy dispersive X-ray spectroscopy (EDX). As shown in the original **Fig. S23**, the elemental compositions and variations acquired by EDX are in good agreement with the XPS results shown in Fig. 5. Specifically, the details have been included in the caption for the original Fig. S23 as '***Figure S23 | a*,*b***, *EDX patterns of cycled SF@G (**a**) w/o and (**b**) w/ HCl washing. **c**,**d**, EDX patterns of cycled SF@G-HF (**c**) w/o and (**d**) w/ HCl washing. **e**, Weight ratios of cycled SF@G and SF@G-HF w/o and w/ HCl washing. **f**,**g**, Comparison of weight ratios between pristine and cycled (**f**) SF@G and (**g**) SF@G-HF w/o and w/ HCl washing. Note that, the relative value of incremental weight ratios of C to O in cycled SF@G and SF@G-HF relative to their pristine counterparts is 4 and 2, respectively. This discrepancy discloses the average stoichiometry of distinct interfacial SEI species in*

both cases as revealed by XPS (Fig. 5d, 5e, and 5g), since the nearly same silicon and carbon components in their pristine counterparts. Furthermore, the significant change in weight ratios of C and O in cycled SF@G-HF upon HCl washing can be associated with its inorganic-dominated interface. In stark contrast, the change in weight ratios of C and O in cycled SF@G upon HCl washing is indistinguishable, attributable to its organic-dominated interface and stable nature. In addition, a slight increase in weight ratios of C and O in cycled SF@G relative to its pristine one suggests the presence of a low amount of SEI components, in good agreement with the SEM observation (Fig. 5a). By comparison, the observed substantial increase in weight ratios of C and O in cycled SF@G-HF indicates the introduction of a large amount of SEI species as exhibited in Fig. 5b.'.

This overwhelming body of experimental evidences has also been recognized by the Referee 3 as '*The authors responded my comments well. Therefore, the paper is acceptable for publication.*'.

Thirdly, the DISTINCT difference in SEI components of SF@G in comparison with their non-covalent counterparts has been consistently documented by both XPS and EDX results, consolidating the conclusion of our manuscript. We believe, here, employing other characterizations, such as cryo-EM as mentioned by the Referee, would only be helpful in elucidating further, but SUBTLE differences, in particular, when modulating the covalent binding types (e.g., Si-O-C, Si-S-C, Si-N-C, and so forth) and numbers, as well as Si/C components of such two-dimensional covalently encapsulated hybrids in the future work.

1. The SF@G displays a very small volume change of 5.8~6.6% (Figure S12), which is very important for the practical applications of Si anodes, the authors should provide more discussions and explanations.

R: We completely agree with the Referee that the very small volume change is essential for the practical applications of Si anodes. In our previous work (*ACS Nano* 2017, 11, 7476-7484; that is, Ref. 27 in the original version), it has been detailed that

every thin Si nanoplates in the SF mimic the behavior of a planar Si thin film in respective spatial orientations upon cycling. Moreover, the free spaces (or gaps) between Si nanoplates can well accommodate the volume change of Si, thus stabilizing the material structure and the electrode architecture (~7% thickness change upon cycling). Resembling the SF, the SF@G possesses a micro-sized flower-like architecture composed of many interconnected nanoplates with different spatial orientations. We can thus firmly assume that the two-dimensional character and three-dimensional spatial orientation of the nanoplates in SF@G (also see Figure 1e and Figure 2a) consistently allow for better accommodation of the volume change of silicon without disturbing the material structure and the electrode architecture. This feature is further corroborated by 5.8~6.6% thickness change of cycled SF@G in the original Fig. S12.

For being even clearer, the related text of '*This can be attributed to the two-dimensional character and three-dimensional spatial orientation of the involved nanoplates*[27].' has been *improved* into '*This can be attributed to the two-dimensional character (mimicking the behavior of a planar thin film) and three-dimensional spatial orientation (creating free spaces to accommodate the volume change) of the involved nanoplates*[27].' in the revised version.

2. What is the thickness of Si nanoplates? Since the authors insist that graphene nanosheets deposited on SF are typically 2~3 layers, however, the C content in SF@G is about 12 % as estimated by thermogravimetric analysis (TGA).

R: The average thickness of the original SF nanoplates is around 7.2 nm, as noted in the original caption of Fig. S7 and also described in our previous work (Ref. 27). As the Referee noticed, the C content in SF@G is about 12% based on EDX and TGA results (Fig. S7c and S10 in the original version). As depicted in the original caption of Fig. S7, based on the atomic ratio of C, O and Si obtained by EDX as well as the layer number of graphene observed from TEM images, it can be tentatively assumed that, in an SF@G nanoplate, two graphene nanosheets are covalently bound through oxygen atoms to BOTH sides of a Si nanoplate with a thickness of 6.9 nm (Fig. S7d),

corresponding to a possible molecular formula of $C_{64}O_{16}Si_{192}$. Notably, the thus-deduced thickness of 6.9 nm is very close to the average thickness (7.2 nm) of the original SF nanoplates. Consistently, we have noted this in the original version as '*The covalent binding at the interface of two-dimensional components can be described by a tentative chemical structure of SF@G (Fig. S7).*'.

3. In Figure 4a, the SF@G and SF@G-HF have capacities about 2200 mAh g^{-1}, however, in the long-term cycle in Figure 3c, their capacities are below 2000 mAh g^{-1}, why?

R: Thank very much for the Referee's kind note. As the Referee noticed, the average capacity values are approximately 2000 and 2200 mAh g^{-1} in the Fig. 3c and the Fig. 4a, respectively. This discrepancy can be associated with the testing (or cycling) conditions. Firstly, as described in the original version, in comparison with Fig. 4a, there is no ten cycles of 0.8 A g^{-1} before long-term cycling at 2 A g^{-1} in Fig. 3c. Cycling at different current rates may result in differences for activating the material, which can be relevant for the observed discrepancy. Secondly, since all the electrochemical measurements were undertaken at room temperature, the pre-cycling conditions (e.g., ten cycles of 0.8 A g^{-1} before 2 A g^{-1}) incur the temperature difference inside a cell, which can be another factor for the discrepancy. It has been reported that the capacity fluctuation can be associated with the temperature variation (e.g., Ref. 18, Ref. 22).

Consistent with the above discussion, the related text of '*the capacity reported was based on the total weight of active materials in the working electrode.*' has been *improved* into '*the capacity reported was based on the total weight of active materials in the working electrode, as well as the annotated cycling conditions.*' in the revised version. For being more accurate, the text of '*Capacity of SF@G, SF@G-HF, and SF cycled at different rates from 0.8 to 20 A* g^{-1}.' has been *improved* into '*Capacity of SF@G, SF@G-HF, and SF cycled at different rates from 0.8 to 20 A* g^{-1} *(ten cycles for each rate).*' in the revised version.

4. Line 129-130, SF@G also offers a dramatically improved cycling stability at a high rate 130 of 2 A g-1 over 500 cycles compared to SF@G (here, the SF@G should be changed as SF@G-HF) and SF.

R: Thank very much for the Referee's kind reminder. The text of '*SF@G also offers a dramatically improved cycling stability at a high rate of 2 A g^{-1} over 500 cycles compared to SF@G and SF,*' has been *modified* into '*SF@G also offers a dramatically improved cycling stability at a high rate of 2 A g^{-1} over 500 cycles compared to SF@G-HF and SF,*' in the revised version.

5. If the author investigated the effect of morphology and structure of graphene coating on the electrochemical properties of SF@G? the Raman results reveal the the ratio of D band to G band (ID/IG) to be around 1.5, if this is the optimized structure of graphene coating?

R: As extensively investigated (e.g., Ref. 9), different structures and morphologies of graphene have been demonstrated to be a powerful factor for performance modulation of silicon and others. We completely agree with the Referee that the material components, e.g., the morphology and structure of graphene coating, have effects on the hybrid electrochemical properties. For this, the graphene employed in our study has been characterized and described as '*On the surface of every nanoplate, graphene nanosheets (typically, 2~3 layers) are observed to be conformally deposited, in line with investigations of tap density, specific surface area, pore size distribution, as well as graphene morphology (Fig. S2, S3, and S4).*' and '*Furthermore, the ratio of the D band to G band is estimated to be around 1.5, revealing the presence of pinholes, defects and/or disordered domains in the deposited graphene favorable for ion transport (Fig. 2g, S5).*' in the original version. With the material components as described above, our work in this manuscript distinctly focuses on two-dimensional covalent encapsulation, in which the two-dimensional covalent binding creates a robust and efficient contact between the silicon and electrically conductive media, enabling stable and fast electron as well as ion transport from and to silicon. As thoroughly evidenced by comparing SF@G, SF@G-HF, and SF in the original version,

this study represents a proof-of-concept of a new material design, and opens a new, broad avenue to stabilize silicon without sacrificing other device parameters. We are, thus, confident that more two-dimensional covalent encapsulation studies, e.g., based on diverse material components *via* structural, textural, and morphological modulations of graphene, will hold enormous promise in the foreseeable future.

6. The SF@G exhibits larger b values in Figure 4g, however, the SF@G-HF and SF have the similiar b values. The authors should provide more explanations since the SF@G-HF and SF@G have the similar morphology and structure except Si-O-C bonding.

R: Thank very much for the Referee's kind reminder. As displayed in the original Figure 4g and also pointed out by the Referee, the SF@G-HF has a b value close to that of SF, although the SF@G-HF bears a similar morphology to that of SF@G. This observation suggests the similar interfacial contact between the silicon and adjacent electrically conductive media in the cycled SF@G-HF and SF (b and c in the original Figure S19), which is further evidenced by the similar interfacial morphology and chemical composition of cycled SF@G-HF and SF (the original Figure 5).

Consistent with the above discussion, the text of '*It should be noted as well that the similar b values of SF@G-HF and SF suggest the comparable interfacial contact between the silicon and adjacent electrically conductive media in both cases upon cycling.*' has been *added* into Supplementary note 1 in the revised supporting information.

7. What is the tap density of SF@G? In Table S1, the electrode press density of SF@G is only 0.94 g cm-3.

R: The tap density of SF@G is around 0.9 g cm^{-3}. As exhibited and depicted in the original Figure S2, we have noted that '*the density of SF@G is approximately four times that of Si NP, being evidently favorable for volumetric capacity and critical for practical applications.*'. Furthermore, both the tap density and press density of SF@G can be associated with the two-dimensional character and three-dimensional spatial

orientation of the involved nanoplates as discussed in our previous work (Ref. 27), which rather allow better accommodation of the volume change of silicon in SF@G. For being clear, the text of '*The SF@G shows a similar tap density to that of SF,*' has been *improved* into '*The SF@G shows a similar tap density (ca. 0.9 g cm^{-3}) to that of SF,*' in the revised supporting information.

8. The um should be corrected as μm in main text and supporting informations.

R: The 'um' in the revised main text and supporting information has been *checked* and/or *corrected* as 'μm'.

REVIEWERS' COMMENTS:

Reviewer #4 (Remarks to the Author):

The authors addressed the reviewer's comments.

Responses to the Referee's comments

REVIEWERS' COMMENTS:

Reviewer #4 (Remarks to the Author):

The authors addressed the reviewer's comments.

R: Thanks again for the Referee's fruitful comments and invaluable time, as well as recognition of our work.
